# Pentannulation of N-heterocycles by a tandem gold-catalyzed [3,3]-rearrangement/Nazarov reaction of propargyl ester derivatives: a computational study on the crucial role of the nitrogen atom

**DOI:** 10.3762/bjoc.16.255

**Published:** 2020-12-15

**Authors:** Giovanna Zanella, Martina Petrović, Dina Scarpi, Ernesto G Occhiato, Enrique Gómez-Bengoa

**Affiliations:** 1 Departamento de Química Orgánica I, University of the Basque Country, UPV-EHU, Manuel Lardizabal 3, 20018 Donostia, San Sebastián, Basque Country, Spain; 2 Dipartimento di Chimica “U. Schiff”, Università degli Studi di Firenze, Via della Lastruccia 13, 50019 Sesto Fiorentino, Florence, Italy

**Keywords:** DFT calculations, gold catalysis, Nazarov reaction, N-heterocycles

## Abstract

The tandem gold(I)-catalyzed rearrangement/Nazarov reaction of enynyl acetates in which the double bond is embedded in a piperidine ring was computationally and experimentally studied. The theoretical calculations predict that the position of the propargylic acetate substituent has a great impact on the reactivity. In contrast to our previous successful cyclization of the 2-substituted substrates, where the nitrogen favors the formation of the cyclized final product, the substitution at position 3 was computed to have a deleterious effect on the electronic properties of the molecules, increasing the activation barriers of the Nazarov reaction. The sluggish reactivity of 3-substituted piperidines predicted by the calculations was further confirmed by the results obtained with some designed substrates.

## Introduction

In the development of new and effective catalysts, step economy is surely one of the major goals. A reduction of the number of steps in the synthesis of complex compounds can be attained by cascade reactions, which allow for structural modifications on the organic compounds by forming several chemical bonds in one pot. To this end, gold catalysis [1–7] has been widely exploited to construct various cyclic and heterocyclic frameworks through cascade reactions triggered by the activation of a triple bond, which has ultimately led to the total synthesis of several natural compounds [2,8]. The gold-catalyzed rearrangement of suitably substituted propargylic esters in particular provides a platform for cascade processes that involve a cationic or an allene intermediate generated in the first step [1,9–12].

**Figure 1:**
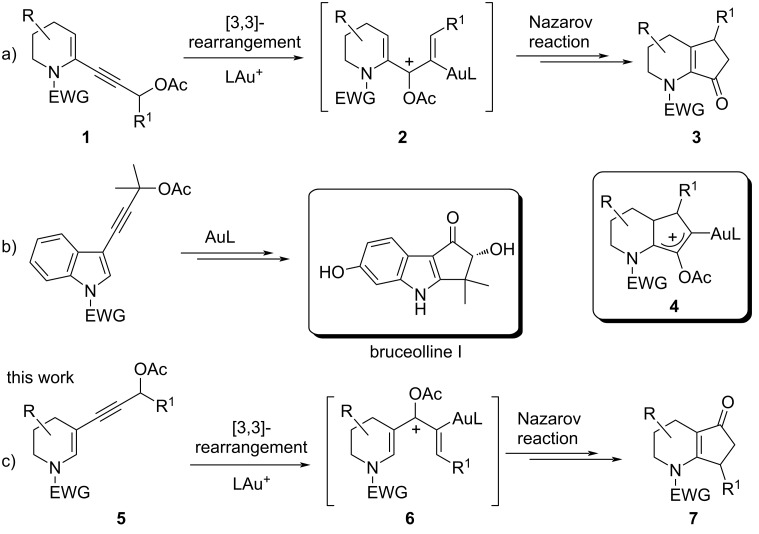
Tandem acetate rearrangement/Nazarov cyclization of different substrates.

In the framework of our studies on gold(I)-catalyzed reactions of propargyl alcohol derivatives [13–15], we have recently reported that the pentannulation of N-heterocycles [16] can be efficiently achieved by a cascade gold-catalyzed [3,3]-rearrangement/Nazarov reaction of propargyl ester derivatives (Figure 1a) [17–24], and we have exploited such a methodology for the synthesis of bruceollines H and I from 3-substituted indoles (Figure 1b) [25–26]. Our computational study showed that the Nazarov reaction is fast with the 2-substituted piperidine derivatives **1** because of the accelerating effect of the nitrogen atom that stabilizes the oxyallyl cation intermediate **4** formed upon the ring closure. This was in analogy to that found for the classical Brønsted or Lewis acid-catalyzed Nazarov reaction involving N-heterocycles [27–37] and in accordance with the polarized Nazarov reaction concept developed by Frontier [27,33].

In an effort to broaden the scope of the reaction and the diversity of products, we assumed that the N-heterocycles **5**, bearing the propargyl side chain at C3, would deliver a cyclopenta-fused heterocyclic system with an alternate position of the C=O group on the five-membered ring when treated with gold(I) (see **7**, Figure 1c). In this context, the Nazarov cyclization has been profusely studied, and it was found that it is very sensitive to the electronic features of the substrates. For example, the rate is optimal in polarized systems obtained by the proper introduction of electronically asymmetric fragments [38–39]. Thus, an unsuitable combination of substituents can be detrimental for the reactivity, and we were aware that the electron donor nitrogen in **5** (having a side chain at C3) could stabilize the pentadienyl cationic intermediate **6**, and thus relenting the 4π-electrocyclization, causing either the degradation of the starting material or the formation of unwanted side products. In fact, preliminary experimental results with **5** pointed in this direction, and we decided to carry out a complete computational analysis to evaluate the entire reaction profile and to help us validate our hypothesis before embarking on a potential total synthesis, involving such a process, in the future. In parallel, a few suitable substrates were also subjected to gold catalysis with the aim of verifying the conclusions drawn by the calculations.

## Results and Discussion

### Computational methods

**Figure 2:**
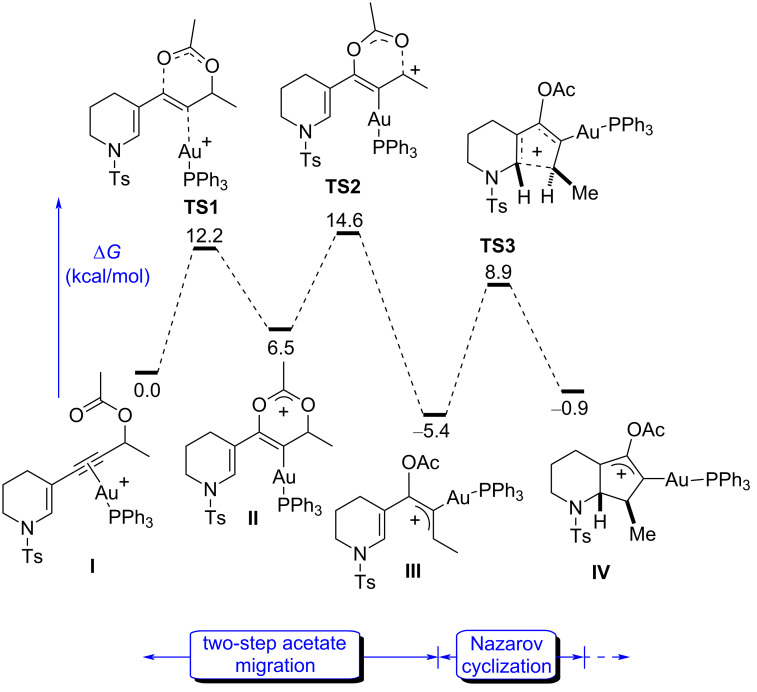
DFT-computed energy profile of the tandem Au(I)-catalyzed [3,3]-rearrangement/Nazarov reaction of 3-substituted piperidine derivatives.

In order to identify the structures and the energies of the critical steps of the mechanism, the potential reaction coordinates of the whole tandem [3,3]-rearrangement/Nazarov cyclization were studied computationally (Figure 2). To this end, a model substrate bearing *p*-toluensulfonyl as the protecting group on the nitrogen atom was chosen owing to the compatibility with such a process [16]. The structures were located using the B3LYP density functional theory method as implemented in the Gaussian suite of programs, using the 6-31G(d,p) basis set for nonmetallic atoms and SDD for Au. The alkynyl–gold(I) cationic complex **I** (Figure 2) was considered as the starting point of the mechanism (Δ*G* = 0 kcal⋅mol^−1^), and all reported energy values in the following discussion are relative to this figure. The values for Δ*G* correspond to the Gibbs energy computed at the M06/def2tzvpp level of theory in a solvent model (IEFPCM, solvent = DCM). The intrinsic reaction coordinates (IRC) were followed to verify the energy profiles connecting the key transition structures to the correct associated local minima. Ph_3_P was chosen as the ligand in analogy to the previous calculations on compound **1** [16].

### Computational discussion

In analogy to similar processes [11,21–22,40–41], the reaction is initiated by a two-step [3,3]-acetate rearrangement [42], triggered by the coordination of the cationic gold species to the alkyne **5**, as in **I** (Figure 2). The first step **TS1** has a low activation energy (Δ*G*^‡^ = 12.2 kcal⋅mol^−1^) to form the unstable cyclic intermediate **II**. This short-lived species rapidly reopens through **TS2** (ΔΔ*G*^‡^ = 8.1 kcal⋅mol^−1^) to give the pentadienyl cation **III**, which presents a high stability (Δ*G* = −5.4 kcal⋅mol^−1^), and thus making the Nazarov cyclization through **TS3** an endergonic process (from **III** to **IV**). In fact, the energy values calculated in Figure 2 show that either **TS2** or **TS3** or a combination of the two, depending on the reaction conditions, could be rate determining as they share very similar numbers, 14.6 kcal⋅mol^−1^ (from **I** to **TS2**) and 14.3 kcal⋅mol^−1^ (from **III** to **TS3**), respectively. We also computed the following steps of deprotonation, protodeauration, and acetate hydrolysis, which would lead to the final product **7**, showing that they are not critical for the rate and outcome of the reaction. Thus, they will be discussed later separately.

**Figure 3:**
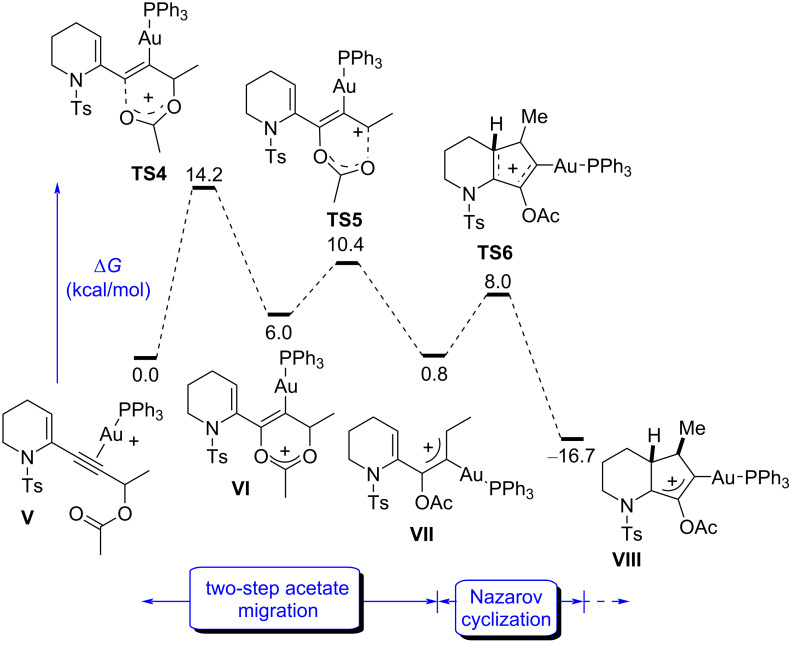
DFT-computed energy profile of the tandem Au(I)-catalyzed [3,3]-rearrangement/Nazarov reaction of 2-substituted piperidine derivatives.

Confirming our working hypothesis, this set of initial data contrasts with the computed gold(I)-catalyzed [3,3]-rearrangement/Nazarov reaction of **1**. We had previously shown that for 2-substituted analogs of **1** (NCO_2_Me), the acetate rearrangement (specially the **TS1**-like first step). is rate determining with a low activation barrier of 10.0 kcal⋅mol^−1^ and that the Nazarov-cyclization is an extremely easy process (ΔΔ*G*^‡^ = 5.1 kcal⋅mol^−1^) [16]. To homogenize with our results in Figure 1, we computed the corresponding *N*-sulfonyl-protected derivative **1** (Figure 3), confirming the differences that the 2- and 3-substitution, respectively, exert in the reaction outcome. Starting with **V**, the acetate rearrangement is rate determining (ΔΔ*G*^‡^ = 14.2 kcal⋅mol^−1^), and more importantly, the activation energy for the cyclization in **TS6** is very low (ΔΔ*G*^‡^ = 7.2 kcal⋅mol^−1^) and highly exergonic (ΔΔ*G*^‡^ = −17.5 kcal⋅mol^−1^), making the process from **VII** to **VIII** completely irreversible. In contrast, the 3-substituted intermediate **III** gives a much slower and reversible process.

**Figure 4:**
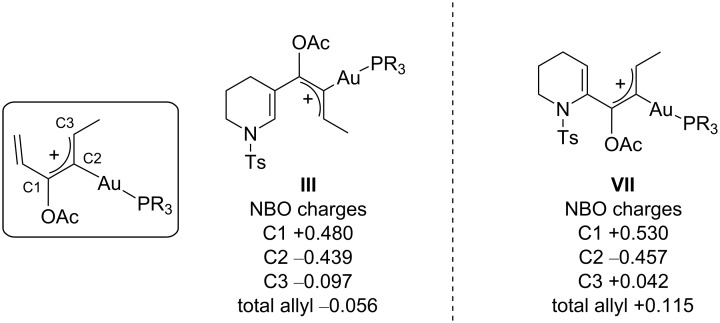
Computed comparison of the NBO charges of 2- and 3-substituted substrates.

Thus, the main reason for the worse performance of **5** as a substrate seems to be related to the higher stability of the intermediate **III**, which could be attributed to the π-donating ability of the nitrogen atom to stabilize the positive charge [43]. We evaluated this effect by calculating the charges through “natural bond orbital analysis” (NBO) of the atoms of the intermediate **III** and the 2-substitued analogue **VII** and found a significant difference between the two (Figure 4).

Indeed, the total allyl charge on the 3-substituted intermediate **III** results to be almost neutral or even slightly negative (−0.056 e), confirming that the lone pair of the nitrogen atom can stabilize the positive charge of the allyl system by conjugation, affecting the following cyclization reaction. Meanwhile, the 2-substitued analogue **VII** does not present a conjugated system, and the total allyl charge cannot be stabilized, maintaining a positive value (+0.115). As a result, **VII** seems to be much more reactive and the associated cyclization much more exergonic than for **III**. The carbon atoms C1 and C3 seem to be especially more positive in **VII** than in **III**.

A second obvious difference between the two isomeric pathways in Figure 2 and Figure 3 is the much higher relative stability of the cyclized structure **VIII** compared to the analogue **IV** (−16.7 vs −0.9 kcal⋅mol^−1^). The π-donating ability of the nitrogen atom might have a clear stabilizing effect in **VIII**, while the nitrogen and the cationic allyl system are disconnected in **IV**. This effect is reflected in the corresponding transition states, with **TS3** being higher in energy than **TS6**.

**Figure 5:**
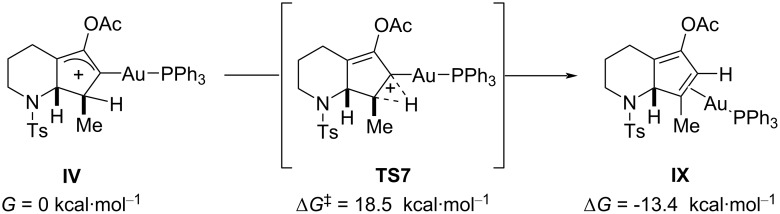
Single-step transformation of **IV** to **IX**.

**Figure 6:**
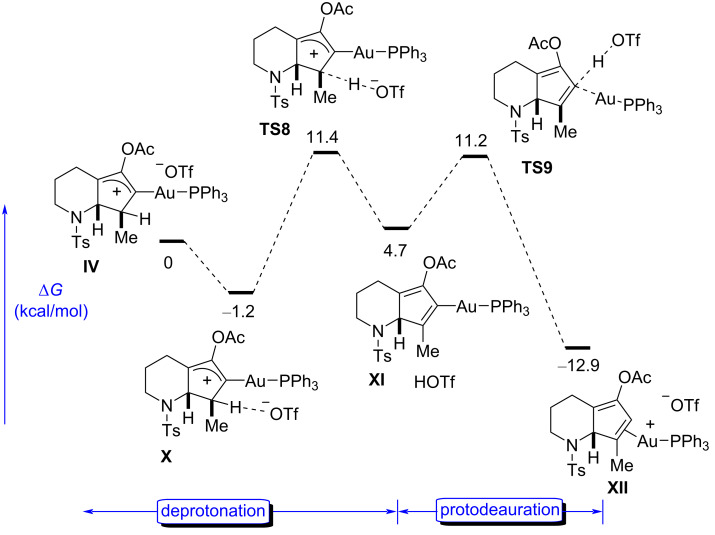
Triflate-promoted hydrogen abstraction and protodeauration with HOTf.

As mentioned before, after the slow cyclization step in **TS3**, we focused our analysis on the transformation of the bicyclic intermediate **IV** to the final diene product. Basically, the final steps have to include a deprotonation, protodeauration, and in some cases acetate hydrolysis. These steps can occur through different pathways; in particular, we considered a single-step intramolecular hydride shift with concomitant C–Au-bond breaking (Figure 5) or a base-mediated deprotonation, followed by Au–C-bond hydrolysis through protodeauration (Figure 6). In the former case, it emerged that the 1,2-hydrogen shift in **TS7** is quite high in energy (Δ*G*^‡^ = 18.5 kcal⋅mol^−1^) relative to the previous barriers shown in Figure 2. This barrier is also much higher than the traditional 1,2-hydride shift in carbocations, which usually show barriers even under 10 kcal⋅mol^−1^. It has been suggested that the presence of water can catalyze this reaction (proton-transport catalysis strategy) through a two-step deprotonation/protonation process [11,21,41–42,44], but in our study, preliminary calculations in the presence of water did not improve the results in Figure 5.

Therefore, we focused on the proton abstraction pathway. Several possible bases exist in the reaction medium, such as the counterion in the gold(I) salt, the anion participating in the silver salt coadditives, or water. In analogy to our previous work [16], we initially modelled the deprotonation step with the triflate anion as a base (Figure 6).

In fact, the abstraction of the hydrogen atom in the position adjacent to Au (C3) shows a low activation barrier (**TS8**, Δ*G*^‡^ = 12.6 kcal⋅mol^−1^) from the corresponding precomplex, leading to the formation of the intermediate **XI** and triflic acid. The high acidity of the latter facilitates the protodeauration in the last step (**TS9**), which occurs exothermically and with a barrier of only 6.5 kcal⋅mol^−1^. The easiness of the two-step process from **IV** to **XII** is remarkable given the low basicity of the triflate anion, suggesting that other possible anions present in the medium could also play the same role. Comparing the different pathways in Figure 5 and Figure 6, it emerged that the base-mediated process is clearly favored over the 1,2-H-shift.

**Figure 7:**
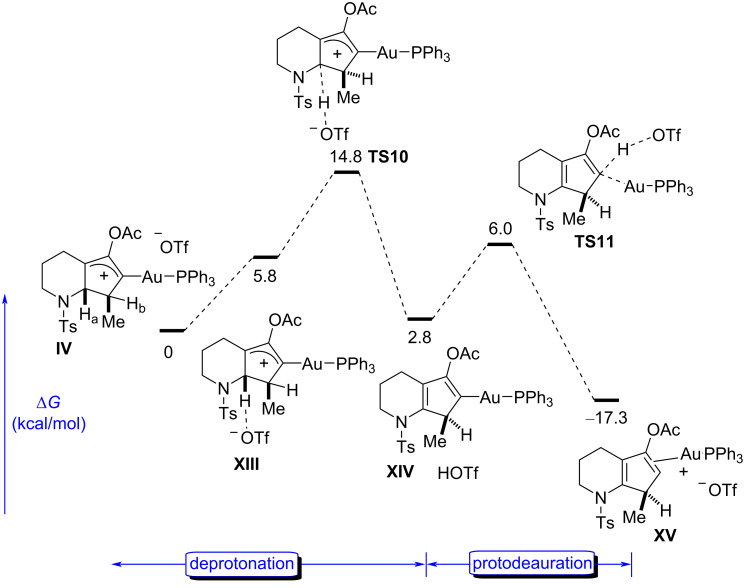
Triflate-mediated abstraction of the hydrogen atom H_a_ and protodeauration.

We were also aware of the regioselectivity issue that arose during the deprotonation due to the presence of two similar hydrogen atoms (H_a_ and H_b_) in **IV** (Figure 7), and we wondered if there were significant differences between the two pathways. Indeed, the deprotonation of H_a_ does not seem as easy as H_b_, despite the fact that the final product **XV** is a conjugated dienamine and more stable (ΔΔ*G* = 4.4 kcal⋅mol^−1^) than **XII**, which lacks the conjugation. However, according to the energy profile, this observation does not have a reflection in the deprotonation step, which seems to be affected partially by the steric hindrance around the two hydrogen atoms, being clearly higher in H_a_ (a 2.2 kcal⋅mol^−1^ higher activation energy of **TS10** than for **TS8**). Thus, under kinetic control, the reaction would lead to the formation of **XII**. However, as will be commented on later in the discussion, the experimental results clearly show the sole formation of compound **15** (Table 1), which arises from hydrolysis of an intermediate related to the complex **XV**. Thus, we believe that the higher thermodynamic stability of **XV** (4.4 kcal⋅mol^−1^ lower than for **XII**), which is due to the conjugation of the nitrogen atom and the diene system, accounts for the preferential formation and the consequent formation of **15**. It cannot be overlooked that the formation of the intermediates **XII** and **XV** is hardly reversible due to the high exergonic character, and thus the equilibration of both final isomers through the previous intermediate **IV** is very unlikely. Our hypothesis is that an isomerization between **XII** and **XV** must be operative under these reaction conditions through a nonstudied protonation/deprotonation sequence. Finally, although we did not study in detail the acetate hydrolysis from **XV** to **15**, we could confirm the higher stability (by more than 6 kcal⋅mol^−1^) of **15** relative to the enone isomer arising from **XII**, in agreement again with the experimental results.

**Table 1:** Gold(I)-catalyzed [3,3]-rearrangement/Nazarov reaction of **14**.^a^

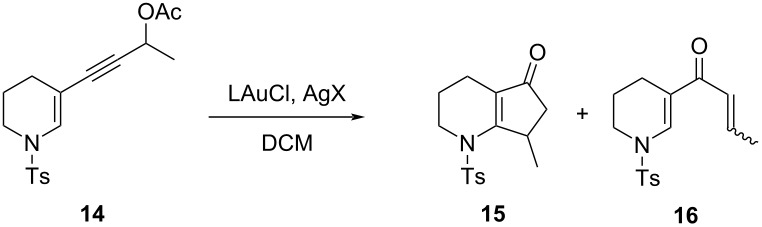					

entry	ligand	anion	time (h)	**15** (%)^b^	**16** (%)

**1**	PPh_3_	TfO	16	51	24
**2**	PPh_3_	SbF_6_	6	66	^c^
**3**	PCy_3_	SbF_6_	6	10^d^	–
**4**	P(4-CF_3_C_6_H_4_)_3_	SbF_6_	6	49	^c^
**5**^e^	PPh_3_	SbF_6_	1	<5^d^	–
**6**^f^	PPh_3_	SbF_6_	4	48	^c^

^a^Reaction conditions: 0.15–0.2 mmol of **14**, 5 mol % of the catalyst, prepared by adding the silver salt to a 0.004 M solution of gold(I) chloride in DCM. The solvent was not dried before use unless otherwise indicated. ^b^Yield after chromatography unless otherwise indicated. ^c^Detected by ^1^H NMR analysis of the crude reaction mixture. ^d^Conversion measured by ^1^H NMR. ^e^Using dry DCM. ^f^Reaction carried out in refluxing solvent.

### Experimental discussion

**Scheme 1:**
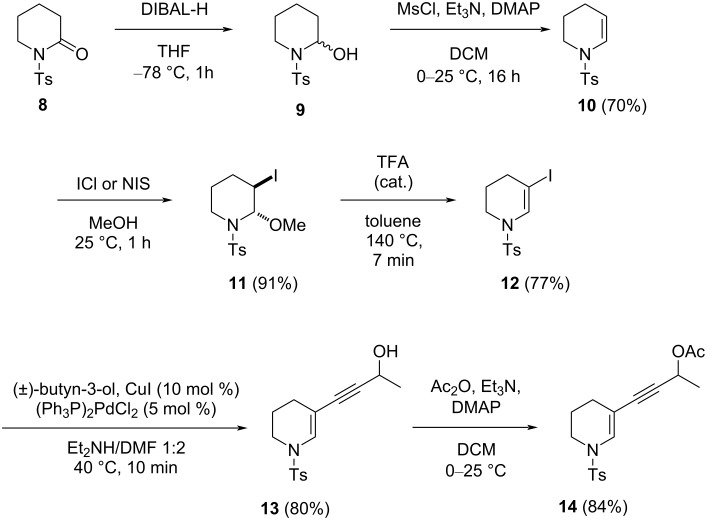
Synthesis of the enynyl acetate starting material **14**.

As a summary of the previous discussion, we rationalized that the high stability of the intermediate **III** (especially compared to **VII**) and the relatively high activation energy of the cyclization in **TS3** (vs the easier cyclization of **TS6**) could hamper the reactivity of 3-substituted piperidines, and that the slow cyclization of the intermediate **III** could result in starting material degradation or appearance of unwanted side reactions. To assess this hypothesis from an experimental point of view, the synthesis of the model compound **14** used in the calculation (as a gold complex **I**) was carried out and then subjected to gold catalysis. The synthesis started with the reduction of the *N*-Ts δ-valerolactam **8** with DIBAL-H into the corresponding lactamol **9** (Scheme 1), which was transformed into the enesulfonamide **10** in 70% yield by mesylation, followed by base-induced elimination of methanesulfonic acid, as previously reported [45]. In the next step, the electrophilic addition of iodine monochloride to the double bond of the enesulfonamide **10**, followed by a nucleophilic attack of methanol on the formed iodonium ion afforded the α-methoxy-β-iodopiperidine **11** as a single stereoisomer (91% yield) [46]. The treatment of **11** with a catalytic amount of trifluoroacetic acid in toluene at 140 °C for 7 min led to the elimination of methanol and provided the 3-iodoenesulfonamide **12** in 77% yield [47]. To avoid the use of these harsh reaction conditions, we employed other methods, but both the iodination and bromination of **10** proved to be more difficult than anticipated. For example, attempts to obtain the 3-iodo derivative **11** using I_2_/Cs_2_CO_3_ in dioxane [48], NIS in DMF [49], NIS/AgNO_3_ in acetonitrile [50], and NIS/TFA in DCM [51] failed completely or provided the desired product as a complex mixture with unknown products. Then, the iodoenesulfonamide **12** was coupled with (±)-butyn-3-ol under Sonogashira conditions [52] to afford the enynyl alcohol **13**, which was treated with acetic anhydride to provide the enynyl acetate **14** in a yield of 67% over two steps.

Then, we applied the typical conditions for the [3,3]-rearrangement/Nazarov cyclization that we used for enynyl acetates of the type **1** (Figure 1) [16] to the enynyl acetate **14**, i.e., 5 mol % Ph_3_PAuCl/AgOTf in DCM at room temperature (Table 1, entry 1) and 5 mol % of Ph_3_PAuCl/AgSbF_6_ in the same solvent (Table 1, entry 2), which were the best conditions that we tested in the rearrangement of the enynyl acetates **1** (e.g., with R = H, R^1^ = Me, EWG = Ts, the total yield of the Nazarov products was 85% after chromatography using AgSbF_6_ as the counter ion source, and with R = H, R^1^ = *n*-Bu, and EWG = Ts, the total yield was 86% when using AgOTf as the silver salt). Under both conditions, the reaction of **14** led to the formation of the cyclopentenone **15** in a lower yield (51% and 66%, respectively), and in comparison to the gold(I)-catalyzed reaction of the enynyl acetates **1**, it was much slower with both catalytic systems (6–16 h vs 1.5–2 h for the complete disappearance of the starting material). Moreover, besides the ketone **16** [53], formed as byproduct in the reaction with AgOTf, we observed the formation of many other unidentified compounds, reasonably either via side reactions of gold intermediates or the degradation of the starting enynyl acetate **14**.

In order to increase the reaction rate and decrease the amount of side products, the best reaction conditions (with AgSbF_6_) were modified by using different precatalysts (Table 1, entries 3 and 4), a dry solvent (Table 1, entry 5), and the reaction was also carried out at a higher temperature (Table 1, entry 6). However, none of these attempts were met with success, and indeed, a very sluggish reactivity was recorded in all these cases. Unfortunately, these results confirmed the negative predictions arising from the above calculations, but at the same time, they serve as a validation of the accuracy of the computational method we have used for the comparison of the isomeric complexes **I** and **V**.

**Scheme 2:**
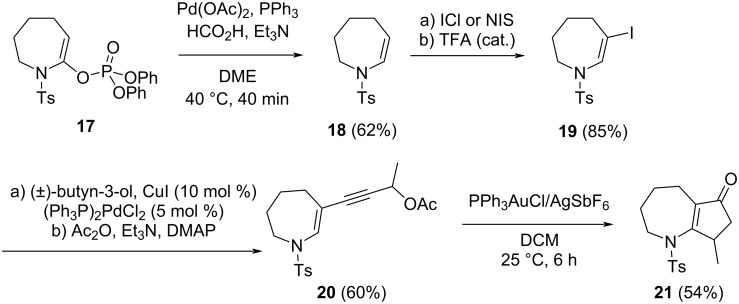
Synthesis and cyclization of enynyl acetate **20**.

Our previous experience in this area taught us that seven-membered azepane-derived enynyl acetates react faster than the corresponding piperidine analogues **1**, prompting us to prepare enynyl acetate **20** as reported in Scheme 2. We wanted to confirm the negative effect of the substitution at the 3-position of the ring. We intended to follow a similar strategy to that outlined in Scheme 1, but the two-step procedure from **8** to **10** failed with the seven-membered ring. Thus, the enesulfonamide **18** was prepared via the palladium-catalyzed reduction of the corresponding phosphate **17** [54]. Iodination and Sonogashira coupling, followed by acetylation led to the formation of the desired enynyl acetate **20**. This compound was treated with 5 mol % Ph_3_PAuCl/AgSbF_6_ in DCM, and after 6 h, this afforded the cyclopenta-fused product **21** in 54% yield. Again, the reaction was very slow compared to the corresponding 2-substituted azepane derivative and provided many unidentified side products, reducing our interest in the process.

## Conclusion

In summary, we computationally studied and experimentally verified the [3,3]-rearrangement/Nazarov cyclization of 2,3-dehydropiperidines substituted with a propargyl acetate group in the 3-position, demonstrating the significance of the correct positioning of the nitrogen atom relative to the forming cycle. The comparison of the reactivity of the substrate having the piperidine ring substituted at the 2- vs the 3-position was very instructive about the optimal electronic features of the reactive species. In this regard, the initial rearrangement of the propargyl acetate induces the formation of a divinyl cationic intermediate, which is differently stabilized by conjugation with the nitrogen atom depending on the relative position of nitrogen. For the 3-substitution, the π-donor ability of the nitrogen atom strongly stabilizes the intermediate, reducing the reactivity. NBO calculations have also been used to confirm this hypothesis. We present some experimental data corroborating the sluggish reactivity of the 3-substituted substrates, in comparison to the 2-substituted analogues that we have previously described.

## Supporting Information

File 1 Computational section, experimental section, and NMR spectra.
