# Supplementary material for: Pentannulation of N-heterocycles by a tandem gold-catalyzed [3,3]-rearrangement/Nazarov reaction of propargyl ester derivatives: a computational study on the crucial role of the nitrogen atom
Source: Beilstein J Org Chem. 2020 Dec 15;16:3059–68. doi: 10.3762/bjoc.16.255 (PMC7753109; doi:10.3762/bjoc.16.255)
Supplement: File 1 — Computational section, experimental section, and NMR spectra. [file Beilstein_J_Org_Chem-16-3059-s001.pdf]

BEILSTEIN JOURNAL OF ORGANIC CHEMISTRY

Supporting Information

for

Pentannulation of N-heterocycles by a tandem gold-catalyzed [3,3]-rearrangement/Nazarov reaction of propargyl ester derivatives: a computational study on the crucial role of the nitrogen atom

Giovanna Zanella, Martina Petrović, Dina Scarpi, Ernesto G. Occhiato
and Enrique Gómez-Bengoa

Beilstein J. Org. Chem. **2020,** *16,* 3059–3068. doi:10.3762/bjoc.16.255

Computational section, experimental section, and NMR spectra

License and Terms: This is a supporting information file under the terms of the Creative Commons Attribution License (https://creativecommons.org/licenses/by/4.0). Please note that the reuse, redistribution and reproduction in particular requires that the author(s) and source are credited and that individual graphics may be subject to special legal provisions.
The license is subject to the *Beilstein Journal of Organic Chemistry* terms and conditions: (https://www.beilstein-journals.org/bjoc/terms)

Index

Computational section **S2**

Experimental section **S62**

^{1}H and ^{13}C NMR spectra **S67**

Computational section

All structures were initially optimized using density functional theory (DFT) with B3LYP[1] and the 6-31G(d,p) basis set and SDD[2] for Au as implemented in Gaussian 16.[3] Final energies were calculated at the M06[4]/def2tzvpp[5] level of theory, in a solvent model (IEFPCM, solvent = dichloromethane).[6] The intrinsic reaction coordinates (IRC)[7] were followed to verify the energy profiles connecting the key transition structures to the correct associated local minima. The stationary points were characterized by frequency calculations in order to verify that they have the right number of imaginary frequencies. The calculation of the atomic charges of the intermediate **III** was carried out through "natural bond orbital analysis".[8]

[1] (a) C. Lee, W. Yang and R. G. Parr, *Phys. Rev. B*, **1988**, *37*, 785-789; (b) A. D. Becke, *J. Chem. Phys.*, **1993**, *98*, 5648-5652; (c) W. Kohn, a. D. Becke and R. G. Parr, *J. Phys. Chem*., **1996**, *100.* 12974-12980.

[2] (a) M. Dolg, U. Wedig, H. Stoll, and H. Preuss, *J. Chem. Phys.* **1987**, *86*, 866. (b) D. Andrae, U. Haussermann, M. Dolg, H. Stoll and H. Preuss, *Theor. Chim. Acta* **1990.** *77*, 123.

[3] Gaussian 16, Revision A.03, Frisch, M. J.; Trucks, G. W.; Schlegel, H. B.; Scuseria, G. E.; Robb, M. A.; Cheeseman, J. R.; Scalmani, G.; Barone, V.; Petersson, G. A.; Nakatsuji, H.; Li, X.; Caricato, M.; Marenich, A. V.; Bloino, J.; Janesko, B. G.; Gomperts, R.; Mennucci, B.; Hratchian, H. P.; Ortiz, J. V.; Izmaylov, A. F.; Sonnenberg, J. L.; Williams-Young, D.; Ding, F.; Lipparini, F.; Egidi, F.; Goings, J.; Peng, B.; Petrone, A.; Henderson, T.; Ranasinghe, D.; Zakrzewski, V. G.; Gao, J.; Rega, N.; Zheng, G.; Liang, W.; Hada, M.; Ehara, M.; Toyota, K.; Fukuda, R.; Hasegawa, J.; Ishida, M.; Nakajima, T.; Honda, Y.; Kitao, O.; Nakai, H.; Vreven, T.; Throssell, K.; Montgomery, J. A., Jr.; Peralta, J. E.; Ogliaro, F.; Bearpark, M. J.; Heyd, J. J.; Brothers, E. N.; Kudin, K. N.; Staroverov, V. N.; Keith, T. A.; Kobayashi, R.; Normand, J.; Raghavachari, K.; Rendell, A. P.; Burant, J. C.; Iyengar, S. S.; Tomasi, J.; Cossi, M.; Millam, J. M.; Klene, M.; Adamo, C.; Cammi, R.; Ochterski, J. W.; Martin, R. L.; Morokuma, K.; Farkas, O.; Foresman, J. B.; Fox, D. J. Gaussian, Inc., Wallingford CT, **2016**.

[4] Y. Zhao and D. G. Truhlar, *Theor. Chem. Acc.*, **2008**, *120.* 215-241.

[5] (a) Weigend, F.; Furche, F. and Ahlrichs, R. *J. Chem. Phys.* **2003**, *119*, 12753-12762; (b) F. Weigend and R. Ahlrichs, *Phys. Chem. Chem. Phys.* **2005**, *7*, 3297-3305

[6] (a) E. Cancés, B. Mennucci and J. Tomasi, *J. Chem. Phys.*, **1997**, *107*, 3032-3041; (b) M. Cossi, V. Barone, B. Mennucci and J. Tomasi, *Chem. Phys. Lett.*, **1998**, *286*, 253-260; (c) J. Tomasi, B. Mennucci and E. Cancès, *J. Mol.Struc.: THEOCHEM*, **1999**, *464*, 211-226.

[7] Gonzalez, C.; Schlegel, H. B. *J. Phys. Chem.* **1990.** *94*, 5523-5527.

[8] NBO Version 3.1, E. D. Glendening, A. E. Reed, J. E. Carpenter, and F. Weinhold.

I

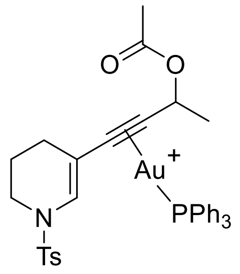

G at M06/def2tzvpp (IEFPCM, dichloromethane) = −2.623,160553 Hartree
Correction at B3LYP/6-31G(d,p) = 0.548345
Cartesian coordinates of the computed structure

Center Number	Atomic Number	Atomic type	Coordinates (Angstroms)		
			X	Y	Z
1	6	0	-2,144945	-1,481444	-1,276341
2	6	0	-3,138787	-1,136409	-0.349069
3	1	0	-2,057901	-0.414584	-3,149527
4	6	0	-2,435219	-1,371908	-2,766363
5	6	0	-4,727849	-0.507615	-2,125108
6	6	0	-3,946087	-1,479122	-3,013236
7	1	0	-5,800646	-0.695144	-2,195203
8	1	0	-4,18532	-1,260693	-4,057942
9	1	0	-4,535449	0.533763	-2,407757
10	1	0	-4,285753	-2,50009	-2,807735
11	1	0	-1,902123	-2,159129	-3,306152
12	6	0	-0.981647	-1,980751	-0.768275
13	6	0	0.10235	-2,409607	-0.251493
14	6	0	1,087459	-3,853325	1,612425
15	1	0	1,299133	-4,892754	1,877633
16	1	0	2,028826	-3,29668	1,616197
17	1	0	0.417886	-3,421685	2,356624
18	79	0	1,547134	-0.865949	-0.156952
19	15	0	3,23408	0.768544	-0.002077
20	6	0	4,752619	0.290078	-0.907482
21	6	0	5,145589	-1,05848	-0.90573
22	6	0	5,542335	1,238737	-1,575035
23	6	0	6,316464	-1,44912	-1,553611
24	1	0	4,535277	-1,80173	-0.399854
25	6	0	6,711472	0.84059	-2,22577
26	1	0	5,245598	2,282447	-1,593225
27	6	0	7,099781	-0.500121	-2,214924
28	1	0	6,612958	-2,493477	-1,548176
29	1	0	7,316388	1,579296	-2,742587
30	1	0	8,008111	-0.806339	-2,724713
31	6	0	3,732099	1,073266	1,73371
32	6	0	2,761181	0.983112	2,745039

33	6	0	5,052263	1,409297	2,07146
34	6	0	3,105098	1,236458	4,072414
35	1	0	1,739817	0.708865	2,494884
36	6	0	5,391015	1,657497	3,402482
37	1	0	5,815283	1,469758	1,302158
38	6	0	4,420081	1,572785	4,402137
39	1	0	2,3497	1,161659	4,848605
40	1	0	6,41507	1,912616	3,657194
41	1	0	4,688768	1,761795	5,436962
42	6	0	2,71495	2,383988	-0.696973
43	6	0	2,062091	2,399545	-1,94147
44	6	0	2,965715	3,593645	-0.03392
45	6	0	1,678199	3,60936	-2,516838
46	1	0	1,859947	1,466575	-2,461159
47	6	0	2,572037	4,802464	-0.612675
48	1	0	3,465378	3,595785	0.9291
49	6	0	1,931517	4,812481	-1,852123
50	1	0	1,180384	3,613607	-3,481824
51	1	0	2,76983	5,735113	-0.093189
52	1	0	1,62931	5,754329	-2,299926
53	7	0	4,341463	-0.683671	-0.706447
54	16	0	5,573544	-0.38258	0.554005
55	6	0	5,763996	1,382498	0.508694
56	8	0	6,779084	-1,017384	0.035702
57	8	0	4,918765	-0.786064	1,792104
58	6	0	6,799964	1,937514	-0.248239
59	6	0	4,894063	2,18213	1,257613
60	6	0	6,950558	3,321935	-0.260581
61	1	0	7,483962	1,296589	-0.793315
62	6	0	5,065392	3,562117	1,228767
63	1	0	4,117183	1,729841	1,864511
64	6	0	6,091699	4,154202	0.473467
65	1	0	7,755693	3,762439	-0.841346
66	1	0	4,399075	4,192103	1,810941
67	6	0	6,289372	5,648723	0.483846
68	1	0	6,760245	5,998098	-0.438649
69	1	0	5,341089	6,178078	0.610463
70	1	0	6,940835	5,943113	1,315642
71	1	0	2,974636	-1,280056	0.715306
72	6	0	0.46528	-3,807425	0.213836
73	1	0	1,188453	-4,200007	-0.507576
74	8	0	0.627734	-4,75578	0.09382
75	6	0	1,747749	-4,595901	0.846843
76	8	0	1,957593	-3,637632	1,559941
77	6	0	2,674552	-5,773285	0.671228

78	1	0	2,792829	-6,020287	-0.386727
79	1	0	-2,242768	-6,650837	1,163341
80	1	0	-3,639365	-5,546134	1,123643

TS1

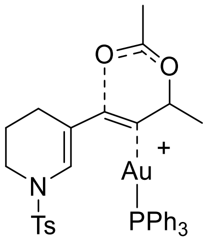

G at M06/def2tzvpp (IEFPCM, dichloromethane) = −2.623,141034 Hartree
Freq = −246,5
Correction at B3LYP/6-31G(d,p) = 0.552977
Cartesian coordinates of the computed structure

Center Number	Atomic Number	Atomic type	Coordinates (Angstroms)		
			X	Y	Z
1	6	0	1,807357	2,725228	-0.508788
2	6	0	2,269384	1,841638	0.429591
3	1	0	2,446945	2,529038	-2,561611
4	6	0	2,690146	3,129651	-1,674024
5	6	0	4,435584	1,542804	-0.742278
6	6	0	4,16586	2,946282	-1,290824
7	1	0	5,446078	1,477878	-0.332151
8	1	0	4,814649	3,109309	-2,156421
9	1	0	4,333087	0.789033	-1,532366
10	1	0	4,440658	3,686491	-0.531653
11	1	0	2,500808	4,172945	-1,938192
12	6	0	0.495521	3,224995	-0.33358
13	6	0	0.771935	2,979158	-0.128137
14	6	0	1,85365	4,247351	1,784762
15	1	0	2,577937	5,03107	2,021662
16	1	0	2,181089	3,314533	2,251792
17	1	0	0.879381	4,513479	2,204124
18	79	0	1,399446	0.982975	-0.135665
19	15	0	2,121205	-1,257053	-0.014864
20	6	0	3,942974	-1,426491	-0.12257
21	6	0	4,742861	-0.515522	0.588006
22	6	0	4,55428	-2,441123	-0.873281
23	6	0	6,131701	-0.625685	0.554367
24	1	0	4,278908	0.277672	1,168236
25	6	0	5,946747	-2,542631	-0.908088
26	1	0	3,950407	-3,147976	-1,432652
27	6	0	6,735308	-1,638591	-0.195541
28	1	0	6,742411	0.081207	1,107636
29	1	0	6,412783	-3,328952	-1,493949

30	1	0	7,817516	-1,719662	-0.226452
31	6	0	1,618939	-2,042809	1,561829
32	6	0	0.408837	-1,65014	2,157438
33	6	0	2,396218	-3,039912	2,171948
34	6	0	0.027482	-2,256206	3,335215
35	1	0	0.194173	-0.863789	1,713353
36	6	0	1,958587	-3,638732	3,354606
37	1	0	-3,341785	-3,342323	1,732852
38	6	0	-0.748557	-3,250829	3,934647
39	1	0	0.966606	-1,939713	3,777754
40	1	0	-2,566651	-4,405994	3,824164
41	1	0	-0.415256	-3,71808	4,856365
42	6	0	-1,427499	-2,295575	-1,359321
43	6	0	-1,292159	-1,737914	-2,641686
44	6	0	-1,054783	-3,631569	-1,146362
45	6	0	-0.802618	-2,50859	-3,695763
46	1	0	-1,570515	-0.701782	-2,814791
47	6	0	-0.562296	-4,398148	-2,205204
48	1	0	-1,149298	-4,074162	-0.15999
49	6	0	-0.436918	-3,83927	-3,478849
50	1	0	-0.705087	-2,07001	-4,684171
51	1	0	-0.285013	-5,434184	-2,034177
52	1	0	-0.056604	-4,438673	-4,300512
53	7	0	3,486241	1,229317	0.346223
54	6	0	3,941472	0.043937	1,528869
55	6	0	4,194754	-1,42609	0.552458
56	8	0	5,228934	0.466326	2,071175
57	8	0	2,753839	-0.114642	2,373111
58	6	0	5,500889	-1,843054	0.285147
59	6	0	3,092123	-2,144584	0.07938
60	6	0	5,697124	-2,993203	-0.476883
61	1	0	6,340803	-1,28225	0.67975
62	6	0	3,311456	-3,287112	-0.683431
63	1	0	2,082422	-1,827823	0.315921
64	6	0	4,61265	-3,730777	-0.973042
65	1	0	6,710413	-3,32426	-0.685519
66	1	0	2,457755	-3,845163	-1,057624
67	6	0	4,836925	-4,990802	-1,771019
68	1	0	4,036441	-5,152626	-2,49826
69	1	0	4,860954	-5,866437	-1,110884
70	1	0	5,788773	-4,961844	-2,307789
71	1	0	1,676264	1,567335	1,293562
72	6	0	-1,758076	4,048265	0.274157
73	1	0	-2,741492	3,829529	-0.141872
74	8	0	-1,475032	5,371805	-0.338507
75	6	0	-0.250104	5,765213	-0.565902
76	8	0	0.757801	5,05178	-0.384168

77	6	0	-0.129727	7,157726	-1,105419
78	1	0	-0.347247	7,137363	-2,179435
79	1	0	-0.855813	7,817333	-0.627145
80	1	0	0.886214	7,524138	-0.958777

II

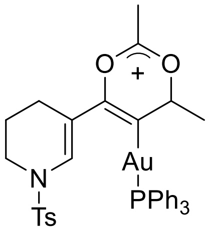

G at M06/def2tzvpp (IEFPCM, dichloromethane) = −2.623,150129 Hartree
Correction at B3LYP/6-31G(d,p) = 0.553648
Cartesian coordinates of the computed structure

Center Number	Atomic Number	Atomic type	Coordinates (Angstroms)		
			X	Y	Z
1	6	0	0.930161	3,20463	-0.530256
2	6	0	1,526453	2,308508	0.297877
3	1	0	1,59105	3,430688	-2,573002
4	6	0	1,740271	3,916399	-1,597552
5	6	0	3,708567	2,503089	-0.869998
6	6	0	3,229768	3,911641	-1,227002
7	1	0	4,73259	2,533721	-0.491987
8	1	0	3,834925	4,289546	-2,05654
9	1	0	3,687152	1,849037	-1,750251
10	1	0	3,398369	4,571945	-0.369316
11	1	0	1,396181	4,948015	-1,715342
12	6	0	0.48844	3,470079	-0.355803
13	6	0	1,561358	2,712997	-0.101323
14	6	0	3,228497	3,327962	1,726328
15	1	0	4,160912	3,868881	1,908701
16	1	0	3,35889	2,289019	2,039675
17	1	0	2,427274	3,764755	2,329231
18	79	0	1,541889	0.63173	-0.068756
19	15	0	1,628022	-1,729301	-0.030327
20	6	0	3,336501	-2,356216	-0.266075
21	6	0	4,378045	-1,739089	0.447572
22	6	0	3,629174	-3,431278	-1,117202
23	6	0	5,687832	-2,196865	0.317252
24	1	0	4,162431	-0.90333	1,107964
25	6	0	4,944851	-3,881468	-1,249571
26	1	0	2,837446	-3,916482	-1,678203
27	6	0	5,97326	-3,267773	-0.533985
28	1	0	6,485571	-1,716103	0.875308
29	1	0	5,162939	-4,71283	-1,912957
30	1	0	6,994738	-3,620078	-0.639913
31	6	0	1,036825	-2,443173	1,5491

32	6	0	0.028885	-1,814895	2,213958
33	6	0	1,60134	-3,608276	2,092379
34	6	0	0.531999	-2,352658	3,398332
35	1	0	0.472382	-0.905396	1,821128
36	6	0	1,097492	-4,137413	3,281651
37	1	0	2,433918	-4,0967	1,595964
38	6	0	0.031602	-3,512915	3,933565
39	1	0	1,35732	-1,854564	3,897097
40	1	0	1,541014	-5,036154	3,699457
41	1	0	0.354344	-3,927247	4,860123
42	6	0	-0.619494	-2,498559	-1,357459
43	6	0	-0.545617	-1,860813	-2,607215
44	6	0	0.050071	-3,716833	-1,166042
45	6	0	0.176183	-2,439015	-3,651007
46	1	0	-1,05398	-0.912915	-2,762503
47	6	0	0.773647	-4,290626	-2,21414
48	1	0	0.006898	-4,217546	-0.204172
49	6	0	0.836332	-3,654638	-3,455979
50	1	0	0.224428	-1,94043	-4,614338
51	1	0	1,281176	-5,238357	-2,060113
52	1	0	1,396862	-4,104887	-4,269737
53	7	0	2,845908	1,919345	0.176553
54	16	0	3,579737	1,010941	1,438306
55	6	0	4,348283	-0.33374	0.551825
56	8	0	4,637796	1,824293	2,035982
57	8	0	2,459974	0.508745	2,242901
58	6	0	5,741679	-0.408547	0.516164
59	6	0	3,554294	-1,306312	-0.06404
60	6	0	6,342718	-1,470872	-0.157698
61	1	0	6,336352	0.349538	1,013471
62	6	0	4,174195	-2,355484	-0.734327
63	1	0	2,472167	-1,249339	-0.01683
64	6	0	5,574587	-2,457322	-0.791015
65	1	0	7,426704	-1,533691	-0.188941
66	1	0	3,560158	-3,108555	-1,220131
67	6	0	6,233315	-3,619069	-1,492429
68	1	0	5,655636	-3,941038	-2,363658
69	1	0	6,315679	-4,481139	-0.819251
70	1	0	7,243658	-3,366964	-1,824687
71	1	0	0.99051	1,842112	1,11495
72	6	0	-2,869528	3,351324	0.246763
73	1	0	-3,687307	2,955285	-0.357691
74	8	0	-2,920589	4,833743	-0.154132
75	6	0	-1,856281	5,477415	-0.44475
76	8	0	-0.690399	4,954863	-0.487549
77	6	0	-1,985691	6,9231	-0.783561
78	1	0	-2,22103	7,010913	-1,851015

79	1	0	-2,80228	7,371255	-0.216058
80	1	0	-1,044102	7,438527	-0.592219

TS2

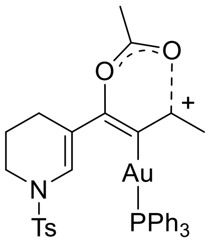

G at M06/def2tzvpp (IEFPCM, dichloromethane) = −2.623,137366 Hartree
Correction at B3LYP/6-31G(d,p) = 0.552471
Freq = −241,83
Cartesian coordinates of the computed structure

Center Number	Atomic Number	Atomic type	Coordinates (Angstroms)		
			X	Y	Z
1	6	0	1,281252	3,147436	-0.620155
2	6	0	1,688583	2,079283	0.126571
3	1	0	2,198726	3,646533	-2,505667
4	6	0	2,29293	3,926223	-1,446622
5	6	0	3,979298	2,162261	-0.804946
6	6	0	3,722395	3,661228	-0.959705
7	1	0	4,964645	1,981283	-0.372215
8	1	0	4,448939	4,07771	-1,663668
9	1	0	3,932351	1,652732	-1,774783
10	1	0	3,8855	4,147519	0.008381
11	1	0	2,078567	4,995977	-1,395807
12	6	0	0.108528	3,556791	-0.589002
13	6	0	1,256233	2,853656	-0.390405
14	6	0	3,717068	3,148884	0.329742
15	1	0	4,386932	4,001199	0.463969
16	1	0	4,255225	2,394223	-0.256682
17	1	0	3,458373	2,716749	1,298529
18	79	0	1,408319	0.778543	-0.143747
19	15	0	1,794476	-1,547341	0.023455
20	6	0	1,519095	-2,251937	1,690585
21	6	0	0.440265	-1,777511	2,454389
22	6	0	2,341188	-3,26744	2,205198
23	6	0	0.184377	-2,320553	3,713383
24	1	0	0.206866	-0.992591	2,076616
25	6	0	2,081348	-3,801443	3,468128
26	1	0	3,183608	-3,635805	1,628677
27	6	0	1,004546	-3,329873	4,222083
28	1	0	0.653249	-1,946574	4,293785
29	1	0	2,722832	-4,583631	3,862608
30	1	0	0.808264	-3,745694	5,205787
31	6	0	0.759928	-2,52399	-1,136133

32	6	0	0.4135	-1,961889	-2,376495
33	6	0	0.342295	-3,828321	-0.83031
34	6	0	0.325234	-2,699526	-3,301568
35	1	0	0.724775	-0.948934	-2,617773
36	6	0	0.399842	-4,561671	-1,758485
37	1	0	0.594783	-4,271096	0.127777
38	6	0	0.730651	-4,001016	-2,99449
39	1	0	0.583553	-2,259103	-4,259825
40	1	0	0.713362	-5,572701	-1,516278
41	1	0	1,300726	-4,576939	-3,7175
42	6	0	-3,537498	-1,937124	-0.404381
43	6	0	-4,557314	-1,210547	0.234916
44	6	0	-3,879528	-2,926579	-1,336172
45	6	0	-5,895159	-1,477035	-0.049462
46	1	0	-4,3045	-0.44333	0.962098
47	6	0	-5,222502	-3,183795	-1,623568
48	1	0	-3,105483	-3,496773	-1,838507
49	6	0	-6,229637	-2,462717	-0.982458
50	1	0	-6,676133	-0.915488	0.454323
51	1	0	-5,477944	-3,950791	-2,348314
52	1	0	-7,272049	-2,666166	-1,207616
53	7	0	2,963522	1,566405	0.085977
54	16	0	3,500056	0.556497	1,385149
55	6	0	4,259058	-0.807356	0.524974
56	8	0	4,540396	1,277188	2,117091
57	8	0	2,268385	0.116782	2,047356
58	6	0	5,641795	-0.976026	0.619682
59	6	0	3,464487	-1,705415	-0.194857
60	6	0	6,233772	-2,057749	-0.030429
61	1	0	6,234348	-0.275933	1,197963
62	6	0	4,076818	-2,775966	-0.837655
63	1	0	2,388913	-1,575398	-0.24926
64	6	0	5,466604	-2,971686	-0.765803
65	1	0	7,309159	-2,194015	0.038653
66	1	0	3,463945	-3,473164	-1,401522
67	6	0	6,111727	-4,156441	-1,440489
68	1	0	6,065697	-5,041388	-0.794097
69	1	0	7,16541	-3,96811	-1,661573
70	1	0	5,604451	-4,410354	-2,375657
71	1	0	1,022813	1,584903	0.823513
72	6	0	-2,498369	3,558588	-0.433357
73	1	0	-2,715674	4,108109	-1,351884
74	8	0	-2,124419	5,275606	0.413467
75	6	0	-1,046383	5,716005	0.001113
76	8	0	-0.173861	4,996822	-0.704877
77	6	0	-0.60089	7,122738	0.247948
78	1	0	-1,434479	7,725332	0.605854

79	1	0	0.197823	7,117291	0.997478
80	1	0	-0.185964	7,542736	-0.672305

III

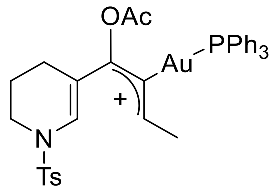

G at M06/def2tzvpp (IEFPCM, dichloromethane) = −2.623,169205 Hartree
Correction at B3LYP/6-31G(d,p) = 0.549677
Cartesian coordinates of the computed structure

Center Number	Atomic Number	Atomic type	Coordinates (Angstroms)		
			X	Y	Z
1	6	0	-2,455777	1,837824	-0.220266
2	6	0	-3,187612	0.685977	-0.518933
3	1	0	-3,385901	3,71745	0.256832
4	6	0	-3,002341	3,194823	-0.629543
5	6	0	-5,130738	1,988282	-1,278714
6	6	0	-4,110089	3,05466	-1,681293
7	1	0	-5,848385	1,810513	-2,080992
8	1	0	-4,630157	4,006936	-1,817942
9	1	0	-5,678142	2,277245	-0.374078
10	1	0	-3,677879	2,777045	-2,648863
11	1	0	-2,190974	3,813425	-1,020681
12	6	0	-1,207231	1,701388	0.413418
13	6	0	-0.651026	0.486914	0.951478
14	6	0	-1,045891	-1,610349	2,347445
15	1	0	-0.939091	-1,531284	3,437217
16	1	0	-1,838091	-2,347867	2,166235
17	1	0	-0.107702	-1,989239	1,935394
18	79	0	1,321889	0.018871	0.428193
19	15	0	3,556428	-0.498803	-0.147605
20	6	0	4,465516	0.988904	-0.723837
21	6	0	3,840802	1,83103	-1,660767
22	6	0	5,750599	1,306742	-0.263176
23	6	0	4,498387	2,965383	-2,134693
24	1	0	2,844026	1,593821	-2,024023
25	6	0	6,401783	2,449069	-0.73598
26	1	0	6,243506	0.668738	0.462861
27	6	0	5,779735	3,277105	-1,670522
28	1	0	4,01218	3,605449	-2,864952
29	1	0	7,396605	2,688086	-0.372614
30	1	0	6,289611	4,163013	-2,03666
31	6	0	3,697466	-1,726987	-1,499687
32	6	0	2,745221	-2,756438	-1,577502
33	6	0	4,738891	-1,677865	-2,439226

34	6	0	2,841164	-3,727851	-2,573179
35	1	0	1,928031	-2,794824	-0.86236
36	6	0	4,827348	-2,650638	-3,436329
37	1	0	5,474712	-0.880973	-2,398688
38	6	0	3,881648	-3,67545	-3,503502
39	1	0	2,100156	-4,519521	-2,627022
40	1	0	5,634257	-2,604964	-4,161354
41	1	0	3,95189	-4,428565	-4,282433
42	6	0	4,502163	-1,169128	1,271399
43	6	0	4,200496	-0.702309	2,561978
44	6	0	5,522194	-2,117783	1,101336
45	6	0	4,918491	-1,16926	3,662552
46	1	0	3,402131	0.020989	2,705633
47	6	0	6,233333	-2,585286	2,207594
48	1	0	5,75691	-2,496791	0.111897
49	6	0	5,93446	-2,111417	3,486435
50	1	0	4,678702	-0.803726	4,65636
51	1	0	7,018525	-3,322202	2,068904
52	1	0	6,487983	-2,479877	4,344781
53	7	0	-4,427524	0.714601	-1,017821
54	16	0	-5,19	-0.812949	-1,529191
55	6	0	-6,555914	-0.968814	-0.406232
56	8	0	-5,688952	-0.544096	-2,872222
57	8	0	-4,177838	-1,823837	-1,238097
58	6	0	-7,825347	-0.55165	-0.816726
59	6	0	-6,342558	-1,529416	0.857923
60	6	0	-8,892132	-0.689229	0.068106
61	1	0	-7,974422	-0.150638	-1,813079
62	6	0	-7,423642	-1,65565	1,723188
63	1	0	-5,356193	-1,87594	1,146372
64	6	0	-8,712138	-1,239682	1,346103
65	1	0	-9,883012	-0.371935	-0.243206
66	1	0	-7,269751	-2,092823	2,70551
67	6	0	-9,880148	-1,416435	2,282433
68	1	0	-10.304283	-2,422695	2,180013
69	1	0	-10.678515	-0.701472	2,068382
70	1	0	-9,578521	-1,295838	3,326524
71	1	0	-2,755988	-0.300601	-0.397972
72	6	0	-1,416585	-0.28374	1,764689
73	1	0	-2,378314	0.112813	2,103335
74	8	0	0.351199	2,723183	2,51498
75	6	0	0.425261	3,194443	1,417326
76	8	0	-0.466571	2,84529	0.379659
77	6	0	1,383833	4,235986	0.920772
78	1	0	1,919191	4,663727	1,767703
79	1	0	2,09883	3,76903	0.23419
80	1	0	0.854653	5,016417	0.368475

TS3

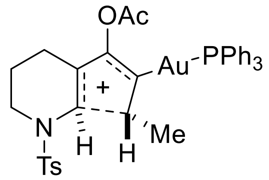

G at M06/def2tzvpp (IEFPCM, dichloromethane) = −2.623,14631190 Hartree
Correction at B3LYP/6-31G(d,p) = 0.549999
Freq = −307,590
Cartesian coordinates of the computed structure

Center Number	Atomic Number	Atomic type	Coordinates (Angstroms) X	Y	Z
1	6	0	-1,740408	-0.242734	0.835852
2	6	0	-2,345209	2,179557	0.360663
3	6	0	-2,98678	0.981923	-0.126107
4	6	0	-0.604153	0.610877	0.693269
5	1	0	-2,833795	3,854276	1,575926
6	6	0	-3,115783	3,450374	0.595578
7	6	0	-5,01975	1,825142	0.931365
8	6	0	-4,64159	3,245924	0.49712
9	1	0	-6,096575	1,657733	0.888794
10	1	0	-5,157073	3,98196	1,120958
11	6	0	-1,757527	-1,674391	0.369826
12	1	0	-2,78459	-2,037117	0.278776
13	1	0	-1,254786	-1,797742	-0.592101
14	1	0	-1,251629	-2,30905	1,107523
15	79	0	1,340369	0.017734	0.352846
16	15	0	3,591345	-0.620468	-0.048365
17	6	0	4,500283	0.662832	-0.990145
18	6	0	3,828063	1,341766	-2,02089
19	6	0	5,84261	0.970011	-0.722481
20	6	0	4,493919	2,30467	-2,778101
21	1	0	2,785833	1,116723	-2,231855
22	6	0	6,502178	1,939776	-1,480233
23	1	0	6,371637	0.459399	0.075596
24	6	0	5,831239	2,605466	-2,507383
25	1	0	3,967781	2,822249	-3,574425
26	1	0	7,540462	2,17382	-1,265805
27	1	0	6,347567	3,35922	-3,094017
28	6	0	3,722171	-2,168602	-1,020618
29	6	0	2,862811	-3,234204	-0.703935
30	6	0	4,659447	-2,321202	-2,052655
31	6	0	2,948728	-4,436699	-1,403322
32	1	0	2,1294	-3,123264	0.090396
33	6	0	4,736371	-3,526319	-2,754023
34	1	0	5,324159	-1,504052	-2,312957
35	6	0	3,884518	-4,583205	-2,430625
36	1	0	2,282578	-5,256246	-1,151453

37	1	0	5,462312	-3,636199	-3,553794
38	1	0	3,946052	-5,517866	-2,979598
39	6	0	4,53146	-0.902511	1,498522
40	6	0	4,24631	-0.104275	2,618722
41	6	0	5,537198	-1,877429	1,583384
42	6	0	4,965238	-0.272284	3,801539
43	1	0	3,460454	0.644648	2,566191
44	6	0	6,25056	-2,043737	2,771621
45	1	0	5,759548	-2,509026	0.729241
46	6	0	5,967285	-1,242336	3,879099
47	1	0	4,738147	0.34775	4,663386
48	1	0	7,025571	-2,801732	2,831091
49	1	0	6,522219	-1,376907	4,802543
50	8	0	-0.117279	2,961792	0.998821
51	6	0	0.112959	3,972865	0.069636
52	6	0	1,147643	4,927352	0.591564
53	1	0	2,114629	4,417847	0.652216
54	1	0	1,224435	5,779556	-0.082337
55	1	0	0.888306	5,258763	1,600388
56	8	0	-0.453585	4,014519	-0.990842
57	1	0	-4,699457	1,625508	1,962493
58	1	0	-4,987551	3,387754	-0.530454
59	1	0	-2,781043	4,187673	-0.144281
60	1	0	-2,325993	-0.06871	1,738449
61	7	0	-4,35557	0.835291	0.05908
62	16	0	-5,275534	0.197187	-1,287766
63	6	0	-6,442895	-0.891808	-0.501007
64	8	0	-6,008398	1,313851	-1,884818
65	8	0	-4,291931	-0.580337	-2,044869
66	6	0	-7,746083	-0.44157	-0.274469
67	6	0	-6,054116	-2,191703	-0.162505
68	6	0	-8,66019	-1,304638	0.3269
69	1	0	-8,040042	0.555065	-0.584866
70	6	0	-6,983922	-3,036649	0.433859
71	1	0	-5,052513	-2,540364	-0.388276
72	6	0	-8,297739	-2,60941	0.691033
73	1	0	-9,675006	-0.961372	0.50558
74	1	0	-6,690968	-4,049337	0.696037
75	6	0	-9,303313	-3,548537	1,307835
76	1	0	-10.109374	-3,003626	1,805554
77	1	0	-8,834345	-4,213897	2,038203
78	1	0	-9,760908	-4,18153	0.537922
79	1	0	-2,637451	0.547383	-1,058712
80	6	0	-1,006578	1,958396	0.68656

IV

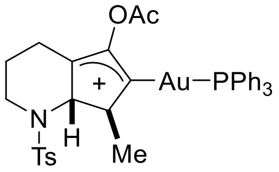

G at M06/def2tzvpp (IEFPCM, dichloromethane) = −2.623,16206390 Hartree
Correction at B3LYP/6-31G(d,p) = 0.550864
Cartesian coordinates of the computed structure

Center Number	Atomic Number	Atomic type	Coordinates (Angstroms)		
			X	Y	Z
1	6	0	-1,808052	-0.113391	-0.191058
2	6	0	-2,36487	2,19461	0.204655
3	6	0	-2,943486	0.960897	-0.400624
4	6	0	-0.585259	0.703877	0.099626
5	1	0	-2,70592	3,844031	1,466235
6	6	0	-3,186474	3,362713	0.605955
7	6	0	-4,769983	1,476158	1,20796
8	6	0	-4,657385	2,974679	0.898356
9	1	0	-5,802221	1,192212	1,422983
10	1	0	-5,026991	3,560467	1,745274
11	6	0	-1,651674	-1,155067	-1,303713
12	1	0	-2,604032	-1,651263	-1,498054
13	1	0	-1,329669	-0.685241	-2,238057
14	1	0	-0.905738	-1,903602	-1,023496
15	79	0	1,354804	0.068698	0.071538
16	15	0	3,626614	-0.644333	0.02294
17	6	0	4,707427	0.59864	-0.779481
18	6	0	4,231866	1,267119	-1,920671
19	6	0	5,993464	0.884142	-0.297593
20	6	0	5,036712	2,199135	-2,574199
21	1	0	3,235061	1,057162	-2,2999
22	6	0	6,792349	1,823151	-0.953146
23	1	0	6,370522	0.380495	0.586525
24	6	0	6,317134	2,479019	-2,089895
25	1	0	4,663113	2,709015	-3,456927
26	1	0	7,785985	2,040659	-0.573562
27	1	0	6,941317	3,208813	-2,596434
28	6	0	3,852627	-2,209774	-0.90089
29	6	0	2,91571	-3,240634	-0.716582
30	6	0	4,937635	-2,409049	-1,767048
31	6	0	3,069202	-4,455252	-1,382691
32	1	0	2,069079	-3,094203	-0.050909
33	6	0	5,082459	-3,626104	-2,436106
34	1	0	5,664566	-1,618947	-1,924062
35	6	0	4,15181	-4,648247	-2,244791
36	1	0	2,342068	-5,2477	-1,234288
37	1	0	5,922854	-3,772479	-3,107618

38	1	0	4,266623	-5,59216	-2,768744
39	6	0	4,30025	-0.927573	1,702297
40	6	0	3,888984	-0.084847	2,748501
41	6	0	5,22733	-1,948565	1,960997
42	6	0	4,406997	-0.255799	4,031572
43	1	0	3,163598	0.702249	2,55995
44	6	0	5,739024	-2,117105	3,248747
45	1	0	5,545025	-2,613299	1,164168
46	6	0	5,331564	-1,272378	4,282797
47	1	0	4,08402	0.398733	4,835325
48	1	0	6,454248	-2,910516	3,442644
49	1	0	5,729612	-1,408764	5,283647
50	8	0	-0.133016	2,967155	0.871553
51	6	0	0.001018	4,138477	0.131946
52	6	0	1,033105	5,033845	0.752434
53	1	0	2,01917	4,565841	0.668815
54	1	0	1,037041	5,992075	0.234775
55	1	0	0.826674	5,17496	1,816619
56	8	0	-0.64295	4,339877	-0.865016
57	1	0	-4,179197	1,215115	2,096644
58	1	0	-5,294087	3,199539	0.040579
59	1	0	-3,124825	4,100986	-0.207354
60	1	0	-2,067508	-0.624412	0.748935
61	7	0	-4,283263	0.629807	0.095997
62	16	0	-5,448268	0.299968	-1,138005
63	6	0	-6,508618	-0.88789	-0.334647
64	8	0	-6,24275	1,500251	-1,426946
65	8	0	-4,667042	-0.341792	-2,202205
66	6	0	-7,840232	-0.546712	-0.092627
67	6	0	-6,014757	-2,156453	-0.013373
68	6	0	-8,681791	-1,491536	0.493987
69	1	0	-8,205199	0.436258	-0.368731
70	6	0	-6,87061	-3,082956	0.570827
71	1	0	-4,98176	-2,413594	-0.220539
72	6	0	-8,215821	-2,768895	0.832393
73	1	0	-9,718781	-1,231575	0.685796
74	1	0	-6,494083	-4,069995	0.824549
75	6	0	-9,139281	-3,7973	1,435838
76	1	0	-10.009441	-3,330498	1,90403
77	1	0	-8,626513	-4,402722	2,189002
78	1	0	-9,508809	-4,483713	0.664422
79	1	0	-2,99069	1,119008	-1,489824
80	6	0	-1,000484	2,00954	0.390074

V

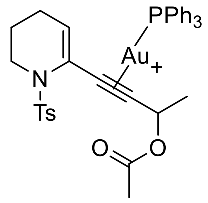

G at M06/def2tzvpp (IEFPCM, dichloromethane) = −2,623.14860701 Hartree
Correction at B3LYP/6-31G(d,p) = 0.548700
Cartesian coordinates of the computed structure

Center Number	Atomic Number	Atomic type	Coordinates (Angstroms)		
			X	Y	Z
1	6	0	1.963813	-2.215946	2.627371
2	6	0	2.174233	-1.660462	1.405659
3	1	0	2.161671	-1.419796	4.574333
4	6	0	2.800833	-1.921107	3.831269
5	6	0	3.663488	-0.024782	2.433272
6	6	0	4.029837	-1.065999	3.496107
7	1	0	4.517772	0.603062	2.17367
8	1	0	4.405378	-0.567187	4.394643
9	1	0	2.869015	0.640774	2.791744
10	1	0	4.836549	-1.692049	3.105312
11	1	0	3.094697	-2.868275	4.3019
12	1	0	1.112136	-2.880339	2.741703
13	6	0	1.318196	-2.024965	0.32628
14	6	0	0.548714	-2.389954	-0.5786
15	6	0	0.271257	-3.275243	-1.756666
16	1	0	-0.09031	-2.655488	-2.581173
17	6	0	1.506591	-4.074763	-2.157669
18	1	0	1.279674	-4.66579	-3.04856
19	1	0	2.336969	-3.398135	-2.366111
20	1	0	1.801856	-4.749073	-1.350498
21	8	0	-0.761145	-4.236275	-1.429043
22	6	0	-2.04856	-3.82769	-1.490988
23	6	0	-2.994353	-4.962454	-1.201327
24	1	0	-4.013453	-4.657631	-1.436545
25	1	0	-2.718465	-5.846976	-1.780214
26	1	0	-2.928558	-5.23099	-0.141771
27	8	0	-2.372598	-2.680305	-1.738955
28	79	0	-0.810844	-0.698927	-0.079986
29	15	0	-2.404637	1.00997	0.176654
30	6	0	-3.994666	0.366571	0.808778
31	6	0	-4.500882	-0.827264	0.266169
32	6	0	-4.721334	1.051563	1.793902
33	6	0	-5.729017	-1.319026	0.70616
34	1	0	-3.941965	-1.367479	-0.493576
35	6	0	-5.947039	0.54626	2.231293
36	1	0	-4.335119	1.97116	2.220848
37	6	0	-6.451308	-0.636424	1.688621

38	1	0	-6.121422	-2.2391	0.283761
39	1	0	-6.50491	1.077737	2.996071
40	1	0	-7.404439	-1.027401	2.031526
41	6	0	-2.752053	1.826546	-1.421412
42	6	0	-1.694909	2.052457	-2.317905
43	6	0	-4.047096	2.250792	-1.755393
44	6	0	-1.929976	2.702874	-3.527871
45	1	0	-0.69151	1.716204	-2.07057
46	6	0	-4.275984	2.898655	-2.970468
47	1	0	-4.874186	2.071576	-1.076303
48	6	0	-3.220885	3.125279	-3.855558
49	1	0	-1.10935	2.871678	-4.218271
50	1	0	-5.280668	3.221146	-3.225612
51	1	0	-3.404283	3.624761	-4.801891
52	6	0	-1.845523	2.3095	1.337967
53	6	0	-1.14502	1.936345	2.496688
54	6	0	-2.13195	3.663501	1.108232
55	6	0	-0.748647	2.904576	3.417622
56	1	0	-0.911078	0.89045	2.676843
57	6	0	-1.725484	4.629013	2.031175
58	1	0	-2.665845	3.96579	0.21335
59	6	0	-1.036767	4.251968	3.184876
60	1	0	-0.212414	2.609444	4.314443
61	1	0	-1.947929	5.675357	1.846133
62	1	0	-0.722167	5.005647	3.900227
63	7	0	3.155812	-0.648163	1.187293
64	16	0	4.386662	-1.01834	0.012713
65	6	0	4.791802	0.606467	-0.600659
66	8	0	5.569133	-1.566091	0.684646
67	8	0	3.6956	-1.792779	-1.022677
68	6	0	6.08589	1.0954	-0.414729
69	6	0	3.834166	1.336915	-1.31039
70	6	0	6.41591	2.343602	-0.941069
71	1	0	6.814986	0.502737	0.126562
72	6	0	4.184233	2.580239	-1.825619
73	1	0	2.836455	0.936648	-1.455195
74	6	0	5.47752	3.102759	-1.653616
75	1	0	7.420703	2.730771	-0.798214
76	1	0	3.445921	3.156967	-2.375841
77	6	0	5.854433	4.436153	-2.249345
78	1	0	6.644727	4.924032	-1.672742
79	1	0	4.996058	5.112013	-2.297164
80	1	0	6.227759	4.309466	-3.272876

TS4

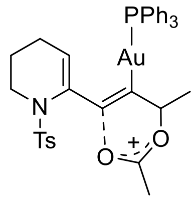

G at M06/def2tzvpp (IEFPCM, dichloromethane) = −2,623.12599759 Hartree
Correction at B3LYP/6-31G(d,p) = 0.55129300
Freq = −163.874
Cartesian coordinates of the computed structure

Center Number	Atomic Number	Atomic type	Coordinates (Angstroms)		
			X	Y	Z
1	6	0	2.51774	-2.03965	2.464879
2	6	0	2.482297	-1.36413	1.286957
3	1	0	2.550977	-1.360226	4.469849
4	6	0	3.274388	-1.584006	3.67108
5	6	0	3.409068	0.58239	2.42911
6	6	0	4.156871	-0.36658	3.370487
7	1	0	3.992098	1.478913	2.211927
8	1	0	4.422058	0.154686	4.295218
9	1	0	2.465265	0.91173	2.880858
10	1	0	5.087548	-0.675581	2.886204
11	1	0	3.870948	-2.422921	4.052694
12	1	0	1.942486	-2.957982	2.533058
13	6	0	1.76441	-1.923881	0.190209
14	6	0	0.807061	-2.093391	-0.639885
15	6	0	0.81694	-2.970438	-1.873522
16	1	0	-0.195422	-3.312082	-2.089982
17	6	0	1.399992	-2.236509	-3.0809
18	1	0	1.417476	-2.907637	-3.943633
19	1	0	0.769819	-1.375789	-3.323037
20	1	0	2.40644	-1.873421	-2.864591
21	8	0	1.525181	-4.236342	-1.690494
22	6	0	2.659657	-4.318859	-0.999804
23	6	0	3.299999	-5.673556	-1.101223
24	1	0	3.710913	-5.799624	-2.108172
25	1	0	4.10303	-5.757088	-0.369996
26	1	0	2.553565	-6.457252	-0.949117
27	8	0	3.142302	-3.395507	-0.34612
28	79	0	-0.878012	-0.895374	-0.227022
29	15	0	-2.867924	0.348143	0.113787
30	6	0	-4.031459	-0.517978	1.230076
31	6	0	-4.150786	-1.914068	1.129857
32	6	0	-4.818549	0.179078	2.159072
33	6	0	-5.053108	-2.600364	1.940737
34	1	0	-3.538568	-2.462533	0.418927
35	6	0	-5.716655	-0.514944	2.971905
36	1	0	-4.729776	1.256487	2.252093

37	6	0	-5.835708	-1.901363	2.863294
38	1	0	-5.140644	-3.679216	1.857161
39	1	0	-6.321016	0.029459	3.690955
40	1	0	-6.533706	-2.437453	3.499021
41	6	0	-3.753931	0.627942	-1.463307
42	6	0	-3.005493	0.916457	-2.616655
43	6	0	-5.153799	0.577547	-1.541791
44	6	0	-3.650245	1.161771	-3.82776
45	1	0	-1.920199	0.946685	-2.566482
46	6	0	-5.793284	0.81899	-2.75919
47	1	0	-5.743263	0.345044	-0.660883
48	6	0	-5.04472	1.111651	-3.900246
49	1	0	-3.065563	1.384013	-4.715142
50	1	0	-6.876614	0.7747	-2.814309
51	1	0	-5.545758	1.295352	-4.845717
52	6	0	-2.560013	1.996188	0.848944
53	6	0	-1.566978	2.123186	1.833956
54	6	0	-3.309631	3.121187	0.473715
55	6	0	-1.337615	3.355823	2.443193
56	1	0	-0.975712	1.258354	2.12305
57	6	0	-3.071598	4.354072	1.083954
58	1	0	-4.073337	3.0384	-0.292656
59	6	0	-2.089112	4.472425	2.068335
60	1	0	-0.570338	3.446687	3.205971
61	1	0	-3.654451	5.220926	0.78806
62	1	0	-1.906477	5.433001	2.540328
63	7	0	3.055537	-0.061925	1.140893
64	16	0	4.23717	0.159485	-0.117142
65	6	0	4.073309	1.909659	-0.433711
66	8	0	5.582379	-0.090931	0.410425
67	8	0	3.73466	-0.597272	-1.265183
68	6	0	5.178814	2.738138	-0.236913
69	6	0	2.87176	2.41066	-0.943678
70	6	0	5.068823	4.093283	-0.547081
71	1	0	6.103762	2.322187	0.147134
72	6	0	2.7827	3.765818	-1.243689
73	1	0	2.024921	1.750406	-1.09869
74	6	0	3.876952	4.627451	-1.05518
75	1	0	5.925217	4.743931	-0.394802
76	1	0	1.851234	4.165148	-1.635597
77	6	0	3.777664	6.087672	-1.420875
78	1	0	4.479351	6.694821	-0.843035
79	1	0	2.768664	6.475409	-1.254081
80	1	0	4.012671	6.23698	-2.481774

VI

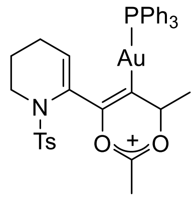

G at M06/def2tzvpp (IEFPCM, dichloromethane) = −2,623.13910150 Hartree
Correction at B3LYP/6-31G(d,p) = 0.555640
Cartesian coordinates of the computed structure

Center Number	Atomic Number	Atomic type	Coordinates (Angstroms)		
			X	Y	Z
1	6	0	2.154899	-2.057396	2.61387
2	6	0	2.2774	-1.489874	1.399796
3	1	0	1.530713	-1.252028	4.480364
4	6	0	2.464806	-1.368721	3.911717
5	6	0	2.488225	0.691084	2.490076
6	6	0	3.136846	-0.008863	3.68795
7	1	0	2.922016	1.677504	2.314086
8	1	0	3.045794	0.619831	4.579239
9	1	0	1.413881	0.83099	2.658942
10	1	0	4.20349	-0.135848	3.481576
11	1	0	3.100723	-2.019816	4.524449
12	1	0	1.773957	-3.074271	2.657732
13	6	0	1.984883	-2.256327	0.1695
14	6	0	0.980893	-2.205109	-0.698239
15	6	0	1.07613	-3.07109	-1.920696
16	1	0	0.669557	-4.072288	-1.734477
17	6	0	0.512024	-2.497058	-3.206695
18	1	0	0.734985	-3.153427	-4.051449
19	1	0	-0.572929	-2.412358	-3.107125
20	1	0	0.923888	-1.503433	-3.399673
21	8	0	2.539989	-3.363011	-2.256387
22	6	0	3.405621	-3.432742	-1.318502
23	6	0	4.798599	-3.81895	-1.649413
24	1	0	5.208305	-4.44034	-0.850165
25	1	0	4.847922	-4.326047	-2.612058
26	1	0	5.381331	-2.890158	-1.682676
27	8	0	3.128761	-3.157223	-0.095827
28	79	0	-0.693202	-1.034576	-0.348507
29	15	0	-2.679231	0.217606	0.077053
30	6	0	-4.020613	-0.235971	-1.087453
31	6	0	-3.704957	-0.358325	-2.451154
32	6	0	-5.339586	-0.450916	-0.663211
33	6	0	-4.696452	-0.681376	-3.375592
34	1	0	-2.684047	-0.196703	-2.787699
35	6	0	-6.327878	-0.781358	-1.593389
36	1	0	-5.596602	-0.366345	0.38753
37	6	0	-6.009236	-0.895982	-2.946923

38	1	0	-4.445095	-0.770595	-4.428132
39	1	0	-7.346307	-0.949777	-1.256909
40	1	0	-6.779943	-1.1545	-3.666599
41	6	0	-2.461262	2.029443	-0.085173
42	6	0	-1.221727	2.597386	0.249422
43	6	0	-3.508855	2.861033	-0.511318
44	6	0	-1.039737	3.978428	0.175801
45	1	0	-0.398413	1.9599	0.56016
46	6	0	-3.320177	4.241724	-0.586481
47	1	0	-4.466612	2.433	-0.789804
48	6	0	-2.08866	4.801436	-0.24036
49	1	0	-0.076834	4.40796	0.434462
50	1	0	-4.134832	4.878367	-0.917833
51	1	0	-1.945713	5.876203	-0.300058
52	6	0	-3.33074	-0.083233	1.763882
53	6	0	-3.250754	-1.383335	2.29118
54	6	0	-3.918131	0.935452	2.528737
55	6	0	-3.762762	-1.660206	3.557935
56	1	0	-2.789297	-2.177583	1.710531
57	6	0	-4.422889	0.653638	3.79962
58	1	0	-3.978778	1.946174	2.139025
59	6	0	-4.348142	-0.641583	4.314072
60	1	0	-3.70016	-2.668342	3.9562
61	1	0	-4.873569	1.448186	4.386476
62	1	0	-4.741347	-0.856873	5.302977
63	7	0	2.605824	-0.101538	1.239484
64	16	0	4.021021	0.261906	0.309708
65	6	0	3.668985	1.919621	-0.253073
66	8	0	5.213854	0.306407	1.163785
67	8	0	3.997669	-0.653819	-0.842651
68	6	0	4.553788	2.944736	0.085936
69	6	0	2.570258	2.154657	-1.084438
70	6	0	4.32442	4.226022	-0.413111
71	1	0	5.404567	2.734111	0.724516
72	6	0	2.358258	3.441957	-1.569878
73	1	0	1.894083	1.34693	-1.344074
74	6	0	3.231439	4.495191	-1.24893
75	1	0	5.009457	5.027589	-0.151637
76	1	0	1.503526	3.633506	-2.212502
77	6	0	3.017012	5.876279	-1.817038
78	1	0	3.359675	6.651995	-1.126678
79	1	0	1.963497	6.058827	-2.045891
80	1	0	3.579653	6.000485	-2.750246

TS5

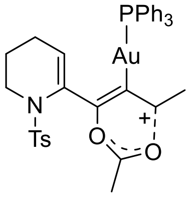

G at M06/def2tzvpp (IEFPCM, dichloromethane) = −2,623.13206718 Hartree
Correction at B3LYP/6-31G(d,p) = 0.55468200
Freq = −181.516
Cartesian coordinates of the computed structure

Center Number	Atomic Number	Atomic type	Coordinates (Angstroms)		
			X	Y	Z
1	6	0	-1.986001	-0.633572	-2.547467
2	6	0	-2.623907	-0.841278	-1.372873
3	1	0	-2.038005	1.385047	-3.306037
4	6	0	-2.471836	0.398582	-3.525548
5	6	0	-4.435874	0.792026	-1.998379
6	6	0	-4.006223	0.467722	-3.426755
7	1	0	-5.513956	0.667926	-1.872223
8	1	0	-4.415159	1.236358	-4.08967
9	1	0	-4.188878	1.838675	-1.779996
10	1	0	-4.430059	-0.491954	-3.740884
11	1	0	-2.156286	0.136678	-4.539582
12	1	0	-1.209674	-1.333734	-2.837217
13	6	0	-2.289943	-2.048041	-0.587048
14	6	0	-1.052599	-2.482321	-0.204574
15	6	0	-0.975199	-3.801165	0.30407
16	1	0	-1.504936	-4.580834	-0.245686
17	6	0	0.085267	-4.300443	1.218066
18	1	0	-0.277086	-5.149082	1.802833
19	1	0	0.919121	-4.663727	0.600753
20	1	0	0.460946	-3.514721	1.875831
21	8	0	-2.625135	-3.85289	1.567082
22	6	0	-3.568308	-3.336864	0.986722
23	6	0	-4.972396	-3.319477	1.489729
24	1	0	-5.65204	-3.595289	0.678598
25	1	0	-5.077609	-3.999662	2.333713
26	1	0	-5.216663	-2.297007	1.794349
27	8	0	-3.445664	-2.744225	-0.241257
28	79	0	0.624939	-1.242001	-0.161303
29	15	0	2.58767	0.101368	-0.019648
30	6	0	3.231794	0.157317	1.694425
31	6	0	2.321899	1.842082	-0.536211
32	6	0	3.950349	-0.528812	-1.070876
33	6	0	2.316972	0.147181	2.760677
34	6	0	4.607275	0.226028	1.965556
35	6	0	1.547718	2.090665	-1.682011
36	6	0	2.893062	2.920259	0.155938

37	6	0	4.118722	-1.917905	-1.192782
38	6	0	4.835678	0.333975	-1.734629
39	6	0	2.772221	0.217602	4.076809
40	1	0	1.250687	0.078661	2.562026
41	6	0	5.056867	0.291163	3.285344
42	1	0	5.326021	0.221243	1.152393
43	6	0	1.362433	3.395459	-2.136073
44	1	0	1.093396	1.262323	-2.218935
45	6	0	2.698611	4.226953	-0.299544
46	1	0	3.490156	2.743749	1.044474
47	6	0	5.163141	-2.434625	-1.957833
48	1	0	3.432235	-2.593331	-0.689501
49	6	0	5.876893	-0.189237	-2.50346
50	1	0	4.71267	1.409354	-1.658125
51	6	0	4.142208	0.288322	4.340157
52	1	0	2.058598	0.208819	4.894969
53	1	0	6.122404	0.339879	3.487788
54	6	0	1.937863	4.465721	-1.445615
55	1	0	0.769146	3.578178	-3.026897
56	1	0	3.148785	5.05533	0.239168
57	6	0	6.042386	-1.570597	-2.615149
58	1	0	5.286803	-3.509603	-2.046299
59	1	0	6.556366	0.484468	-3.016489
60	1	0	4.496111	0.33514	5.365522
61	1	0	1.79546	5.481549	-1.801921
62	1	0	6.8517	-1.973635	-3.216233
63	7	0	-3.761784	-0.087687	-1.003295
64	16	0	-3.97248	0.416989	0.621167
65	6	0	-3.207813	2.024945	0.761487
66	8	0	-5.41485	0.573031	0.800464
67	8	0	-3.201959	-0.527209	1.442022
68	6	0	-4.013288	3.165631	0.790605
69	6	0	-1.814557	2.118445	0.841484
70	6	0	-3.40681	4.416087	0.901061
71	1	0	-5.092348	3.067508	0.746755
72	6	0	-1.229861	3.376578	0.946787
73	1	0	-1.20159	1.222747	0.834283
74	6	0	-2.01297	4.542517	0.981159
75	1	0	-4.028341	5.306539	0.930542
76	1	0	-0.148315	3.457242	1.006757
77	6	0	-1.365294	5.896606	1.131616
78	1	0	-1.990063	6.690028	0.713064
79	1	0	-0.389837	5.927227	0.637922
80	1	0	-1.202804	6.13264	2.190357

VII

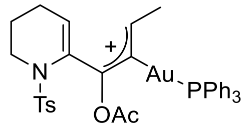

G at M06/def2tzvpp (IEFPCM, dichloromethane) = −2,623.14740142 Hartree
Correction at B3LYP/6-31G(d,p) = 0.54929500
Cartesian coordinates of the computed structure

Center Number	Atomic Number	Atomic type	Coordinates (Angstroms)		
			X	Y	Z
1	6	0	0.790453	-2.491382	-1.584703
2	6	0	2.573222	-3.104228	0.889737
3	6	0	2.779218	-1.824041	0.473884
4	6	0	1.650166	-1.001951	0.053026
5	6	0	0.545407	-1.496722	-0.669376
6	1	0	3.349247	-4.166446	2.521536
7	6	0	3.642996	-3.963947	1.480163
8	6	0	4.949045	-1.81576	1.595543
9	6	0	5.04286	-3.334954	1.414707
10	1	0	5.928637	-1.341231	1.514997
11	1	0	5.683819	-3.76242	2.191795
12	6	0	-0.232214	-3.26688	-2.334019
13	1	0	0.014219	-4.336065	-2.311352
14	1	0	-1.241332	-3.108804	-1.948234
15	1	0	-0.210859	-2.979622	-3.394781
16	79	0	-1.337213	-0.633609	-0.35555
17	15	0	-3.456607	0.349249	0.007226
18	6	0	-3.417024	1.558778	1.385982
19	6	0	-2.721265	1.214726	2.557907
20	6	0	-4.067631	2.798646	1.309909
21	6	0	-2.688999	2.093827	3.639263
22	1	0	-2.209432	0.258195	2.625401
23	6	0	-4.025133	3.678659	2.393765
24	1	0	-4.604445	3.079667	0.409878
25	6	0	-3.339628	3.32799	3.557521
26	1	0	-2.154601	1.817026	4.543087
27	1	0	-4.530338	4.637341	2.326368
28	1	0	-3.310534	4.01409	4.398575
29	6	0	-4.750241	-0.87432	0.443033
30	6	0	-4.771534	-2.102184	-0.239168
31	6	0	-5.725287	-0.609313	1.416061
32	6	0	-5.75904	-3.04493	0.041681
33	1	0	-4.01659	-2.319033	-0.990048
34	6	0	-6.708823	-1.559807	1.697214
35	1	0	-5.716744	0.331558	1.956574
36	6	0	-6.727891	-2.775178	1.011558
37	1	0	-5.769114	-3.990897	-0.491104
38	1	0	-7.458661	-1.348598	2.453456

39	1	0	-7.493062	-3.512514	1.234292
40	6	0	-4.076173	1.24797	-1.46417
41	6	0	-3.150797	1.871039	-2.3177
42	6	0	-5.44784	1.342173	-1.746631
43	6	0	-3.592942	2.587045	-3.429721
44	1	0	-2.086139	1.790377	-2.115122
45	6	0	-5.883992	2.056805	-2.863271
46	1	0	-6.173173	0.85431	-1.103324
47	6	0	-4.959323	2.679998	-3.703582
48	1	0	-2.871159	3.06372	-4.085922
49	1	0	-6.946297	2.122572	-3.077567
50	1	0	-5.302363	3.231482	-4.573654
51	8	0	1.635818	0.297232	0.4072
52	6	0	2.005844	0.711951	1.71565
53	6	0	2.177625	2.198384	1.741205
54	1	0	2.270403	2.529128	2.774907
55	1	0	3.082885	2.464642	1.186352
56	1	0	1.330498	2.68868	1.255036
57	8	0	2.090965	-0.062451	2.623506
58	1	0	4.53608	-1.554505	2.575722
59	1	0	5.512101	-3.543567	0.450415
60	1	0	3.626865	-4.946327	0.989083
61	1	0	1.825751	-2.684918	-1.871138
62	1	0	1.553971	-3.473097	0.923591
63	7	0	4.044772	-1.189863	0.598688
64	16	0	4.775003	-0.814102	-0.949783
65	6	0	5.569927	0.757487	-0.677507
66	8	0	5.812283	-1.807666	-1.239441
67	8	0	3.62828	-0.627842	-1.848351
68	6	0	6.913748	0.792018	-0.297702
69	6	0	4.850517	1.933505	-0.907761
70	6	0	7.529189	2.02832	-0.112489
71	1	0	7.471747	-0.12985	-0.176132
72	6	0	5.488052	3.158166	-0.724287
73	1	0	3.822869	1.88283	-1.251197
74	6	0	6.831556	3.227389	-0.320262
75	1	0	8.5734	2.061583	0.184789
76	1	0	4.940105	4.077264	-0.913107
77	6	0	7.521236	4.558688	-0.158049
78	1	0	8.299797	4.515794	0.608314
79	1	0	6.814231	5.34742	0.112696
80	1	0	8.003244	4.859287	-1.096223

TS6

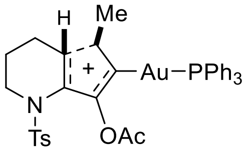

G at M06/def2tzvpp (IEFPCM, dichloromethane) = −2,623.13581392 Hartree
Correction at B3LYP/6-31G(d,p) = 0.55163500
Freq = −168.297
Cartesian coordinates of the computed structure

Center Number	Atomic Number	Atomic type	Coordinates (Angstroms)		
			X	Y	Z
1	6	0	-0.959943	-2.91378	0.738233
2	6	0	-2.644759	-3.167622	-0.831506
3	6	0	-2.952211	-1.839254	-0.502705
4	6	0	-1.816046	-0.983731	-0.245284
5	6	0	-0.691003	-1.616268	0.232157
6	1	0	-3.707633	-4.625056	-1.930504
7	6	0	-3.692914	-4.244704	-0.900011
8	6	0	-5.286453	-2.31023	-0.981039
9	6	0	-5.091155	-3.748435	-0.495291
10	1	0	-6.271306	-1.934522	-0.711156
11	1	0	-5.859435	-4.399051	-0.924166
12	6	0	0.024691	-4.037793	0.742865
13	1	0	-0.483913	-5.006801	0.73371
14	1	0	0.726782	-3.980451	-0.092081
15	1	0	0.600844	-3.9982	1.678204
16	79	0	1.195806	-0.765213	0.147567
17	15	0	3.327376	0.303803	0.052768
18	6	0	3.276304	1.761296	-1.059217
19	6	0	2.606556	1.64003	-2.28903
20	6	0	3.887626	2.977766	-0.724382
21	6	0	2.5617	2.716829	-3.173364
22	1	0	2.127025	0.701889	-2.556526
23	6	0	3.832928	4.055588	-1.611154
24	1	0	4.402083	3.087603	0.224572
25	6	0	3.173702	3.926858	-2.834188
26	1	0	2.050181	2.611898	-4.125479
27	1	0	4.307713	4.994718	-1.343893
28	1	0	3.135105	4.766333	-3.521747
29	6	0	4.653772	-0.785288	-0.586481
30	6	0	4.681394	-2.125888	-0.168448
31	6	0	5.644523	-0.313446	-1.460095
32	6	0	5.691997	-2.977996	-0.610519
33	1	0	3.913009	-2.500422	0.502704
34	6	0	6.650941	-1.173273	-1.904279
35	1	0	5.629227	0.7176	-1.798041
36	6	0	6.676645	-2.502761	-1.4805
37	1	0	5.708176	-4.012588	-0.281623

38	1	0	7.413057	-0.80261	-2.582919
39	1	0	7.459604	-3.168932	-1.829756
40	6	0	3.886239	0.904079	1.689203
41	6	0	2.923864	1.332291	2.618114
42	6	0	5.247112	0.960655	2.028107
43	6	0	3.31911	1.821128	3.862761
44	1	0	1.867454	1.278855	2.368853
45	6	0	5.636224	1.447194	3.276887
46	1	0	6.000074	0.619766	1.324704
47	6	0	4.674953	1.878218	4.193238
48	1	0	2.569243	2.148406	4.576403
49	1	0	6.690332	1.484871	3.534107
50	1	0	4.981314	2.251993	5.165494
51	8	0	-1.846318	0.362984	-0.500729
52	6	0	-2.183217	0.764693	-1.799961
53	6	0	-2.004655	2.247249	-1.94152
54	1	0	-2.34544	2.55596	-2.929013
55	1	0	-2.563738	2.77252	-1.16345
56	1	0	-0.946054	2.498301	-1.818495
57	8	0	-2.526144	-0.015984	-2.646159
58	1	0	-5.16814	-2.229919	-2.066921
59	1	0	-5.223196	-3.766969	0.590896
60	1	0	-3.387326	-5.100267	-0.283196
61	1	0	-1.768859	-3.001138	1.461694
62	1	0	-1.754181	-3.335027	-1.424057
63	7	0	-4.261185	-1.417839	-0.379716
64	16	0	-4.726112	-0.60134	1.127734
65	6	0	-5.052639	1.094596	0.689454
66	8	0	-5.989687	-1.232514	1.505626
67	8	0	-3.543766	-0.654361	1.988749
68	6	0	-6.215768	1.411271	-0.020489
69	6	0	-4.188558	2.089041	1.149411
70	6	0	-6.494041	2.746219	-0.292391
71	1	0	-6.900841	0.633316	-0.338941
72	6	0	-4.495286	3.42254	0.876678
73	1	0	-3.305931	1.81976	1.717482
74	6	0	-5.644601	3.773216	0.153655
75	1	0	-7.393669	2.998576	-0.846404
76	1	0	-3.836338	4.20404	1.244628
77	6	0	-5.986409	5.217757	-0.112055
78	1	0	-6.341544	5.361628	-1.136995
79	1	0	-5.125954	5.871868	0.048888
80	1	0	-6.787399	5.553925	0.556978

VIII

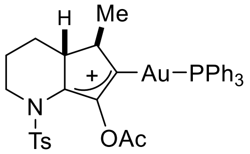

G at M06/def2tzvpp (IEFPCM, dichloromethane) = −2,623.17527065 Hartree

Correction at B3LYP/6-31G(d,p) = 0.55562100

Cartesian coordinates of the computed structure

Center Number	Atomic Number	Atomic type	Coordinates (Angstroms)		
			X	Y	Z
1	6	0	-0.937958	-2.638129	-0.355821
2	6	0	-2.483056	-2.617489	-0.237001
3	6	0	-2.801828	-1.180966	0.077092
4	6	0	-1.593459	-0.438318	0.186751
5	6	0	-0.490242	-1.23253	-0.035566
6	1	0	-3.11594	-4.577976	0.420911
7	6	0	-3.105188	-3.561109	0.822777
8	6	0	-5.059432	-1.976223	0.319884
9	6	0	-4.528338	-3.097545	1.222795
10	1	0	-5.998995	-1.586876	0.707203
11	1	0	-5.240965	-3.926439	1.180563
12	6	0	-0.436329	-3.137894	-1.724392
13	1	0	-0.786828	-4.157645	-1.910895
14	1	0	-0.794775	-2.493365	-2.533117
15	1	0	0.655943	-3.140139	-1.756162
16	79	0	1.456945	-0.613283	0.028006
17	15	0	3.717363	0.178178	0.010147
18	6	0	3.799097	1.964476	-0.389421
19	6	0	2.80887	2.51876	-1.217768
20	6	0	4.831601	2.782116	0.096821
21	6	0	2.864156	3.868991	-1.565274
22	1	0	1.988227	1.907249	-1.581734
23	6	0	4.877294	4.132586	-0.251187
24	1	0	5.593303	2.369916	0.75103
25	6	0	3.896804	4.676329	-1.08377
26	1	0	2.096095	4.287777	-2.208494
27	1	0	5.677507	4.759185	0.130851
28	1	0	3.935018	5.727946	-1.351779
29	6	0	4.735489	-0.694245	-1.240417
30	6	0	4.61431	-2.090548	-1.339903
31	6	0	5.626025	-0.017398	-2.085476
32	6	0	5.382489	-2.798186	-2.26257
33	1	0	3.920529	-2.62184	-0.693375
34	6	0	6.388505	-0.731047	-3.01277
35	1	0	5.72217	1.0616	-2.02585
36	6	0	6.269467	-2.118394	-3.101603
37	1	0	5.285382	-3.877465	-2.331082
38	1	0	7.073818	-0.199819	-3.666228

39	1	0	6.862746	-2.669355	-3.825049
40	6	0	4.581513	-0.039914	1.611194
41	6	0	3.848418	0.118691	2.798408
42	6	0	5.951048	-0.337229	1.680037
43	6	0	4.479819	-0.005688	4.035171
44	1	0	2.78476	0.337087	2.753993
45	6	0	6.577046	-0.464812	2.92119
46	1	0	6.525869	-0.475204	0.769784
47	6	0	5.844345	-0.297988	4.097655
48	1	0	3.906049	0.118513	4.948523
49	1	0	7.636576	-0.697609	2.967115
50	1	0	6.334027	-0.400831	5.061284
51	8	0	-1.580316	0.866506	0.623863
52	6	0	-1.07483	1.851865	-0.215882
53	6	0	-1.198772	3.196863	0.438637
54	1	0	-0.521612	3.898755	-0.047365
55	1	0	-2.229097	3.54511	0.317606
56	1	0	-0.9894	3.132299	1.508219
57	8	0	-0.619639	1.611466	-1.304254
58	1	0	-5.248812	-2.332698	-0.699586
59	1	0	-4.528356	-2.730169	2.253463
60	1	0	-2.46001	-3.575496	1.706052
61	1	0	-0.516704	-3.300157	0.413609
62	1	0	-2.942575	-2.84759	-1.208588
63	7	0	-4.082758	-0.855917	0.253297
64	16	0	-4.650125	0.867395	0.469718
65	6	0	-6.377367	0.754408	0.072839
66	8	0	-4.487459	1.173076	1.884817
67	8	0	-3.973609	1.605984	-0.590737
68	6	0	-7.314777	0.747218	1.111076
69	6	0	-6.768464	0.762825	-1.270839
70	6	0	-8.668657	0.729001	0.78613
71	1	0	-6.985613	0.7706	2.144333
72	6	0	-8.126998	0.740036	-1.56832
73	1	0	-6.025817	0.80566	-2.060245
74	6	0	-9.096766	0.723892	-0.551054
75	1	0	-9.405501	0.727098	1.583901
76	1	0	-8.442918	0.747277	-2.607456
77	6	0	-10.564907	0.738631	-0.890896
78	1	0	-11.167924	0.307408	-0.087967
79	1	0	-10.768534	0.1865	-1.812399
80	1	0	-10.910815	1.767986	-1.045016

TS7

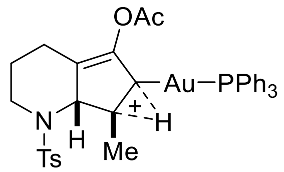

G at M06/def2tzvpp (IEFPCM, dichloromethane) = −2.623,13255003 Hartree
Freq = −778,150
Correction at B3LYP/6-31G(d,p) = 0.546915
Cartesian coordinates of the computed structure

Center Number	Atomic Number	Atomic type	Coordinates (Angstroms)		
			X	Y	Z
1	6	0	-1,811429	-0.074911	-0.226908
2	6	0	-2,34602	2,24149	0.11345
3	6	0	-2,922466	0.949951	-0.422476
4	6	0	-0.639782	0.612232	0.185296
5	1	0	-2,800794	4,003889	1,183649
6	6	0	-3,191279	3,458248	0.316914
7	6	0	-4,827485	1,629749	1,009478
8	6	0	-4,680047	3,077503	0.524038
9	1	0	-5,875176	1,374241	1,181932
10	1	0	-5,137221	3,751262	1,255264
11	6	0	-1,935524	-1,513959	-0.588434
12	1	0	-2,779354	-1,973794	-0.06607
13	1	0	-2,163826	-1,581581	-1,657277
14	1	0	-1,012474	-2,058051	-0.37847
15	79	0	1,348634	-0.019687	0.143346
16	15	0	3,624977	-0.634805	0.005333
17	6	0	4,625772	0.696903	-0.756337
18	6	0	4,080046	1,42226	-1,829338
19	6	0	5,921745	0.994634	-0.309103
20	6	0	4,825468	2,423304	-2,450582
21	1	0	3,074745	1,20406	-2,180252
22	6	0	6,660846	2,002132	-0.931975
23	1	0	6,352476	0.447948	0.523419
24	6	0	6,115927	2,714911	-2,001318
25	1	0	4,397161	2,977895	-3,279817
26	1	0	7,662242	2,229023	-0.579282
27	1	0	6,693607	3,498468	-2,482026
28	6	0	3,887316	-2,138166	-1,007877
29	6	0	3,026001	-3,233055	-0.823186
30	6	0	4,927668	-2,227849	-1,943847
31	6	0	3,209973	-4,402099	-1,559062
32	1	0	2,215748	-3,172605	-0.101202
33	6	0	5,102862	-3,399938	-2,682719
34	1	0	5,596554	-1,388078	-2,100481
35	6	0	4,247591	-4,485679	-2,491495
36	1	0	2,542078	-5,244887	-1,40944
37	1	0	5,908351	-3,461349	-3,40797

38	1	0	4,38597	-5,394384	-3,069209
39	6	0	4,358698	-0.979281	1,647128
40	6	0	3,939231	-0.21895	2,751438
41	6	0	5,343242	-1,964232	1,819553
42	0	0	4,505052	-0.435174	4,00718
43	0	0	3,170235	0.539305	2,629237
44	0	0	5,90278	-2,178574	3,080297
45	0	0	5,668559	-2,565739	0.97685
46	0	0	5,486396	-1,415506	4,172646
47	0	0	4,175456	0.155682	4,856277
48	0	0	6,66227	-2,943822	3,207537
49	0	0	5,921906	-1,587431	5,152184
50	0	0	-0.144498	2,903686	0.952397
51	0	0	0.142983	4,074218	0.252474
52	0	0	1,162528	4,882617	1,00413
53	0	0	2,10959	4,335454	1,045302
54	0	0	1,310209	5,835354	0.4975
55	0	0	0.833987	5,047122	2,033837
56	0	0	-0.370854	4,342756	-0.800436
57	0	0	-4,307497	1,479156	1,963522
58	0	0	-5,237919	3,186923	-0.408431
59	0	0	-3,07569	4,13519	-0.538195
60	0	0	-1,220879	-0.056003	1,060014
61	0	0	-4,274521	0.630574	0.055022
62	0	0	-5,354733	0.157681	-1,215225
63	0	0	-6,518881	-0.890844	-0.363963
64	0	0	-6,081736	1,320968	-1,735585
65	0	0	-4,507925	-0.644481	-2,109552
66	0	0	-7,797475	-0.400086	-0.091021
67	0	0	-6,158248	-2,198831	-0.026606
68	0	0	-8,715162	-1,230181	0.551212
69	0	0	-8,067877	0.605649	-0.393141
70	0	0	-7,089657	-3,011143	0.610721
71	0	0	-5,175108	-2,579279	-0.280098
72	0	0	-8,379962	-2,542542	0.911787
73	0	0	-9,710798	-0.853416	0.766821
74	0	0	-6,81725	-4,029694	0.872811
75	0	0	-9,38935	-3,446347	1,574112
76	0	0	-8,915819	-4,106774	2,306243
77	0	0	-9,881251	-4,085106	0.830567
78	0	0	-10.169521	-2,873368	2,081485
79	0	0	-2,919043	1,021154	-1,528728
80	0	0	-1,051514	2,021292	0.413577

IX

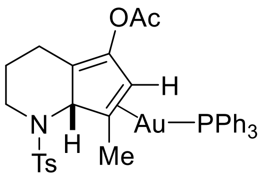

G at M06/def2tzvpp (IEFPCM, dichloromethane) = −2.623,18348520 Hartree
Correction at B3LYP/6-31G(d,p) = 0.552684
Cartesian coordinates of the computed structure

Center Number	Atomic Number	Atomic type	Coordinates (Angstroms)		
			X	Y	Z
1	6	0	1,174508	0.990547	-1,338876
2	6	0	1,503562	2,663838	0.370193
3	6	0	1,850878	1,230048	0.024862
4	6	0	0.381251	2,097883	-1,596841
5	1	0	2,116072	4,439996	1,343789
6	6	0	2,14607	3,361434	1,526671
7	6	0	4,127434	2,123709	0.475641
8	6	0	3,606574	2,856685	1,720239
9	1	0	5,150046	1,77108	0.628673
10	1	0	4,26795	3,701679	1,935852
11	6	0	1,617276	-0.072276	-2,295181
12	1	0	2,652356	0.148324	-2,586177
13	1	0	1,620861	-1,058074	-1,825642
14	1	0	0.998894	-0.089131	-3,195448
15	79	0	-1,117127	0.473072	-0.772285
16	15	0	-2,750543	-0.94427	0.113735
17	6	0	-2,249123	-1,44195	1,801675
18	6	0	-0.907505	-1,79758	2,031007
19	6	0	-3,16823	-1,478326	2,860355
20	6	0	-0.497487	-2,190346	3,303755
21	1	0	-0.178654	-1,770865	1,225024
22	6	0	-2,747868	-1,869735	4,133346
23	1	0	-4,203844	-1,199034	2,697586
24	6	0	-1,416877	-2,225064	4,356001
25	1	0	0.540888	-2,458964	3,470717
26	1	0	-3,463015	-1,89314	4,949826
27	1	0	-1,09411	-2,524806	5,34836
28	6	0	-2,92526	-2,474028	-0.874392
29	6	0	-3,002569	-2,371779	-2,273574
30	6	0	-3,011539	-3,733872	-0.264524
31	6	0	-3,17341	-3,516272	-3,04966
32	1	0	-2,930866	-1,399784	-2,754741
33	6	0	-3,178152	-4,877072	-1,049246
34	1	0	-2,944231	-3,825895	0.814481
35	6	0	-3,260076	-4,770101	-2,437985
36	1	0	-3,23379	-3,430912	-4,130251
37	1	0	-3,241788	-5,850167	-0.572226

38	1	0	-3,387325	-5,661523	-3,044471
39	6	0	-4,405165	-0.178687	0.206934
40	6	0	-4,520231	1,217555	0.303979
41	6	0	-5,561357	-0.977066	0.205241
42	6	0	-5,782153	1,803003	0.412499
43	1	0	-3,63698	1,848797	0.280226
44	6	0	-6,817733	-0.381758	0.313844
45	1	0	-5,483294	-2,055705	0.111314
46	6	0	-6,928823	1,006786	0.419313
47	1	0	-5,86685	2,882972	0.482337
48	1	0	-7,708622	-1,002102	0.310349
49	1	0	-7,909041	1,466979	0.498483
50	8	0	0.083396	4,377107	-0.570791
51	6	0	-1,284043	4,502274	-0.529272
52	6	0	-1,686277	5,947974	-0.591048
53	1	0	-1,310674	6,40036	-1,513564
54	1	0	-2,771683	6,026277	-0.551669
55	1	0	-1,239413	6,494949	0.244258
56	8	0	-2,025123	3,548585	-0.453652
57	1	0	4,152784	2,793466	-0.39151
58	1	0	3,663468	2,175808	2,571982
59	1	0	1,572243	3,190771	2,446955
60	7	0	3,300244	0.950928	0.079361
61	16	0	3,655565	-0.516083	0.913429
62	6	0	5,242305	-0.971234	0.238974
63	8	0	3,817735	-0.279256	2,354967
64	8	0	2,616886	-1,452835	0.452923
65	6	0	6,370551	-0.894201	1,057582
66	6	0	5,330953	-1,441604	-1,075105
67	6	0	7,606707	-1,278957	0.53958
68	1	0	6,27364	-0.549528	2,081095
69	6	0	6,57305	-1,821209	-1,571222
70	1	0	4,442637	-1,520618	-1,691447
71	6	0	7,729581	-1,74663	-0.775773
72	1	0	8,488389	-1,219232	1,171095
73	1	0	6,64967	-2,187583	-2,591168
74	6	0	9,064077	-2,190627	-1,320316
75	1	0	9,892892	-1,760121	-0.752891
76	1	0	9,181877	-1,906865	-2,370214
77	1	0	9,159374	-3,281881	-1,266606
78	1	0	1,32809	0.586757	0.75096
79	6	0	0.620096	3,104843	-0.544209
80	1	0	-0.096629	2,32391	-2,545665

X

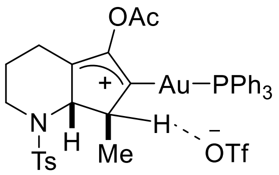

G at M06/def2tzvpp (IEFPCM, dichloromethane) = −3.584,85651732 Hartree

Correction at B3LYP/6-31G(d,p) = 0.566851

Cartesian coordinates of the computed structure

Center Number	Atomic Number	Atomic type	Coordinates (Angstroms)		
			X	Y	Z
1	6	0	-1,728146	0.834305	-0.96664
2	6	0	-2,257735	2,813764	0.285833
3	6	0	-2,876792	1,864729	-0.68988
4	6	0	-0.503673	1,464673	-0.414434
5	1	0	-2,496772	3,884333	2,08259
6	6	0	-3,052109	3,727896	1,150531
7	6	0	-4,521998	1,666306	1,198665
8	6	0	-4,477805	3,181622	1,421061
9	1	0	-5,524801	1,278606	1,382448
10	1	0	-4,772773	3,425686	2,446657
11	6	0	-1,613883	0.270271	-2,386524
12	1	0	-2,566972	-0.159537	-2,699569
13	1	0	-1,34684	1,051338	-3,106682
14	1	0	-0.847746	-0.508432	-2,41405
15	79	0	1,350141	0.622582	-0.471729
16	15	0	3,47276	-0.436292	-0.507442
17	6	0	4,708721	0.511574	-1,483411
18	6	0	4,276416	1,175118	-2,643548
19	6	0	6,060587	0.581767	-1,116622
20	6	0	5,184215	1,884546	-3,428951
21	1	0	3,227685	1,140429	-2,925975
22	6	0	6,964965	1,298487	-1,903003
23	1	0	6,406699	0.084009	-0.216427
24	6	0	6,529717	1,947897	-3,059214
25	1	0	4,838806	2,394142	-4,323549
26	1	0	8,009281	1,349602	-1,609048
27	1	0	7,235045	2,506571	-3,667449
28	6	0	3,423256	-2,11427	-1,242946
29	6	0	2,307305	-2,924413	-0.97009
30	6	0	4,46531	-2,605886	-2,044832
31	6	0	2,251064	-4,218257	-1,49051
32	1	0	1,480209	-2,558698	-0.36532
33	6	0	4,39524	-3,899591	-2,563407
34	1	0	5,324342	-1,981883	-2,270477
35	6	0	3,289876	-4,706499	-2,285483
36	1	0	1,384966	-4,836804	-1,275971
37	1	0	5,202513	-4,273604	-3,186525

38	1	0	3,236508	-5,711975	-2,693205
39	6	0	4,200249	-0.630828	1,165585
40	6	0	3,949736	0.359252	2,128845
41	6	0	5,000225	-1,732782	1,502277
42	6	0	4,50564	0.255705	3,403259
43	1	0	3,308596	1,201405	1,884189
44	6	0	5,550881	-1,833591	2,780835
45	1	0	5,182517	-2,515849	0.773216
46	6	0	5,306694	-0.84026	3,730591
47	1	0	4,300251	1,022339	4,144399
48	1	0	6,163775	-2,69312	3,035605
49	1	0	5,730234	-0.925467	4,727001
50	8	0	0.001997	3,308133	1,100406
51	6	0	0.118615	4,663731	0.883038
52	6	0	1,204349	5,236155	1,753205
53	1	0	2,173805	4,845528	1,427979
54	1	0	1,199961	6,322262	1,670025
55	1	0	1,060397	4,930746	2,792893
56	8	0	-0.56369	5,265264	0.090996
57	1	0	-3,829583	1,129389	1,85791
58	1	0	-5,206086	3,646298	0.750653
59	1	0	-3,084882	4,710945	0.660147
60	1	0	-1,914113	0.008183	-0.256363
61	7	0	-4,151469	1,343217	-0.192063
62	16	0	-5,39169	1,039704	-1,290615
63	6	0	-5,788141	-0.680491	-0.974965
64	8	0	-6,57959	1,834753	-0.947578
65	8	0	-4,788903	1,190058	-2,624392
66	6	0	-6,989418	-1,169721	-1,500428
67	6	0	-4,929151	-1,510098	-0.253271
68	6	0	-7,312581	-2,509692	-1,31082
69	1	0	-7,663828	-0.509135	-2,03521
70	6	0	-5,277024	-2,848699	-0.06605
71	1	0	-4,018683	-1,127775	0.194254
72	6	0	-6,462759	-3,370809	-0.59677
73	1	0	-8,245691	-2,892973	-1,715847
74	1	0	-4,605969	-3,479126	0.511539
75	6	0	-6,815916	-4,827769	-0.419208
76	1	0	-6,49362	-5,416256	-1,287384
77	1	0	-7,896101	-4,969367	-0.314575
78	1	0	-6,326632	-5,251633	0.461812
79	1	0	-3,056535	2,407574	-1,630872
80	6	0	-0.895391	2,596181	0.322163
81	16	0	-1,315667	-1,885908	1,61637
82	8	0	-0.452543	-1,895454	0.40259
83	8	0	-1,900809	-3,179083	1,999361
84	8	0	-2,247859	-0.720561	1,66172

85	6	0	-0.104832	-1,483843	2,970811
86	9	0	0.882713	-2,392005	3,012326
87	9	0	-0.701971	-1,447986	4,168485
88	9	0	0.463084	-0.272717	2,755051

TS8

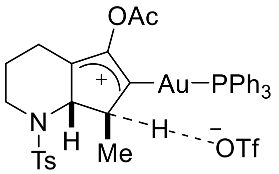

G at M06/def2tzvpp (IEFPCM, dichloromethane) = −3.584,83637599 Hartree
Freq = −1050.190
Correction at B3LYP/6-31G(d,p) = 0.564284
Cartesian coordinates of the computed structure

Center Number	Atomic Number	Atomic type	Coordinates (Angstroms)		
			X	Y	Z
1	6	0	-1,600193	0.810436	-0.846463
2	6	0	-2,148541	2,802955	0.384555
3	6	0	-2,738416	1,843995	-0.615578
4	6	0	-0.434825	1,357622	-0.285857
5	1	0	-2,489101	3,908803	2,152693
6	6	0	-2,969975	3,768995	1,177199
7	6	0	-4,50892	1,748753	1,157312
8	6	0	-4,427409	3,267372	1,347844
9	1	0	-5,531368	1,391918	1,296693
10	1	0	-4,801995	3,540678	2,340151
11	6	0	-1,61418	-0.090869	-2,067977
12	1	0	-2,550275	-0.649319	-2,138693
13	1	0	-1,519597	0.494659	-2,989469
14	1	0	-0.787093	-0.802994	-2,019312
15	79	0	1,459888	0.580755	-0.378348
16	15	0	3,616955	-0.394451	-0.48107
17	6	0	4,866225	0.715778	-1,245356
18	6	0	4,453509	1,561418	-2,288356
19	6	0	6,206425	0.740158	-0.831968
20	6	0	5,369774	2,405465	-2,91503
21	1	0	3,41323	1,562767	-2,602379
22	6	0	7,118941	1,59116	-1,45859
23	1	0	6,536751	0.102431	-0.018198
24	6	0	6,70344	2,421887	-2,500566
25	1	0	5,039325	3,055924	-3,719347
26	1	0	8,153826	1,605462	-1,129256
27	1	0	7,414923	3,084946	-2,983998
28	6	0	3,647047	-1,942591	-1,465133
29	6	0	2,54645	-2,810987	-1,36417
30	6	0	4,72639	-2,282781	-2,294778
31	6	0	2,539721	-4,009675	-2,078022
32	1	0	1,69358	-2,558133	-0.740318

33	6	0	4,708	-3,481772	-3,00941
34	1	0	5,57497	-1,61276	-2,390328
35	6	0	3,616993	-4,346087	-2,900515
36	1	0	1,684245	-4,67298	-1,994406
37	1	0	5,544856	-3,736704	-3,653205
38	1	0	3,603801	-5,276632	-3,460682
39	6	0	4,288599	-0.837393	1,1696
40	6	0	3,961678	-0.023915	2,266428
41	6	0	5,117051	-1,952806	1,363632
42	6	0	4,470072	-0.312902	3,532406
43	1	0	3,298674	0.825852	2,129521
44	6	0	5,619723	-2,240397	2,633716
45	1	0	5,360047	-2,601844	0.528255
46	6	0	5,299652	-1,420901	3,717526
47	1	0	4,207203	0.319201	4,375409
48	1	0	6,256074	-3,108924	2,775844
49	1	0	5,687396	-1,650691	4,705648
50	8	0	0.078047	3,125159	1,352054
51	6	0	0.315022	4,470782	1,20642
52	6	0	1,380794	4,912241	2,177061
53	1	0	2,336623	4,45458	1,902734
54	1	0	1,47224	5,997343	2,144704
55	1	0	1,136896	4,580094	3,18962
56	8	0	-0.251312	5,170206	0.403622
57	1	0	-3,878021	1,214212	1,877444
58	1	0	-5,088629	3,733342	0.61226
59	1	0	-2,947777	4,753336	0.692416
60	1	0	-1,78199	-0.073229	0.1946
61	7	0	-4,062666	1,351055	-0.194387
62	16	0	-5,26933	1,233213	-1,373017
63	6	0	-5,997529	-0.364848	-1,004051
64	8	0	-6,322809	2,239802	-1,163753
65	8	0	-4,566698	1,194451	-2,663659
66	6	0	-7,339744	-0.559505	-1,341373
67	6	0	-5,240411	-1,39768	-0.447499
68	6	0	-7,91689	-1,809123	-1,127614
69	1	0	-7,921491	0.261006	-1,74705
70	6	0	-5,839159	-2,637676	-0.231513
71	1	0	-4,213209	-1,230826	-0.146963
72	6	0	-7,178718	-2,866779	-0.57526
73	1	0	-8,96172	-1,962667	-1,384961
74	1	0	-5,247816	-3,428346	0.222508
75	6	0	-7,807572	-4,223617	-0.368697
76	1	0	-7,649225	-4,864744	-1,244935
77	1	0	-8,887569	-4,146495	-0.212346
78	1	0	-7,371875	-4,738501	0.492369
79	1	0	-2,842563	2,361625	-1,580387

80	6	0	-0.830113	2,503801	0.503942
81	16	0	-1,442176	-2,204453	1,239892
82	8	0	-0.417441	-2,396331	0.204642
83	8	0	-2,357969	-3,298503	1,552209
84	8	0	-2,197527	-0.865307	1,071118
85	6	0	-0.49008	-1,864878	2,802989
86	9	0	0.303073	-2,904894	3,079244
87	9	0	-1,320088	-1,662079	3,828584
88	9	0	0.277306	-0.771078	2,648684

XI

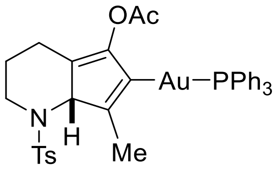

G at M06/def2tzvpp (IEFPCM, dichloromethane) = −2.622,74199393 Hartree
Correction at B3LYP/6-31G(d,p) = 0.540255
Cartesian coordinates of the computed structure

Center Number	Atomic Number	Atomic type	Coordinates (Angstroms)		
			X	Y	Z
1	6	0	-1,802673	0.093534	-0.57071
2	6	0	-2,420605	2,369896	-0.140234
3	6	0	-2,960197	1,07935	-0.711735
4	6	0	-0.693815	0.756648	-0.14484
5	1	0	-2,847171	4,306946	0.671507
6	6	0	-3,375618	3,448288	0.248599
7	6	0	-4,420666	1,326091	1,273245
8	6	0	-4,411211	2,87078	1,270148
9	1	0	-5,341383	0.939614	1,719778
10	1	0	-4,160797	3,2014	2,284913
11	6	0	-1,946766	-1,333108	-0.996584
12	1	0	-2,738415	-1,834906	-0.424483
13	1	0	-2,224825	-1,414197	-2,055944
14	1	0	-1,01214	-1,879639	-0.841942
15	79	0	1,234874	0.076621	-0.017304
16	15	0	3,495865	-0.634218	0.036508
17	6	0	4,674571	0.773021	0.151475
18	6	0	4,324308	1,981601	-0.474495
19	6	0	5,9019	0.677335	0.825292
20	6	0	5,201163	3,066696	-0.440178
21	1	0	3,36381	2,085491	-0.971851
22	6	0	6,771357	1,769019	0.860724
23	1	0	6,176675	-0.244363	1,328601
24	6	0	6,424382	2,962793	0.225248
25	1	0	4,919372	3,995157	-0.927934
26	1	0	7,717769	1,685947	1,387392
27	1	0	7,102032	3,811268	0.255381

28	6	0	3,999466	-1,565864	-1,467533
29	6	0	3,054285	-2,416023	-2,06456
30	6	0	5,279217	-1,460967	-2,032327
31	6	0	3,389879	-3,159727	-3,19541
32	1	0	2,05274	-2,483591	-1,64912
33	6	0	5,609291	-2,203274	-3,167941
34	1	0	6,014596	-0.793923	-1,593814
35	6	0	4,667823	-3,054427	-3,748755
36	1	0	2,649527	-3,811027	-3,650441
37	1	0	6,601393	-2,111207	-3,600452
38	1	0	4,925818	-3,62699	-4,634923
39	6	0	3,91515	-1,739134	1,446821
40	6	0	3,245914	-1,537317	2,665006
41	6	0	4,870975	-2,761811	1,352134
42	6	0	3,541626	-2,332567	3,771972
43	1	0	2,485369	-0.764787	2,738682
44	6	0	5,159796	-3,559368	2,461118
45	1	0	5,383088	-2,942275	0.412203
46	6	0	4,498892	-3,344447	3,67177
47	1	0	3,016153	-2,168611	4,708191
48	1	0	5,898796	-4,350917	2,376271
49	1	0	4,722905	-3,968767	4,531891
50	8	0	-0.32986	3,083623	0.80531
51	6	0	0.776199	3,584153	0.195468
52	6	0	1,542998	4,470245	1,149132
53	1	0	2,155685	3,841096	1,803994
54	1	0	2,199826	5,130486	0.582493
55	1	0	0.865341	5,049652	1,779392
56	8	0	1,103987	3,339116	-0.943974
57	1	0	-3,591214	0.945217	1,878658
58	1	0	-5,409521	3,246667	1,035777
59	1	0	-3,918319	3,805877	-0.635216
60	7	0	-4,24252	0.704598	-0.057384
61	16	0	-5,620334	0.636978	-1,037471
62	6	0	-6,533499	-0.719879	-0.296325
63	8	0	-6,454345	1,84218	-0.889985
64	8	0	-5,158955	0.220768	-2,368106
65	6	0	-7,712638	-0.4582	0.398224
66	6	0	-6,074543	-2,029932	-0.463775
67	6	0	-8,429941	-1,523278	0.947152
68	1	0	-8,063004	0.563932	0.490102
69	6	0	-6,802609	-3,07853	0.087425
70	1	0	-5,168528	-2,218617	-1,029122
71	6	0	-7,987697	-2,843419	0.805303
72	1	0	-9,351005	-1,32252	1,487922
73	1	0	-6,451286	-4,098815	-0.044357
74	6	0	-8,754766	-3,993272	1,412867

75	1	0	-9,745045	-3,679286	1,753259
76	1	0	-8,22268	-4,408687	2,27732
77	1	0	-8,88576	-4,808827	0.693936
78	1	0	-3,154219	1,194738	-1,786736
79	6	0	-1,125657	2,148321	0.136833

TS9

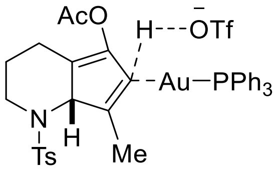

G at M06/def2tzvpp (IEFPCM, dichloromethane) = −3.584,83677004 Hartree
Freq = −1047,450
Correction at B3LYP/6-31G(d,p) = 0.563635
Cartesian coordinates of the computed structure

Center Number	Atomic Number	Atomic type	Coordinates (Angstroms)		
			X	Y	Z
1	6	0	-1,878508	-0.210804	-0.054605
2	6	0	-2,420261	0.767739	-2,170699
3	6	0	-2,906882	-0.262867	-1,177831
4	6	0	-0.830602	0.600961	-0.422984
5	1	0	-2,884637	2,040475	-3,824539
6	6	0	-3,35203	1,24948	-3,23253
7	6	0	-4,790997	1,337079	-1,091054
8	6	0	-4,681436	1,749022	-2,57384
9	1	0	-5,819516	1,432218	-0.732541
10	1	0	-4,733186	2,842629	-2,616025
11	6	0	-2,061083	-0.977942	1,209228
12	1	0	-2,989861	-0.662319	1,701383
13	1	0	-2,167393	-2,050743	1,005241
14	1	0	-1,223	-0.828141	1,893262
15	79	0	1,13562	-0.174608	-0.076859
16	15	0	3,204746	-1,235116	0.130922
17	6	0	4,608239	-0.222963	-0.483537
18	6	0	4,433181	0.507877	-1,671312
19	6	0	5,838699	-0.1709	0.186928
20	6	0	5,486627	1,265141	-2,18273
21	1	0	3,477539	0.495698	-2,189043
22	6	0	6,88514	0.59697	-0.328278
23	1	0	5,979888	-0.721379	1,11127
24	6	0	6,71196	1,312607	-1,513456
25	1	0	5,345388	1,826674	-3,101654
26	1	0	7,833097	0.636399	0.20035
27	1	0	7,526626	1,910945	-1,911171
28	6	0	3,27111	-2,793451	-0.84188
29	6	0	2,110581	-3,578168	-0.934671
30	6	0	4,444747	-3,226886	-1,476066

31	6	0	2,127976	-4,781331	-1,639109
32	1	0	1,192498	-3,239032	-0.463067
33	6	0	4,45642	-4,429933	-2,184604
34	1	0	5,345378	-2,623338	-1,42522
35	6	0	3,300847	-5,208472	-2,265663
36	1	0	1,223245	-5,378007	-1,707641
37	1	0	5,368705	-4,754909	-2,676328
38	1	0	3,311514	-6,141382	-2,821682
39	6	0	3,624494	-1,709844	1,853108
40	6	0	3,198165	-0.876173	2,900273
41	6	0	4,367529	-2,865185	2,143185
42	6	0	3,526615	-1,19477	4,218943
43	1	0	2,607604	0.011658	2,694879
44	6	0	4,68656	-3,177883	3,465157
45	1	0	4,68962	-3,523597	1,34266
46	6	0	4,268388	-2,342719	4,503424
47	1	0	3,191444	-0.544928	5,021661
48	1	0	5,258	-4,075733	3,682386
49	1	0	4,515216	-2,590429	5,531908
50	8	0	-0.55822	2,28054	-2,302032
51	6	0	0.688303	2,113621	-2,819117
52	6	0	1,27071	3,446262	-3,213638
53	1	0	0.536153	4,041905	-3,760625
54	1	0	1,533938	3,995339	-2,304557
55	1	0	2,16375	3,291127	-3,818448
56	8	0	1,238238	1,038678	-2,926018
57	1	0	-4,178455	1,996966	-0.467748
58	1	0	-5,535922	1,355703	-3,128014
59	1	0	-3,586704	0.427972	-3,920684
60	7	0	-4,318553	-0.035872	-0.792822
61	16	0	-5,402254	-1,298994	-1,143025
62	6	0	-6,592767	-1,141842	0.187086
63	8	0	-6,122489	-1,060898	-2,404177
64	8	0	-4,633492	-2,53755	-0.957824
65	6	0	-7,877183	-0.67932	-0.094066
66	6	0	-6,232793	-1,524849	1,48274
67	6	0	-8,805557	-0.581785	0.943933
68	1	0	-8,141392	-0.415793	-1,112211
69	6	0	-7,171312	-1,422485	2,503411
70	1	0	-5,238337	-1,909674	1,679615
71	6	0	-8,469164	-0.944736	2,253397
72	1	0	-9,808306	-0.222811	0.728808
73	1	0	-6,896999	-1,721933	3,511555
74	6	0	-9,467684	-0.820403	3,378472
75	1	0	-9,210322	0.015287	4,04031
76	1	0	-9,48714	-1,724894	3,995153
77	1	0	10.478192	-0.644936	3,000518

78	6	0	-1,231945	1,205506	-1,730203
79	1	0	-0.520955	1,567685	0.532742
80	8	0	-0.594412	2,60976	1,198234
81	16	0	0.548843	2,974752	2,16513
82	8	0	0.136708	3,992039	3,12472
83	8	0	1,279845	1,789737	2,634546
84	6	0	1,73832	3,831649	1,014971
85	9	0	2,819752	4,230465	1,687969
86	9	0	2,135836	2,987256	0.036864
87	9	0	1,167573	4,892306	0.433564
88	1	0	-2,824325	-1,272831	-1,607846

XII

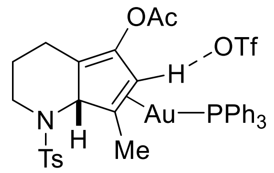

G at M06/def2tzvpp (IEFPCM, dichloromethane) = -3.584,875219510 Hartree
Correction at B3LYP/6-31G(d,p) = 0,568034
Cartesian Coordinates of the computed structure

Center Number	Atomic Number	Atomic type	Coordinates (Angstroms)		
			X	Y	Z
1	6	0	1,428855	-1,214612	0,29274
2	6	0	1,831947	-1,666035	-2,043408
3	6	0	2,240238	-0,73067	-0,924772
4	6	0	0,551215	-2,186489	-0,152974
5	1	0	2,307303	-2,574391	-3,900275
6	6	0	2,570495	-1,67179	-3,341235
7	6	0	4,448989	-1,67585	-1,583793
8	6	0	4,110548	-1,589498	-3,081114
9	1	0	5,518899	-1,525743	-1,4175
10	1	0	4,624916	-2,407112	-3,597692
11	6	0	1,797838	-0,887218	1,707732
12	1	0	2,743063	-1,392848	1,946394
13	1	0	1,960458	0,186333	1,837401
14	1	0	1,025086	-1,227943	2,399371
15	79	0	-0,816003	-0,253618	0,07065
16	15	0	-2,051628	1,704666	-0,142194
17	6	0	-3,652621	1,467584	-1,001
18	6	0	-3,73903	0,489024	-2,004402
19	6	0	-4,779527	2,24287	-0,687584
20	6	0	-4,936517	0,306652	-2,695928
21	1	0	-2,885983	-0,142613	-2,23303
22	6	0	-5,974642	2,050558	-1,381911
23	1	0	-4,729568	2,985101	0,1026
24	6	0	-6,053514	1,085499	-2,387728
25	1	0	-4,996895	-0,457298	-3,465148
26	1	0	-6,844675	2,649969	-1,130313

27	1	0	-6,987069	0,933447	-2,921812
28	6	0	-1,1278	2,966434	-1,107527
29	6	0	0,257881	3,081093	-0,906748
30	6	0	-1,761864	3,814953	-2,026319
31	6	0	0,99559	4,032661	-1,60894
32	1	0	0,769916	2,423431	-0,210734
33	6	0	-1,017895	4,765756	-2,729286
34	1	0	-2,82959	3,729574	-2,200987
35	6	0	0,357899	4,876181	-2,522261
36	1	0	2,067011	4,096644	-1,44678
37	1	0	-1,516349	5,415616	-3,4428
38	1	0	0,933359	5,612163	-3,076329
39	6	0	-2,421976	2,481759	1,475913
40	6	0	-2,604668	1,655633	2,59747
41	6	0	-2,541953	3,874919	1,607065
42	6	0	-2,922555	2,226721	3,831026
43	1	0	-2,485048	0,577985	2,520638
44	6	0	-2,857522	4,434577	2,845308
45	1	0	-2,379535	4,521116	0,749889
46	6	0	-3,050652	3,611005	3,956831
47	1	0	-3,059358	1,582152	4,693976
48	1	0	-2,946104	5,512878	2,941557
49	1	0	-3,292173	4,04949	4,92093
50	8	0	0,204862	-3,455226	-2,276178
51	6	0	-1,076282	-3,218102	-2,709443
52	6	0	-1,701398	-4,484179	-3,225907
53	1	0	-0,985826	-5,06037	-3,81656
54	1	0	-1,999462	-5,098246	-2,369284
55	1	0	-2,58443	-4,240853	-3,815966
56	8	0	-1,600634	-2,131247	-2,652899
57	1	0	4,204021	-2,667178	-1,187384
58	1	0	4,510713	-0,653035	-3,47461
59	1	0	2,260995	-0,820098	-3,960747
60	7	0	3,711093	-0,699415	-0,743474
61	16	0	4,395316	0,859561	-0,651025
62	6	0	5,790739	0,599515	0,440308
63	8	0	4,930476	1,293633	-1,951149
64	8	0	3,388055	1,687475	0,030846
65	6	0	7,082582	0,678833	-0,078175
66	6	0	5,572769	0,367757	1,802139
67	6	0	8,169199	0,503833	0,780258
68	1	0	7,226469	0,887496	-1,132468
69	6	0	6,668065	0,198082	2,641374
70	1	0	4,563654	0,333355	2,196233
71	6	0	7,981943	0,258074	2,145704
72	1	0	9,177658	0,566127	0,380798
73	1	0	6,503749	0,021184	3,700977

74	6	0	9,156887	0,054652	3,070758
75	1	0	9,219089	-0,98885	3,401703
76	1	0	9,065308	0,672044	3,970387
77	1	0	10,101379	0,305356	2,581306
78	1	0	1,887292	0,280208	-1,169646
79	6	0	0,837499	-2,440301	-1,578921
80	1	0	-0,062392	-2,844438	0,466582
81	8	0	-1,507193	-3,817828	1,549896
82	16	0	-2,174755	-2,805224	2,402987
83	8	0	-2,621347	-3,235493	3,731434
84	8	0	-1,491341	-1,465807	2,36949
85	6	0	-3,7498	-2,445102	1,476701
86	9	0	-4,420469	-1,427023	2,044198
87	9	0	-3,478121	-2,085885	0,19685
88	9	0	-4,552815	-3,513873	1,439014

XIII

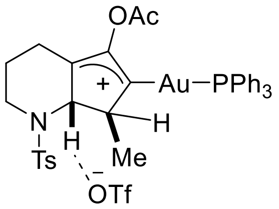

G at M06/def2tzvpp (IEFPCM, dichloromethane) = -3.584,84540662 Hartree
Correction at B3LYP/6-31G(d,p) = 0,570073
Cartesian Coordinates of the computed structure

Center Number	Atomic Number	Atomic type	Coordinates (Angstroms)		
			X	Y	Z
1	6	0	1,630721	0,837933	0,435263
2	6	0	2,22517	-1,207484	1,524917
3	6	0	2,72551	-0,283726	0,47596
4	6	0	0,433937	0,188078	1,079106
5	1	0	2,720522	-2,463672	3,143643
6	6	0	3,056129	-2,283447	2,117339
7	6	0	4,758299	-0,429767	1,919312
8	6	0	4,563176	-1,94736	2,03724
9	1	0	5,816775	-0,161862	1,909043
10	1	0	5,078187	-2,323001	2,927467
11	6	0	1,381944	1,484982	-0,929698
12	1	0	2,309352	1,895844	-1,334057
13	1	0	1,005828	0,738806	-1,632597
14	1	0	0,643151	2,286224	-0,838385
15	79	0	-1,495151	0,672535	0,634241
16	15	0	-3,688665	1,00139	-0,184209
17	6	0	-4,229217	-0,446822	-1,164183
18	6	0	-3,265456	-1,354513	-1,631591
19	6	0	-5,590497	-0,681455	-1,427531
20	6	0	-3,657522	-2,480561	-2,359446
21	1	0	-2,20918	-1,213885	-1,419033

22	6	0	-5,973975	-1,799053	-2,166688
23	1	0	-6,347657	-0,003548	-1,043834
24	6	0	-5,008274	-2,697845	-2,631232
25	1	0	-2,89248	-3,18335	-2,673174
26	1	0	-7,026946	-1,975903	-2,367406
27	1	0	-5,314012	-3,576062	-3,193045
28	6	0	-3,858964	2,490236	-1,247653
29	6	0	-3,263842	3,686582	-0,812433
30	6	0	-4,543247	2,471485	-2,470684
31	6	0	-3,368515	4,845871	-1,579077
32	1	0	-2,711574	3,705335	0,123396
33	6	0	-4,637303	3,63344	-3,240353
34	1	0	-4,993695	1,551365	-2,827367
35	6	0	-4,054988	4,820594	-2,79589
36	1	0	-2,905018	5,764945	-1,232527
37	1	0	-5,163883	3,606207	-4,189826
38	1	0	-4,12782	5,721529	-3,398109
39	6	0	-4,949641	1,191486	1,140579
40	6	0	-4,880967	0,316564	2,238606
41	6	0	-5,968069	2,15263	1,085816
42	6	0	-5,826925	0,402376	3,259032
43	1	0	-4,091987	-0,429249	2,290243
44	6	0	-6,90887	2,236251	2,115106
45	1	0	-6,027111	2,837215	0,245756
46	6	0	-6,840582	1,362466	3,200505
47	1	0	-5,768716	-0,278218	4,103452
48	1	0	-7,694061	2,985206	2,065697
49	1	0	-7,572596	1,430221	4,00013
50	8	0	0,280323	-1,730104	2,73687
51	6	0	-0,971631	-2,291505	2,590131
52	6	0	-1,025541	-3,601267	3,322144
53	1	0	-0,46269	-3,559094	4,257136
54	1	0	-2,062812	-3,882456	3,503382
55	1	0	-0,555455	-4,340654	2,664693
56	8	0	-1,880499	-1,79038	1,977013
57	1	0	4,306137	0,086868	2,776917
58	1	0	5,02391	-2,420834	1,167204
59	1	0	2,808791	-3,19697	1,557099
60	1	0	1,962874	1,607778	1,150421
61	7	0	4,12915	0,120257	0,696349
62	16	0	5,14801	0,123207	-0,678537
63	6	0	6,180456	1,551984	-0,345263
64	8	0	6,030378	-1,051874	-0,685215
65	8	0	4,274545	0,405893	-1,819857
66	6	0	7,554662	1,376086	-0,193194
67	6	0	5,608843	2,827215	-0,298254
68	6	0	8,362665	2,494106	0,021876

69	1	0	7,975925	0,378429	-0,248328
70	6	0	6,429066	3,928957	-0,083381
71	1	0	4,539066	2,951867	-0,426597
72	6	0	7,817461	3,781987	0,078854
73	1	0	9,434089	2,360359	0,143654
74	1	0	5,988562	4,921795	-0,043208
75	6	0	8,695454	4,992075	0,286738
76	1	0	9,691648	4,709619	0,636967
77	1	0	8,259465	5,680708	1,017699
78	1	0	8,819177	5,55078	-0,648838
79	1	0	2,615712	-0,819346	-0,479582
80	6	0	0,904661	-0,918452	1,815037
81	16	0	0,321854	-3,141768	-1,027175
82	8	0	-0,735758	-3,866985	-1,750832
83	8	0	0,532291	-3,539985	0,392362
84	8	0	0,347035	-1,666702	-1,248675
85	6	0	1,905136	-3,696956	-1,831719
86	9	0	2,06079	-5,023287	-1,730927
87	9	0	2,965816	-3,111637	-1,216069
88	9	0	1,941332	-3,358063	-3,123905

TS10

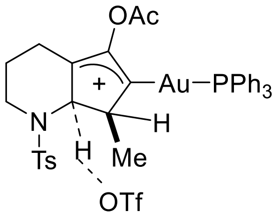

G at M06/def2tzvpp (IEFPCM, dichloromethane) = -3.584,83097091 Hartree
Freq = -1094,920
Correction at B3LYP/6-31G(d,p) = 0,564308
Cartesian Coordinates of the computed structure

Center Number	Atomic Number	Atomic type	Coordinates (Angstroms)		
			X	Y	Z
1	6	0	1,423052	1,08552	0,634689
2	6	0	2,089963	-0,831099	1,854517
3	6	0	2,557888	0,008679	0,811969
4	6	0	0,220075	0,42677	1,272367
5	1	0	2,740343	-1,636713	3,688682
6	6	0	2,933636	-1,79346	2,619006
7	6	0	4,709467	-0,130672	2,026029
8	6	0	4,422747	-1,614632	2,284909
9	1	0	5,774302	0,050188	1,867871
10	1	0	5,045902	-1,984217	3,106011
11	6	0	1,198593	1,711732	-0,743317
12	1	0	2,106201	2,197271	-1,104701
13	1	0	0,917045	0,953465	-1,475858
14	1	0	0,397221	2,454102	-0,6768

15	79	0	-1,720435	0,741791	0,696296
16	15	0	-3,872242	0,802384	-0,276164
17	6	0	-4,092909	-0,623082	-1,407809
18	6	0	-2,96227	-1,34197	-1,825783
19	6	0	-5,367586	-1,031634	-1,837713
20	6	0	-3,098901	-2,444221	-2,671602
21	1	0	-1,972555	-1,068385	-1,47499
22	6	0	-5,498965	-2,126263	-2,690971
23	1	0	-6,254857	-0,507014	-1,494675
24	6	0	-4,365776	-2,831537	-3,108417
25	1	0	-2,208608	-2,995974	-2,954718
26	1	0	-6,486821	-2,436593	-3,019654
27	1	0	-4,47516	-3,691693	-3,762916
28	6	0	-4,201743	2,331943	-1,240607
29	6	0	-3,805839	3,558628	-0,680571
30	6	0	-4,813084	2,321884	-2,502079
31	6	0	-4,036311	4,752688	-1,362043
32	1	0	-3,307549	3,574568	0,2851
33	6	0	-5,033022	3,519839	-3,185714
34	1	0	-5,107757	1,381988	-2,95642
35	6	0	-4,649871	4,734897	-2,61706
36	1	0	-3,725871	5,694436	-0,918993
37	1	0	-5,501121	3,499773	-4,165559
38	1	0	-4,820037	5,664315	-3,152695
39	6	0	-5,259172	0,66576	0,924799
40	6	0	-5,165198	-0,325356	1,917192
41	6	0	-6,388191	1,494408	0,882959
42	6	0	-6,193153	-0,485233	2,844773
43	1	0	-4,290956	-0,970279	1,957451
44	6	0	-7,411891	1,332858	1,820093
45	1	0	-6,470058	2,266183	0,124408
46	6	0	-7,316975	0,344396	2,799642
47	1	0	-6,114627	-1,25516	3,606875
48	1	0	-8,282733	1,98097	1,781216
49	1	0	-8,11378	0,221228	3,52747
50	8	0	0,055838	-1,429418	2,961677
51	6	0	-1,074558	-2,147285	2,683824
52	6	0	-1,359857	-3,09348	3,823897
53	1	0	-1,272884	-2,579402	4,784546
54	1	0	-2,356735	-3,516669	3,704213
55	1	0	-0,621294	-3,901303	3,809922
56	8	0	-1,739037	-2,04034	1,682012
57	1	0	4,401325	0,475922	2,8884
58	1	0	4,687945	-2,181422	1,387765
59	1	0	2,582392	-2,80592	2,388889
60	1	0	1,727391	1,88077	1,333987
61	7	0	3,963551	0,380938	0,851854

62	16	0	4,860686	0,310383	-0,611386
63	6	0	6,04902	1,628862	-0,334141
64	8	0	5,622121	-0,943641	-0,704049
65	8	0	3,945556	0,707435	-1,680494
66	6	0	7,40995	1,328972	-0,338282
67	6	0	5,604054	2,946214	-0,188294
68	6	0	8,334572	2,363164	-0,181098
69	1	0	7,72983	0,300536	-0,46525
70	6	0	6,53891	3,96355	-0,03105
71	1	0	4,542071	3,166829	-0,193908
72	6	0	7,917454	3,690383	-0,026824
73	1	0	9,396431	2,132278	-0,180915
74	1	0	6,196973	4,988514	0,087649
75	6	0	8,918937	4,810531	0,119061
76	1	0	9,916316	4,428993	0,352911
77	1	0	8,626197	5,507327	0,911195
78	1	0	8,995451	5,390636	-0,808569
79	1	0	2,227565	-0,794068	-0,20257
80	6	0	0,700358	-0,613942	2,035838
81	16	0	1,284459	-2,876498	-1,061255
82	8	0	0,127946	-3,192328	-1,901343
83	8	0	1,278227	-3,304088	0,347908
84	8	0	1,729274	-1,414941	-1,228557
85	6	0	2,725194	-3,77576	-1,816882
86	9	0	2,526793	-5,097595	-1,725751
87	9	0	3,848442	-3,462516	-1,156769
88	9	0	2,868352	-3,447134	-3,101939

XIV

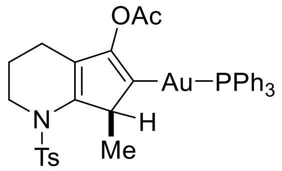

G at M06/def2tzvpp (IEFPCM, dichloromethane) = -2.622,745036260 Hartree
Correction at B3LYP/6-31G(d,p) = 0,539921
Cartesian Coordinates of the computed structure

Center Number	Atomic Number	Atomic type	Coordinates (Angstroms)		
			X	Y	Z
1	6	0	1,855254	0,101	0,021026
2	6	0	2,451235	-2,148927	0,42252
3	6	0	2,976838	-0,936947	0,105604
4	6	0	0,589109	-0,728257	0,240913
5	1	0	2,908872	-3,680468	1,819164
6	6	0	3,229897	-3,369023	0,81439
7	6	0	5,033078	-1,636282	1,188661
8	6	0	4,744354	-3,090518	0,794374
9	1	0	6,107331	-1,435493	1,193333

10	1	0	5,268423	-3,768265	1,477965
11	6	0	1,758806	1,022086	-1,205697
12	1	0	2,630205	1,670019	-1,299868
13	1	0	1,683044	0,435964	-2,124848
14	1	0	0,859885	1,641096	-1,115199
15	79	0	-1,342914	-0,07618	0,143554
16	15	0	-3,600662	0,633335	0,012061
17	6	0	-4,641554	-0,491365	-1,005606
18	6	0	-4,045849	-1,118827	-2,112512
19	6	0	-5,993221	-0,739112	-0,722621
20	6	0	-4,794971	-1,965476	-2,929471
21	1	0	-2,993556	-0,951849	-2,325665
22	6	0	-6,737506	-1,591809	-1,540103
23	1	0	-6,463193	-0,27494	0,138658
24	6	0	-6,141286	-2,203211	-2,644446
25	1	0	-4,322725	-2,446601	-3,780784
26	1	0	-7,782309	-1,780217	-1,310368
27	1	0	-6,721791	-2,868923	-3,276585
28	6	0	-3,808788	2,29952	-0,73852
29	6	0	-2,850535	3,281899	-0,438812
30	6	0	-4,877988	2,62206	-1,587078
31	6	0	-2,969841	4,56693	-0,966712
32	1	0	-2,007214	3,03447	0,200036
33	6	0	-4,99024	3,908644	-2,11825
34	1	0	-5,61766	1,86937	-1,840703
35	6	0	-4,039585	4,882076	-1,807674
36	1	0	-2,221464	5,317559	-0,730347
37	1	0	-5,81939	4,147035	-2,778147
38	1	0	-4,126988	5,880678	-2,225772
39	6	0	-4,447276	0,735431	1,64219
40	6	0	-4,065161	-0,171915	2,643761
41	6	0	-5,453925	1,673901	1,915475
42	6	0	-4,692024	-0,150869	3,889597
43	1	0	-3,268154	-0,884282	2,448766
44	6	0	-6,074756	1,694891	3,165754
45	1	0	-5,747124	2,39354	1,157448
46	6	0	-5,697443	0,782004	4,152273
47	1	0	-4,386629	-0,855581	4,657428
48	1	0	-6,850324	2,427774	3,368823
49	1	0	-6,179486	0,803025	5,125386
50	8	0	0,15114	-3,049601	0,837408
51	6	0	-0,004619	-4,086709	-0,032725
52	6	0	-0,996262	-5,084909	0,518806
53	1	0	-1,994489	-4,635727	0,536936
54	1	0	-1,007229	-5,972227	-0,113655
55	1	0	-0,739571	-5,353329	1,547002
56	8	0	0,565797	-4,178456	-1,094246

57	1	0	4,652301	-1,420014	2,193647
58	1	0	5,152551	-3,257006	-0,205791
59	1	0	2,984223	-4,20179	0,145518
60	1	0	1,986235	0,737541	0,912739
61	7	0	4,370562	-0,664706	0,278887
62	16	0	5,345445	-0,273866	-1,088281
63	6	0	6,48893	0,900107	-0,349791
64	8	0	6,147083	-1,428093	-1,527504
65	8	0	4,49493	0,426247	-2,051773
66	6	0	7,859115	0,672567	-0,458628
67	6	0	6,00062	2,057157	0,264165
68	6	0	8,749161	1,614085	0,061989
69	1	0	8,21281	-0,231634	-0,941629
70	6	0	6,900964	2,983758	0,778711
71	1	0	4,931256	2,219346	0,346379
72	6	0	8,288102	2,779844	0,684146
73	1	0	9,818478	1,437044	-0,017701
74	1	0	6,524606	3,881356	1,262961
75	6	0	9,251547	3,80735	1,228001
76	1	0	10,267378	3,408765	1,294599
77	1	0	8,953086	4,146154	2,225571
78	1	0	9,285388	4,694109	0,583381
79	6	0	1,000942	-1,993531	0,472764

TS11

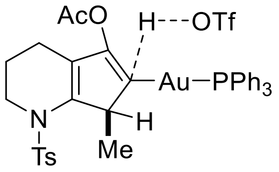

G at M06/def2tzvpp (IEFPCM, dichloromethane) = -3.584,84504780 Hartree
Freq = -1025,160
Correction at B3LYP/6-31G(d,p) = 0,563379
Cartesian Coordinates of the computed structure

Center Number	Atomic Number	Atomic type	Coordinates (Angstroms)		
			X	Y	Z
1	6	0	2,112586	-0,150533	0,173711
2	6	0	2,520128	-1,385866	-1,807353
3	6	0	3,111325	-0,533549	-0,918552
4	6	0	0,796645	-0,80454	-0,286572
5	1	0	3,077054	-3,231304	-2,699467
6	6	0	3,21601	-2,158373	-2,889864
7	6	0	5,237794	-1,544396	-1,515916
8	6	0	4,71366	-1,806876	-2,932223
9	1	0	6,305133	-1,317675	-1,525395
10	1	0	5,284881	-2,619286	-3,394056
11	6	0	1,971389	1,302757	0,64774
12	1	0	2,898065	1,686945	1,074852

13	1	0	1,693132	1,960052	-0,178784
14	1	0	1,189203	1,344154	1,411773
15	79	0	-1,080151	0,143565	-0,063407
16	15	0	-3,075285	1,364323	0,07189
17	6	0	-4,518326	0,474339	-0,637765
18	6	0	-4,344054	-0,206488	-1,855186
19	6	0	-5,772526	0,459478	-0,011738
20	6	0	-5,418796	-0,87563	-2,439457
21	1	0	-3,37152	-0,222012	-2,340369
22	6	0	-6,841463	-0,221428	-0,598945
23	1	0	-5,915708	0,97218	0,933723
24	6	0	-6,667693	-0,886182	-1,813076
25	1	0	-5,277277	-1,396665	-3,381983
26	1	0	-7,808146	-0,231987	-0,103847
27	1	0	-7,500175	-1,415926	-2,267214
28	6	0	-2,981355	2,941135	-0,869404
29	6	0	-1,757313	3,627758	-0,906926
30	6	0	-4,092088	3,482585	-1,533538
31	6	0	-1,650699	4,841143	-1,585917
32	1	0	-0,886942	3,204981	-0,413236
33	6	0	-3,979768	4,695421	-2,215894
34	1	0	-5,040515	2,954662	-1,526769
35	6	0	-2,761541	5,376363	-2,241525
36	1	0	-0,697523	5,360773	-1,611894
37	1	0	-4,844293	5,104443	-2,730769
38	1	0	-2,675641	6,317189	-2,777398
39	6	0	-3,553496	1,840405	1,77913
40	6	0	-3,2072	0,980716	2,834532
41	6	0	-4,260268	3,022887	2,050936
42	6	0	-3,579119	1,299563	4,141771
43	1	0	-2,643372	0,072177	2,64544
44	6	0	-4,623702	3,335609	3,361185
45	1	0	-4,518547	3,70278	1,245169
46	6	0	-4,285555	2,473811	4,407104
47	1	0	-3,304696	0,628917	4,950418
48	1	0	-5,166547	4,254381	3,564008
49	1	0	-4,566479	2,72175	5,426786
50	8	0	0,358838	-2,438862	-2,083154
51	6	0	-0,777035	-2,121329	-2,783812
52	6	0	-1,501282	-3,386428	-3,163317
53	1	0	-0,81002	-4,105017	-3,610517
54	1	0	-1,909655	-3,841158	-2,255818
55	1	0	-2,308258	-3,151991	-3,856738
56	8	0	-1,118604	-0,99467	-3,049259
57	1	0	5,086691	-2,418328	-0,872172
58	1	0	4,885712	-0,906637	-3,528986
59	1	0	2,747835	-1,964254	-3,86247

60	7	0	4,521028	-0,415187	-0,864048
61	16	0	5,293112	1,111695	-1,099068
62	6	0	6,516287	1,095449	0,211875
63	8	0	6,025336	1,098046	-2,37294
64	8	0	4,295607	2,148952	-0,840446
65	6	0	7,849633	1,339217	-0,112094
66	6	0	6,121425	0,904076	1,539536
67	6	0	8,798628	1,385871	0,910719
68	1	0	8,132193	1,483386	-1,148899
69	6	0	7,080832	0,952187	2,544405
70	1	0	5,082589	0,705723	1,780429
71	6	0	8,433039	1,196138	2,2483
72	1	0	9,839778	1,571625	0,661545
73	1	0	6,778617	0,796218	3,576643
74	6	0	9,457401	1,259195	3,355082
75	1	0	9,385727	0,386839	4,013268
76	1	0	9,305372	2,147064	3,980161
77	1	0	10,474859	1,302001	2,958172
78	6	0	1,135941	-1,505363	-1,422942
79	1	0	0,403697	-1,818585	0,628972
80	8	0	0,374971	-2,812466	1,323156
81	16	0	-0,810009	-3,049933	2,286413
82	8	0	-0,500205	-4,095702	3,253709
83	8	0	-1,421756	-1,796096	2,741278
84	6	0	-2,066819	-3,800661	1,132515
85	9	0	-3,187665	-4,081513	1,800832
86	9	0	-2,371403	-2,936681	0,138669
87	9	0	-1,597056	-4,920477	0,5736
88	1	0	2,455028	-0,742106	1,041206

XV

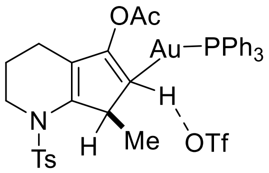

G at M06/def2tzvpp (IEFPCM, dichloromethane) = -3.584,882104510 Hartree
Correction at B3LYP/6-31G(d,p) = 0,572129

Cartesian Coordinates of the computed structure

Center Number	Atomic Number	Atomic type	Coordinates (Angstroms)		
			X	Y	Z
1	6	0	-1,179518	-1,468057	-0,249771
2	6	0	-1,590775	0,472964	-1,638454
3	6	0	-1,995001	-0,851117	-1,377695
4	6	0	-0,418855	-0,24945	0,326911
5	1	0	-2,851816	2,013486	-2,420034
6	6	0	-2,121138	1,284131	-2,786533
7	6	0	-3,728136	-0,632965	-3,106932

8	6	0	-2,767474	0,329054	-3,796752
9	1	0	-4,15765	-1,341167	-3,812812
10	1	0	-3,335005	0,890328	-4,545298
11	6	0	-0,256825	-2,605838	-0,745891
12	1	0	-0,825504	-3,432511	-1,169766
13	1	0	0,43953	-2,226378	-1,500778
14	1	0	0,329991	-2,977507	0,098911
15	79	0	1,727263	-0,248648	0,322158
16	15	0	4,083433	-0,093509	0,26703
17	6	0	4,624416	1,544101	-0,36117
18	6	0	3,793549	2,218116	-1,27157
19	6	0	5,832547	2,136854	0,03852
20	6	0	4,172308	3,460074	-1,781655
21	1	0	2,843552	1,787124	-1,573423
22	6	0	6,206183	3,379391	-0,475519
23	1	0	6,475053	1,636536	0,75646
24	6	0	5,378533	4,040947	-1,385658
25	1	0	3,512348	3,972138	-2,474998
26	1	0	7,140972	3,83276	-0,158933
27	1	0	5,669747	5,011093	-1,77767
28	6	0	4,864349	-1,347611	-0,823928
29	6	0	4,357885	-2,657796	-0,796824
30	6	0	5,937984	-1,043836	-1,673375
31	6	0	4,926387	-3,648879	-1,59543
32	1	0	3,514396	-2,898689	-0,15562
33	6	0	6,499742	-2,038793	-2,476414
34	1	0	6,330539	-0,033057	-1,715197
35	6	0	5,997688	-3,340194	-2,437344
36	1	0	4,526266	-4,657991	-1,568049
37	1	0	7,327795	-1,793181	-3,134771
38	1	0	6,434243	-4,110609	-3,066025
39	6	0	4,888333	-0,29577	1,904995
40	6	0	4,225807	0,20353	3,038405
41	6	0	6,137409	-0,916269	2,056378
42	6	0	4,811479	0,096516	4,29928
43	1	0	3,24966	0,669055	2,932555
44	6	0	6,717017	-1,025648	3,321848
45	1	0	6,653469	-1,320179	1,191079
46	6	0	6,057384	-0,518395	4,442537
47	1	0	4,290391	0,484576	5,169333
48	1	0	7,682759	-1,510486	3,430434
49	1	0	6,509314	-0,608043	5,425972
50	8	0	-0,507946	2,100675	-0,160276
51	6	0	-0,136397	3,124811	-1,024074
52	6	0	-0,493189	4,446558	-0,422796
53	1	0	-1,585938	4,484805	-0,338973
54	1	0	-0,086904	4,519149	0,590244

55	1	0	-0,110618	5,252086	-1,048566
56	8	0	0,421468	2,904074	-2,070232
57	1	0	-4,530968	-0,078548	-2,613054
58	1	0	-1,995815	-0,239155	-4,331084
59	1	0	-1,307305	1,841512	-3,25633
60	7	0	-3,007714	-1,428334	-2,059692
61	16	0	-3,830613	-2,93746	-1,582954
62	6	0	-4,265622	-2,713807	0,131718
63	8	0	-5,028726	-2,935959	-2,417402
64	8	0	-2,865737	-4,033329	-1,687847
65	6	0	-3,98546	-3,76252	1,013113
66	6	0	-4,930578	-1,557848	0,547677
67	6	0	-4,384129	-3,639121	2,342247
68	1	0	-3,463905	-4,645585	0,661967
69	6	0	-5,299119	-1,4529	1,885791
70	1	0	-5,121801	-0,725188	-0,124044
71	6	0	-5,041812	-2,487079	2,798078
72	1	0	-4,173278	-4,447585	3,036975
73	1	0	-5,77156	-0,53477	2,219267
74	6	0	-5,467111	-2,353343	4,239658
75	1	0	-6,559095	-2,392987	4,329892
76	1	0	-5,145639	-1,393497	4,65663
77	1	0	-5,051863	-3,152879	4,859017
78	6	0	-0,805163	0,845018	-0,545173
79	1	0	-0,67907	-0,039233	1,369498
80	8	0	-3,425903	1,330474	1,159892
81	16	0	-4,20882	2,13275	0,191401
82	8	0	-3,509957	3,307952	-0,381844
83	8	0	-4,964014	1,327493	-0,807785
84	6	0	-5,554033	2,88187	1,232374
85	9	0	-6,371979	3,642473	0,492896
86	9	0	-6,298207	1,91454	1,811998
87	9	0	-5,035202	3,642987	2,207829
88	1	0	-1,853619	-1,867123	0,513976

Intermediate derived from XII

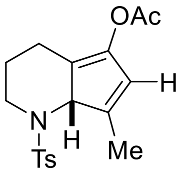

G at M06/def2tzvpp (IEFPCM, dichloromethane) = -1.451,76114953 Hartree
Correction at B3LYP/6-31G(d,p) = 0,304694
Cartesian Coordinates of the computed structure

Center Number	Atomic Number	Atomic type	Coordinates (Angstroms)		
			X	Y	Z
1	6	0	1,497206	1,681607	0,296197
2	6	0	2,21109	-0,612288	0,249284
3	6	0	1,056397	0,271471	0,669318

4	6	0	2,777522	1,616131	-0,121701
5	1	0	2,894229	-2.587.456	-0,216011
6	6	0	1,988195	-2.081.498	0,126722
7	6	0	-0,041898	-1,06802	-1,100852
8	6	0	0,794601	-2.341.625	-0,851715
9	1	0	-1,028267	-1,318732	-1,500601
10	1	0	1,173351	-2.682.998	-1,821772
11	6	0	0,639641	2,884474	0,511081
12	1	0	-0,278741	2,8176	-0,085802
13	1	0	0,328029	2,971294	1,559243
14	1	0	1,163875	3,802432	0,231893
15	8	0	4,380244	-0,272105	-0,712815
16	6	0	5,561414	0,377706	-0,472226
17	6	0	6,683462	-0,318993	-1,200343
18	1	0	6,753805	-1.360.024	-0,872037
19	1	0	6,484044	-0,329131	-2,275888
20	1	0	7,620141	0,199324	-0,999614
21	8	0	5,667483	1,35812	0,223018
22	1	0	0,448389	-0,433447	-1,846561
23	1	0	0,155514	-3.130.163	-0,449453
24	1	0	1,733918	-2.502.544	1,107236
25	7	0	-0,222936	-0,202268	0,086362
26	6	0	-1,427281	-0,682064	1,183722
27	6	0	-2,926434	-0,183351	0,333335
28	8	0	-1,476947	-2,146776	1,322304
29	8	0	-1,251036	0,165665	2,369991
30	6	0	-3,770141	-1,155754	-0,199055
31	6	0	-3,253219	1,173608	0,254514
32	6	0	-4,947303	-0,758846	-0,835881
33	1	0	-3,510061	-2,20357	-0,099085
34	6	0	-4,430631	1,550053	-0,381029
35	1	0	-2,599865	1,915858	0,699414
36	6	0	-5,293867	0,592754	-0,940603
37	1	0	-5,608585	-1,514596	-1,250552
38	1	0	-4,690964	2,603586	-0,440125
39	6	0	-6,560377	1,022289	-1,64021
40	1	0	-6,334975	1,529523	-2,585804
41	1	0	-7,134037	1,725308	-1,027323
42	1	0	-7,203	0,167783	-1,866598
43	1	0	0,957643	0,253901	1,763755
44	6	0	3,19652	0,203101	-0,166806
45	1	0	3,421594	2,448799	-0,368569

Intermediate derived from XV

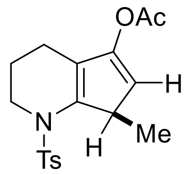

G at M06/def2tzvpp (IEFPCM, dichloromethane) = -1.451,76325427 Hartree
Correction at B3LYP/6-31G(d,p) = 0,306622
Cartesian Coordinates of the computed structure

Center Number	Atomic Number	Atomic type	Coordinates (Angstroms)		
			X	Y	Z
1	6	0	-1,426753	-1,653167	-0,01546
2	6	0	-2,096999	0,607884	-0,146357
3	6	0	-1,007396	-0,182808	0,029396
4	6	0	-2,929761	-1,55914	-0,154477
5	1	0	-2,548517	2,291004	-1,35797
6	6	0	-2,084119	2,088253	-0,382648
7	6	0	0,336954	1,604781	-0,910812
8	6	0	-0,646614	2,635497	-0,342213
9	1	0	1,358408	1,991772	-0,908383
10	1	0	-0,575412	3,567186	-0,914116
11	6	0	-1,025479	-2,623004	1,110717
12	1	0	0,052101	-2,774098	1,153272
13	1	0	-1,344928	-2,242882	2,083422
14	1	0	-1,511639	-3,589088	0,938132
15	8	0	-4,496769	0,379218	-0,343907
16	6	0	-5,670108	-0,31304	-0,466113
17	6	0	-6,820579	0,656401	-0,580162
18	1	0	-6,86962	1,286532	0,312795
19	1	0	-6,673854	1,317738	-1,438973
20	1	0	-7,749881	0,099491	-0,692707
21	8	0	-5,756524	-1,516489	-0,477465
22	1	0	0,083009	1,350989	-1,946352
23	1	0	-0,347233	2,859249	0,684916
24	1	0	-2,713514	2,60234	0,353941
25	7	0	0,313714	0,324488	-0,153952
26	6	0	1,417203	0,320581	1,174872
27	6	0	2,969262	0,03757	0,317568
28	8	0	1,503365	1,651934	1,795641
29	8	0	1,096827	-0,844786	1,999938
30	6	0	4,016327	0,938124	0,499271
31	6	0	3,137073	-1,11315	-0,458162
32	6	0	5,244143	0,684886	-0,114623
33	1	0	3,863922	1,818762	1,113075
34	6	0	4,36611	-1,348426	-1,063098
35	1	0	2,314335	-1,807592	-0,588524
36	6	0	5,43869	-0,454725	-0,902753
37	1	0	6,062994	1,385147	0,024971

38	1	0	4,500081	-2,241041	-1,668469
39	6	0	6,761885	-0,720774	-1,578316
40	1	0	6,678265	-0,609784	-2,665906
41	1	0	7,108172	-1,741486	-1,385236
42	1	0	7,534716	-0,029941	-1,232153
43	6	0	-3,273607	-0,258603	-0,222618
44	1	0	-3,588201	-2,411821	-0,132898
45	1	0	-1,013823	-2,041562	-0,961621

Hydrolized product derived from XII

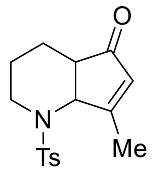

G at M06/def2tzvpp (IEFPCM, dichloromethane) = -1.299,19656641 Hartree
Correction at B3LYP/6-31G(d,p) = 0,275692
Cartesian Coordinates of the computed structure

Center Number	Atomic Number	Atomic type	Coordinates (Angstroms)		
			X	Y	Z
1	6	0	2,203229	1,864965	0,125146
2	6	0	3,122575	-0,375036	0,423032
3	6	0	1,860864	0,48832	0,690411
4	6	0	3,406515	1,867739	-0,477846
5	1	0	3,759487	-2,228878	-0,460086
6	6	0	2,814425	-1,735336	-0,214647
7	6	0	0,850055	-0,521186	-1,344064
8	6	0	1,938282	-1,619027	-1,475815
9	1	0	-0,11133	-0,858684	-1,737526
10	1	0	2,564577	-1,383164	-2,343155
11	6	0	1,248272	3,003965	0,265493
12	1	0	0,273359	2,735028	-0,156368
13	1	0	1,078185	3,230873	1,325831
14	1	0	1,61292	3,907529	-0,228404
15	1	0	1,129585	0,361137	-1,928766
16	1	0	1,470717	-2,587903	-1,670131
17	1	0	2,300126	-2,359031	0,522532
18	7	0	0,639037	-0,033732	0,0341
19	16	0	-0,44733	-0,924995	0,988403
20	6	0	-2,03471	-0,367406	0,364748
21	8	0	-0,351773	-2,362749	0,694372
22	8	0	-0,28413	-0,431363	2,36199
23	6	0	-2,80665	-1,223923	-0,417024
24	6	0	-2,49696	0,907488	0,705098
25	6	0	-4,049088	-0,786549	-0,879813
26	1	0	-2,441684	-2,219894	-0,641531
27	6	0	-3,73745	1,325047	0,237393
28	1	0	-1,898309	1,550037	1,341474
29	6	0	-4,531308	0,488927	-0,566192

30	1	0	-4,654418	-1,452853	-1,488019
31	1	0	-4,103121	2,312949	0,5049
32	6	0	-5,869126	0,96572	-1,07684
33	1	0	-5,745078	1,752901	-1,829944
34	1	0	-6,475762	1,387026	-0,268495
35	1	0	-6,435745	0,152326	-1,536851
36	1	0	1,630251	0,565408	1,755824
37	6	0	4,02991	0,528774	-0,429988
38	1	0	3,880174	2,715255	-0,961508
39	8	0	5,073276	0,184531	-0,955563
40	1	0	3,658083	-0,542268	1,366133

Hydrolized product derived from XV

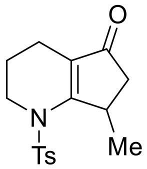

G at M06/def2tzvpp (IEFPCM, dichloromethane) = -1.299,20731134 Hartree
Correction at B3LYP/6-31G(d,p) = 0,27571

Cartesian Coordinates of the computed structure

Center Number	Atomic Number	Atomic type	Coordinates (Angstroms)		
			X	Y	Z
1	6	0	2,31059	1,607328	0,34179
2	6	0	2,956791	-0,55992	-0,501071
3	6	0	1,913067	0,19147	-0,065159
4	6	0	3,834346	1,623339	0,066567
5	1	0	3,054552	-2,063975	-2,015927
6	6	0	2,884528	-1,991989	-0,932806
7	6	0	0,416873	-1,55923	-0,88109
8	6	0	1,515897	-2,576192	-0,560528
9	1	0	-0,5731	-1,959739	-0,658489
10	1	0	1,321003	-3,505192	-1,10652
11	6	0	1,545403	2,699834	-0,421455
12	1	0	1,659423	2,578008	-1,504493
13	1	0	0,482989	2,685425	-0,170878
14	1	0	1,935269	3,685884	-0,150063
15	1	0	0,433534	-1,296244	-1,94516
16	1	0	1,471826	-2,807007	0,508955
17	1	0	3,702382	-2,553551	-0,467837
18	7	0	0,587523	-0,284179	-0,130011
19	16	0	-0,464318	-0,120491	1,228288
20	6	0	-2,062141	-0,0047	0,420544
21	8	0	-0,456168	-1,360805	2,013689
22	8	0	-0,142797	1,156259	1,868187
23	6	0	-3,075823	-0,872406	0,823282
24	6	0	-2,302144	0,99231	-0,5297
25	6	0	-4,344432	-0,741632	0,257278

26	1	0	-2,866517	-1,634004	1,565983
27	6	0	-3,571524	1,103985	-1,085176
28	1	0	-1,505423	1,660821	-0,83612
29	6	0	-4,612619	0,241668	-0,701897
30	1	0	-5,137164	-1,415733	0,569011
31	1	0	-3,761698	1,87515	-1,826796
32	6	0	-5,980612	0,371898	-1,325327
33	1	0	-5,963906	0,063462	-2,377292
34	1	0	-6,331067	1,408841	-1,29979
35	1	0	-6,71739	-0,248017	-0,808684
36	6	0	4,194768	0,223721	-0,436632
37	1	0	4,101907	2,358613	-0,700702
38	8	0	5,315096	-0,172678	-0,718344
39	1	0	4,429087	1,861902	0,953711
40	1	0	2,113865	1,743351	1,408588

Triflate
G at M06/def2tzvpp (IEFPCM, dichloromethane) = -961,69255067 Hartree
Correction at B3LYP/6-31G(d,p) = -0,005245
Cartesian Coordinates of the computed structure

	Atomic Number	Atomic type	Coordinates (Angstroms)		
			X	Y	Z
1	16	0	-0,926091	-0,000067	0,000005
2	8	0	-1,243893	-0,725492	-1,251203
3	8	0	-1,243951	-0,720939	1,253833
4	6	0	0,941132	0,000193	0,000005
5	8	0	-1,244288	1,446212	-0,002664
6	9	0	1,445401	0,630037	1,084789
7	9	0	1,445397	0,624649	-1,087863
8	9	0	1,445615	-1,254501	0,003092

Triflic acid
G at M06/def2tzvpp (IEFPCM, dichloromethane) = -962,10513635 Hartree
Correction at B3LYP/6-31G(d,p) = 0,005457
Cartesian Coordinates of the computed structure

Center Number	Atomic Number	Atomic type	Coordinates (Angstroms)		
			X	Y	Z
1	16	0	-0,852806	-0,147119	0,075807
2	8	0	-1,214883	-1,376014	-0,597745
3	8	0	-1,261655	0,182482	1,433785
4	6	0	1,00822	0,009671	-0,001689
5	8	0	-1,255732	1,091951	-0,903697
6	9	0	1,361972	1,224792	0,421452
7	9	0	1,426279	-0,169897	-1,249777
8	9	0	1,540461	-0,914701	0,792768
9	1	0	-1,504677	1,846792	-0,341507

Au_triflate

G at M06/def2tzvpp (IEFPCM, dichloromethane) = -2.133,12563169 Hartree

Correction at B3LYP/6-31G(d,p) = 0,239437

Cartesian Coordinates of the computed structure

Center Number	Atomic Number	Atomic type	Coordinates (Angstroms)		
			X	Y	Z
1	79	0	-0,680615	-0,350379	-0,647158
2	15	0	1,457012	0,034654	0,022213
3	6	0	1,804248	-0,831658	1,599155
4	6	0	0,738711	-1,098902	2,475053
5	6	0	3,106648	-1,221029	1,950744
6	6	0	0,982228	-1,732407	3,694143
7	1	0	-0,279164	-0,833454	2,201556
8	6	0	3,340805	-1,855064	3,171156
9	1	0	3,933497	-1,042556	1,270123
10	6	0	2,28032	-2,108473	4,04393
11	1	0	0,152159	-1,941331	4,361826
12	1	0	4,34985	-2,157371	3,435386
13	1	0	2,46465	-2,608081	4,990462
14	6	0	1,791854	1,815596	0,304952
15	6	0	1,219906	2,752351	-0,571608
16	6	0	2,605896	2,262115	1,356232
17	6	0	1,471958	4,113243	-0,405719
18	1	0	0,568972	2,416757	-1,374142
19	6	0	2,850785	3,626903	1,520027
20	1	0	3,038589	1,550958	2,052295
21	6	0	2,287466	4,552019	0,640066
22	1	0	1,021686	4,830083	-1,085591
23	1	0	3,477157	3,96508	2,340109
24	1	0	2,475368	5,613211	0,773774
25	6	0	2,721214	-0,539769	-1,175509
26	6	0	2,470763	-1,712627	-1,906186
27	6	0	3,930631	0,145672	-1,363151
28	6	0	3,423613	-2,198215	-2,800418
29	1	0	1,52797	-2,237876	-1,780824
30	6	0	4,879323	-0,343581	-2,263099
31	1	0	4,129112	1,062702	-0,817534
32	6	0	4,628512	-1,514624	-2,979753
33	1	0	3,219959	-3,104026	-3,363348
34	1	0	5,81147	0,19483	-2,406376
35	1	0	5,36646	-1,889982	-3,682513
36	8	0	-4,837786	-1,672976	-0,431156
37	16	0	-3,681558	-0,873806	-0,041074
38	8	0	-3,008693	-1,136667	1,241015
39	8	0	-2,671229	-0,731698	-1,206915
40	6	0	-4,291923	0,882569	0,089428
41	9	0	-3,26209	1,709022	0,358252
42	9	0	-4,859116	1,273013	-1,054585

43	9	0	-5,184961	0,983305	1,077892

Experimental section

General information

Anhydrous solvents were prepared according to the standard techniques. Commercially available reagents were used without further purification. Melting points were recorded on a Büchi B-540 apparatus and are uncorrected. Chromatographic separations were performed under pressure on silica gel (Merck 70–230 mesh) by using flash column techniques; R_f values refer to TLC carried out on 0.25 mm silica gel plates (F_{254}) with the same eluent as indicated for column chromatography. ^{1}H NMR (400 MHz) and ^{13}C NMR (100.4 MHz) spectra were recorded on Varian Inova and Mercury (400 MHz) spectrometers in the specified deuterated solvent at 25 °C. Solvent reference lines were set at 7.26 ppm and 77.00 ppm ($CDCl_3$) in the ^{1}H and ^{13}C NMR spectra, respectively. Mass spectra were recorded either by direct inlet of a 10 ppm solution in CH_3OH on a LCQ FleetTM Ion Trap LC/MS system (Thermo Fisher Scientific) with electrospray ionization (ESI) interface in the positive ion mode or by EI at 70 eV or by methanol CI on a Varian GC/MS Saturn 2200 instrument equipped with a CP-sil8 Varian column. HRMS analyses were performed under conditions of ESIMS through direct infusion of a 1 uM solution in MeOH in a TripleTOF® 5600+ mass spectrometer (Sciex, Framingham, MA, U.S.A.), equipped with a DuoSpray® interface operating with an ESI probe. Microanalyses were carried out with a CHN Thermo FlashEA 1112 Series elemental analyzer.

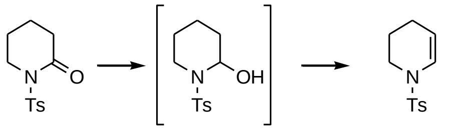

1-(Toluene-4-sulfonyl)-1,2,3,4-tetrahydropyridine (10)

A solution of **8** (500 mg, 2 mmol) in DCM (10 mL) was cooled to −78 °C, and a 1 M solution of DIBAL-H (2.2 mL, 2.2 mmol) was slowly added, keeping the temperature bellow −70 °C. The reaction mixture was stirred below −70 °C and after 1 h, MeOH (1 mL) was added, followed by a saturated solution of Rochelle salt. The formed two-phase system was stirred at room temperature for 1 h. The layers were separated, 1-(toluene-4-sulfonyl)-piperidin-2-ol was extracted with DCM (2 × 15 mL), and the organic layer was dried over anhydrous Na_2CO_3 for 30 min. After filtration and evaporation of the solvent, the obtained 1-(toluene-4-sulfonyl)-piperidin-2-ol was dissolved in DCM (10 mL), and DMAP (9.6 mg, 0.78 mmol) and Et_3N (0.83 mL, 6 mmol) were added. This solution was cooled to 0 °C, and MsCl (0.3 mL, 3 mmol) was added. The reaction was stirred at room temperature. After 3 h, a saturated solution of NH_4Cl (10 mL) was added, and the product extracted with DCM (3 ×10 mL). The organic extracts were dried over anhydrous Na_2CO_3 for 30 min. After filtration and evaporation of the solvent, column chromatography (*n*-hexane/EtOAc 5:1; R_f = 0.40) afforded pure 1-(toluene-4-sulfonyl)-1,2,3,4-tetrahydropyridine (**10**, 333 mg, 70%) as white wax. ^{1}H NMR (400 MHz, $CDCl_3$): δ = 7.69 (d, J = 7.6 Hz, 2H, Ts), 7.33 (d, J = 7.6 Hz, 2H, Ts), 6.66 (d, J = 8.4 Hz, 1H, 2-H), 4.98 (bs, 1H, 3-H), 3.39 (t, J = 3.6 Hz, 2H, 6-H), 2.44 (s, 3H, CH_3-Ts), 1.92 (bs, 2H, 4-H), 1.68 – 1.65 (m, 2H, 5-H) ppm. Spectroscopical data correspond to the literature values.[1]

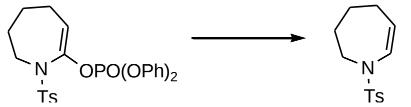

1-(Toluene-4-sulfonyl)-2,3,4,5-tetrahydro-1*H*-azepine (18)

A solution of **17** (975 mg, 1.94 mmol), $Pd(OAc)_2$ (21.8 mg, 0.097 mmol), and PPh_3 (49.8 mg, 0.19 mmol) in DME (5 mL) was prepared under a nitrogen atmosphere. This solution was added dropwise, under a nitrogen atmosphere, to a solution

of formic acid (0.147 mL, 3.9 mmol) and Et_3N (0.75 mL, 5.82 mmol) in DME (5 mL). The reaction mixture was refluxed (at 85 °C) for 30 min and then cooled to room temperature. Water (10 mL) was added, and the product was extracted with EtOAc (3 × 10 mL). The organic phase was dried over Na_2SO_4 and concentrated. Column chromatography (*n*-hexane/EtOAc 10:1; R_f = 0.22) afforded the desired product **18** (290 mg, 62%) as white solid. M.p. = 59.2 – 60.8 °C. ^{1}H NMR (400 MHz, $CDCl_3$): δ = 7.70 (d, J = 8.2 Hz, 2H, Ts), 7.29 (d, J = 8.2 Hz, 2H, Ts), 6.41 (d, J = 8.8 Hz, 1H, 7-H), 5.09 (dt, J = 8.8, 5.7 Hz, 1H, 6-H), 3.50 (t, 2H, J = 6.1 Hz, 2-H), 2.42 (s, 3H, C*H*$_3$-Ts), 2.11 – 2.06 (m, 2H, 5-H), 1.71 – 1.68 (m, 2H, 3-H), 1.50 – 1.44 (m, 2H, 4-H) ppm. ^{13}C NMR (100.4 MHz, $CDCl_3$): δ = 143.4 (s, Ts), 136.7 (s, Ts), 129.8 (d, 2C, Ts), 129.2 (d, C-7), 126.9 (d, 2C, Ts), 116.8 (d, C6), 49.7 (t, C2), 28.9 (t, C5), 26.1 (t, C3), 24.9 (t, C4), 21.5 (q, *C*H$_3$-Ts). MS (ESI) *m/z* (%): 252 ([M+1]$^+$, 100), 274 ([M+Na]$^+$, 32), 524 ([2M+Na]$^+$, 11). Elemental analysis calcd (%) for $C_{13}H_{17}NO_2S$: C 62.12, H 6.82, N 5.57; found C 61.63, H 6.58, N 5.63.

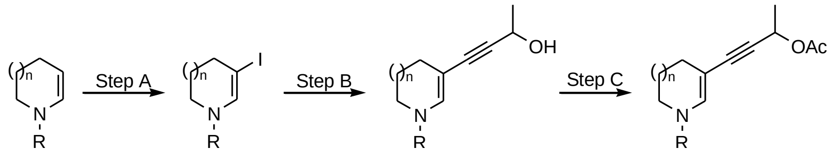

General procedure for the synthesis of enesulfonamide-derived enynyl acetates

Step A: Iodination of enesulfonamides

The enesulfonamide (1 mmol) and $NaOCH_3$ (105 mg, 2 mmol) were suspended in MeOH (4 mL), and a 1 M solution of ICl in DCM (1.1 mL, 1.1 mmol) was added dropwise. The resulting brown suspension was stirred at room temperature for 30 min, and then a 10% aqueous solution of $Na_2S_2O_3$ was added and stirring continued for 30 min. The layers were separated; the organic layer was dried over Na_2SO_4, filtered, and concentrated to give a brown oil. The obtained oil was dissolved in toluene (8 mL), and a solution of TFA (11 µL, 0.15 mmol) in toluene (20 µL) was added. The obtained purple solution was submersed into an oil bath preheated to 140 °C. After 5 min, the reaction flask was put on ice and immediately, a solution of Et_3N (62 µL, 0.5 mmol) in toluene (200 µL) was added. The reaction mixture was concentrated and the crude was purified by flash chromatography to give the corresponding β-iodoenesulfonamide.

Step B: Sonogashira coupling

The β-iodo-enesulfonamide (1 mmol), CuI (19 mg, 0.1 mmol), and $(Ph_3P)_2PdCl_2$ (35 mg, 0.05 mmol) were dissolved in an anhydrous Et_2NH/DMF 4:1 mixture (10 mL), and the alkyne (1.2 mmol) was added under a nitrogen atmosphere. The reaction mixture was heated at 40 °C for 10 min, and water (15 mL) was added. The product was extracted with Et_2O (3 × 10 mL), and the combined organic extracts were dried over anhydrous K_2CO_3 for 30 min. After filtration and evaporation of the solvent, the crude was purified by flash chromatography, and the so obtained enynyl alcohol was used in the next step.

Step C: acetylation

A solution of the enynyl alcohol (1 mmol), DMAP (24 mg, 0.2 mmol), and Et_3N (0.38 mL, 3 mmol) in DCM (4 mL) was cooled by ice bath, and Ac_2O (0.19 mL, 2 mmol) was added. The reaction mixture was stirred at room temperature and analysed by TLC analysis. When the conversion was complete, a saturated solution of $NaHCO_3$ (10 mL) was added and the product extracted with DCM (3 × 10 mL). The combined organic extracts were dried over anhydrous K_2CO_3 for 30 min. After filtration and evaporation of the solvent, the crude was purified by flash chromatography and stored at 4 °C as a 0.1 M solution in the eluent containing 1% Et_3N until use.

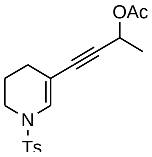

Acetic acid 1-methyl-3-[1-(toluene-4-sulfonyl)-1,4,5,6-tetrahydro-pyridin-3-yl]-prop-2-ynyl ester (14)

Compound **14** was prepared according to the above described procedure starting from **10** (230 mg, 1 mmol). Chromatography (*n*-hexane/EtOAc 3:2; R_f = 0.22) afforded **12** (327 mg, 77%) contaminated by an unknown impurity. ^{1}H NMR (400 MHz, $CDCl_3$): *δ* = 7.65 (d, *J* = 8.3 Hz, 2H, Ts), 7.32 (d, *J* = 10.8 Hz, 2H, Ts), 7.12 (s, 1H, 2-H), 3.22 – 3.12 (m, 2H, 6-H), 2.43 (s, 3H, CH_3-Ts), 2.32 (td, *J* = 6.3, 2.7 Hz, 2H, 4-H), 1.76 – 1.66 (m, 2H, 5-H) ppm. ^{13}C NMR (100.4 MHz, $CDCl_3$): *δ* = 143.9 (s, Ts), 134.6 (s, Ts), 130.4 (d, C-2), 129.9 (d, 2C, Ts), 126.9 (d, 2C, Ts), 87.7 (s, C3), 42.2 (t, C6), 34.0 (t, C4), 23.0 (t, C5), 21.4 (q, CH_3-Ts) ppm. MS (ESI) *m/z* (%): 364 ($[M+1]^+$, 100), 385 ($[M+Na]^+$, 8).

Sonogashira coupling of the β-iodoenesulfonamide **12** (193 mg, 0.9 mmol) and (±)-3-butyn-2-ol after chromatography (*n*-hexane/EtOAc 3:1 + 1% Et_3N; R_f = 0.16) afforded the enynyl alcohol **13**, which was used immediately in the next step (220 mg, 80%). ^{1}H NMR (400 MHz, $CDCl_3$): *δ* = 7.66 (d, *J* = 8.3 Hz, 2H, Ts), 7.31 (d, *J* = 8.3 Hz, 2H, Ts), 7.06 (s, 1H, 2-H), 4.63 (q, *J* = 6.6 Hz, 1H, 3'-H), 3.35 – 3.31 (m, 2H, 6-H), 2.43 (s, 3H, CH_3-Ts), 2.05 – 2.02 (m, 4-H), 1.77 (bs, 1H, OH), 1.71 – 1.65 (m, 2H, 5-H), 1.45 (d, *J* = 6.6 Hz, 3H, 3'-CH_3) ppm. ^{13}C NMR (100.4 MHz, $CDCl_3$): *δ* = 144.0 (s, Ts), 134.8 (s, Ts), 130.7 (d, C2), 129.9 (d, 2C, Ts), 127.0 (d, 2C, Ts), 100.9 (s, C3), 89.4 (s, C1'), 83.7 (C2'), 58.9 (C3'), 43.6 (t, C6), 25.5 (C4), 24.5 (q, *C*H_3-3'), 21.6 (q, CH_3-Ts), 20.6 (t, C-5) ppm. MS (ESI) *m/z* (%): 306 ($[M+1]^+$, 100), 328 ($[M+Na]^+$, 31), 633 ($[2M+Na]^+$, 35).

According to the above described procedure, compound **13** was subjected to acetylation. After chromatography (*n*-hexane/EtOAc 6:1 + 1% Et_3N; R_f = 0.18), pure **14** was obtained as colorless oil (210 mg, 84%). ^{1}H NMR (400 MHz, $CDCl_3$): *δ* = 7.66 (d, *J* = 8.3 Hz, 2H, Ts), 7.32 (d, *J* = 8.0 Hz, 2H, Ts), 7.09 (s, 1H, 2-H), 5.69 – 5.44 (m, 1H, 3'-H), 3.43 – 3.21 (m, 2H, 6-H), 2.43 (s, 3H, CH_3-Ts), 2.08 (s, 3H, CH_3-Ac), 2.04 (td, *J* = 6.2, 1.3 Hz, 2H, 4-H), 1.73 – 1.63 (m, 2H, 5-H), 1.49 (d, *J* = 6.7 Hz, 3H, 3'-CH_3) ppm. ^{13}C NMR (100.4 MHz, $CDCl_3$): *δ* = 169.9 (s, CO), 144.1 (s, Ts), 134.8 (s, Ts), 131.3 (d, C2), 129.9 (d, 2C, Ts), 127.0 (d, 2C, Ts), 100.5 (C3), 85.8 (s, C1'), 84.4 (s, C2'), 60.9 (s, C3'), 43.4 (t, C6), 25.3 (q, CH_3-Ac), 21.6 (q, CH_3-3'), 21.5 (q, CH_3-Ts), 21.1 (t, C-4), 20.6 (t, C-5) ppm. MS (ESI) *m/z* (%): 288 ($[C_{16}H_{18}NO_2S]^+$, 100), 369 ($[M+Na]^+$, 17). Elemental analysis calcd (%) for $C_{18}H_{21}NO_4S$: C 62.23, H 6.09, N 4.03; found C 62.36, H 6.10. N 3.88.

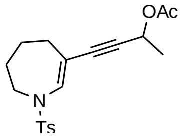

Acetic acid 1-methyl-3-[1-(toluene-4-sulfonyl)-4,5,6,7-tetrahydro-1*H*-azepin-3-yl]-prop-2-ynyl ester (20)

Compound **19** was prepared according to the above described procedure starting from **17** (156 mg, 0.62 mmol). Chromatography (*n*-hexane/EtOAc 3:2; R_f = 0.22) afforded **19** (201 mg, 85%) contaminated by unknown impurity. ^{1}H NMR (400 MHz, $CDCl_3$): *δ* = 7.70 (d, *J* = 8.2 Hz, 2H, Ts), 7.32 (d, *J* = 8.2 Hz, 2H, Ts), 6.92 (s, 1H, 7-H), 3.60 – 3.52 (m, 2H, 2-H), 2.72 – 2.59 (m, 2H, 5-H), 2.44 (s, 3H, C*H*$_3$-Ts), 1.78 – 1.66 (m, 2H, 3-H), 1.47 (dt, *J* = 12.2, 6.1 Hz, 2H, 4-H) ppm.

Sonogashira coupling of **19** (193 mg, 0.51 mmol) and (±)-3-butyn-2-ol after chromatography (*n*-hexane/EtOAc 3:1 + 1% Et_3N; R_f = 0.16) afforded the enynyl alcohol, which was used immediately in the next step (115 mg, 71%). ^{1}H NMR (400 MHz, $CDCl_3$): *δ* = 7.71 (d, *J* = 8.2 Hz, 2H, Ts), 7.32 (d, *J* = 8.2 Hz, 2H, Ts), 6.84 (s, 1H, 2-H), 4.62 (q, *J* = 6.6 Hz, 1H, 1'-H), 3.55 (t, *J* = 6.3 Hz, 2H, 7-H), 2.43 (s, 3H, C*H*$_3$-Ts), 2.35 – 2.21 (t, *J* = 6.2 Hz, 2H, 4-H), 1.72 (dt, *J* = 12.2, 6.3 Hz, 3H, 6-H and OH), 1.55 (dt, *J* = 12.2, 6.2 Hz, 2H, 5-H), 1.46 (d, *J* = 6.6 Hz, 3H, 1'-C*H*$_3$) ppm.

According to the above described procedure, the enynyl alcohol was subjected to acetylation. After chromatography (*n*-hexane/EtOAc 6:1 + 1% Et_3N; R_f = 0.18), pure **20** was obtained as colorless oil (100 mg, 84%). ^{1}H NMR (400 MHz, $CDCl_3$): *δ* = 7.69 (d, *J* = 8.2 Hz, 2H, Ts), 7.31 (d, *J* = 8.2 Hz, 2H, Ts), 6.86 (s, 1H, 2-H), 5.54 (q, *J* = 6.7 Hz, 1H, 3'-H), 3.46 (t, *J* = 6.1 Hz, 2H, 7-H), 2.42 (s, 3H, C*H*$_3$-Ts), 2.27 (t, *J* = 6.0 Hz, 2H, 4-H), 2.06 (s, 3H, C*H*$_3$-Ac), 1.70 (dt, *J* = 12.2, 6.1 Hz, 2H,

6-H), 1.54 (dt, J = 12.2, 6.0 Hz, 2H, 5-H), 1.47 (d, J = 6.7 Hz, 3H, 3'-CH_3). ppm. ^{13}C NMR (100.4 MHz, $CDCl_3$): δ = 169.9 (s, CO), 143.8 (s, Ts), 136.1 (s, Ts), 135.7 (d, C2), 129.8 (d, 2C, Ts), 126.7 (d, 2C, Ts), 109.5 (s, C3), 85.8 (s, C2'), 85.4 (d, C1'), 60.9 (d, C3'), 48.8 (t, C7), 31.2 (t, C6), 28.0 (t, C4), 24.03 (t, C5), 21.4 (q, CH_3-Ac), 21.5 (q, CH_3-Ts), 21.1 (q, CH_3-3') ppm. MS (ESI) *m/z* (%): 302 ([M-OAc]$^+$, 100), 384 ([M+Na]$^+$, 49), 744 ([2M+Na]$^+$, 6). Elemental analysis calcd (%) for $C_{19}H_{23}NO_4S$: C 63.13, H 6.41, N 3.88; found C 63.44, H 6.12, N 3.92.

General procedure for the gold(I)-catalyzed Nazarov reaction

The precatalyst LAuCl (3 mol %, 0.006 mmol) was dissolved in DCM (1.5 mL), and the silver salt (3 mol %, 0.006 mmol) was added. The formed suspension was left to stir at room temperature under a nitrogen atmosphere. After 20 min, a solution of the enynyl acetate **7** (0.2 mmol) in DCM (2.5 mL) was added, and the reaction mixture was stirred at room temperature. The progress of the reaction was followed by TLC analysis. After complete consumption of the enynyl acetate (1–5 h), the reaction mixture was left to stir at room temperature overnight. Water (5 mL) was added and the product extracted with DCM (3 × 5 mL). The combined organic extracts were dried over anhydrous Na_2SO_4, filtered, and concentrated. The oily residue was purified by flash chromatography to give the corresponding cyclopenta-fused piperidine derivative.

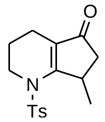

7-Methyl-1-(toluene-4-sulfonyl)-1,2,3,4,6,7-hexahydro-[1]pyrindin-5-one (15)

Compound **15** was prepared according to the general procedure for the Au(I)-catalyzed Nazarov reaction starting from **14** (52 mg, 0.16 mmol). The reaction was carried out with 5 mol % $Ph_3PAuCl/AgSbF_6$ in DCM at room temperature. Chromatography (n-hexane/EtOAc 3:1; R_f = 0.15) afforded pure product **15** (32 mg, 66%) as white solid. ^{1}H NMR (400 MHz, $CDCl_3$): δ = 7.63 (d, J = 14.8 Hz, 2H, Ts), 7.33 (d, J = 8.4 Hz, 2H, Ts), 3.83 (ddd, J = 13.6, 5.0. 3.5 Hz, 1H, 2-H), 3.67 (p, J = 6.7 Hz, 1H, 7-H), 3.41 – 3.27 (m, 1H, 2-H), 2.71 (dd, J = 18.4, 6.8 Hz, 1H, 6-H), 2.48 – 2.37 (m, 4H, CH_3-Ts and 4-H), 2.13 – 1.95 (m, 2H, 6-H and 5-H), 1.72 – 1.54 (m, 1H, 5-H), 1.41 (d, J = 6.8 Hz, 2H, 7-CH_3) ppm. ^{13}C NMR (100.4 MHz, $CDCl_3$): δ = 204.1 (s, CO), 170.1 (s, C7a), 144.8 (s, Ts), 135.3 (s, Ts), 130.2 (d, 2C, Ts), 127.1 (d, 2C, Ts), 123.8 (s, C4a), 47.9 (d, C6), 43.0 (t, C2), 35.4 (d, C7), 22.1 (t, C5), 21.6 (q, CH_3-Ts), 19.3 (t, C4), 18.3 (q, CH_3-7) ppm. MS (ESI) *m/z* (%): 306 ([M+1]+, 100), 328 ([M+Na]$^+$, 15), 633 ([2M+Na]$^+$, 35). Elemental analysis calcd (%) for $C_{16}H_{19}NO_3S$: C 62.93, H 6.27, N 4.59; found: C 62.70. H 6.33, N 4.54.

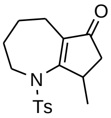

8-Methyl-1-(toluene-4-sulfonyl)-2,3,4,5,7,8-hexahydro-1*H*-cyclopenta[b]azepin-6-one (21)

Compound **21** was prepared according to the general procedure for the Au(I)-catalyzed Nazarov reaction starting from **20** (50 mg, 0.14 mmol). The reaction was carried out with 5 mol % $Ph_3PAuCl/AgSbF_6$ in DCM at room temperature. Chromatography (*n*-hexane/EtOAc 3:1; R_f = 0.15) afforded pure product **21** (24 mg, 54%) as white solid. M.p. = 118.4 – 119.0 °C. ^{1}H NMR (400 MHz, $CDCl_3$): δ = 7.72 (d, J = 8.3 Hz, 2H, Ts), 7.32 (d, J = 8.3 Hz, 2H, Ts), 4.31 (dt, J = 15.0. 3.7 Hz, 1H, 2-H), 3.62 – 3.49 (m, 1H, 8-H), 3.12 (ddd, J = 15.3, 12.3, 3.3 Hz, 1H, 2-H), 2.78 (dd, J = 19.5, 8.8 Hz, 1H, 7-H), 2.47 – 2.33 (m, 4H, CH_3-Ts and 5-H), 2.09 (dd, J = 19.5, 1.7 Hz, 1H, 7-H), 1.88 – 1.73 (m, 1H, 3-H), 1.67 – 1.45 (m, 3H, 3-H and 4-H), 1.41 – 1.32 (m, 1H, 5-H), 1.21 (d, J = 7.0 Hz, 3H, 8-CH_3) ppm. ^{13}C NMR (100.4 MHz, $CDCl_3$): δ = 205.7 (s, C6), 174.3 (s, C8a), 144.3 (s, Ts), 137.2 (s, Ts), 133.7 (s, C6a), 129.9 (d, 2C, Ts), 126.9 (d, 2C, Ts), 52.4 (t, C7), 43.0 (t, C2), 35.6 (t, C8), 28.1 (d, C5), 22.9 (t, C3), 21.6 (t, C4), 21.6 (q, CH_3-Ts), 20.3 (q, CH_3-8) ppm. MS (ESI) *m/z* (%): 320 ([M+1]$^+$, 100). Elemental analysis calcd (%) for $C_{17}H_{21}NO_3S$: C 63.92, H 6.63, N 4.39; found: C 62.93, H 6.53, N 4.06.

References

1. M. Shao, L. Zheng, W. Quiao, J. Wang, J. Wang, *Adv. Synth. Catal*. **2012**, *354*, 2743 – 2750

^{1}H and ^{13}C NMR spectra

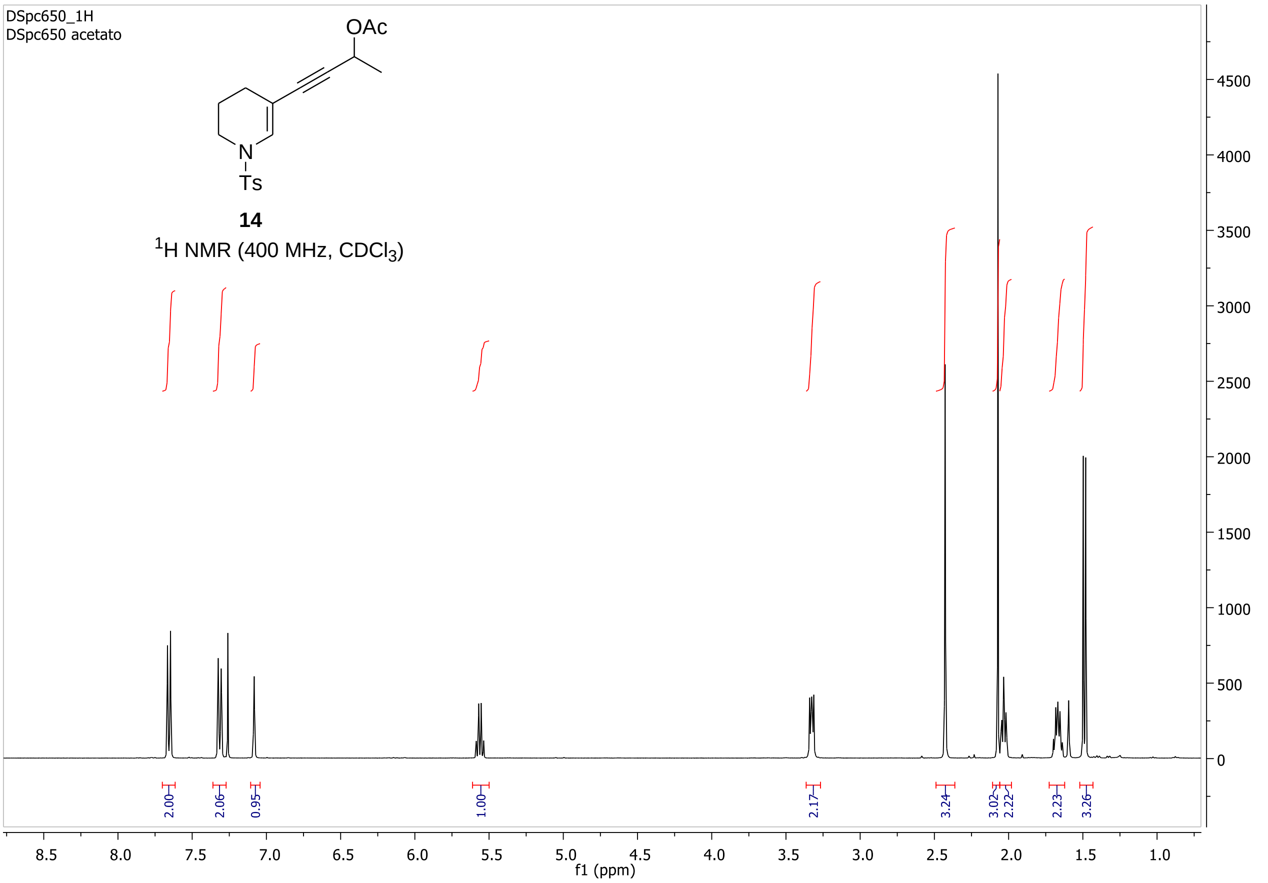

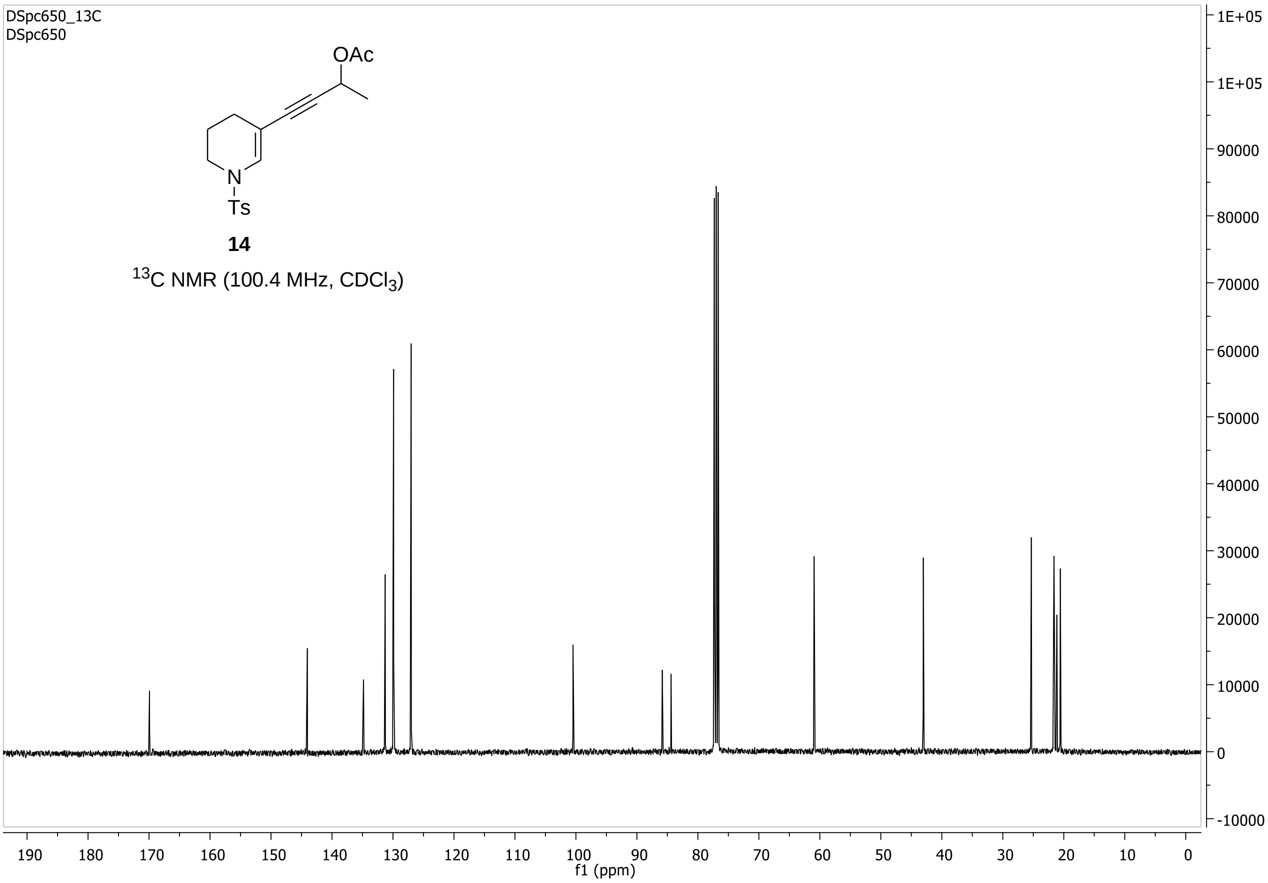

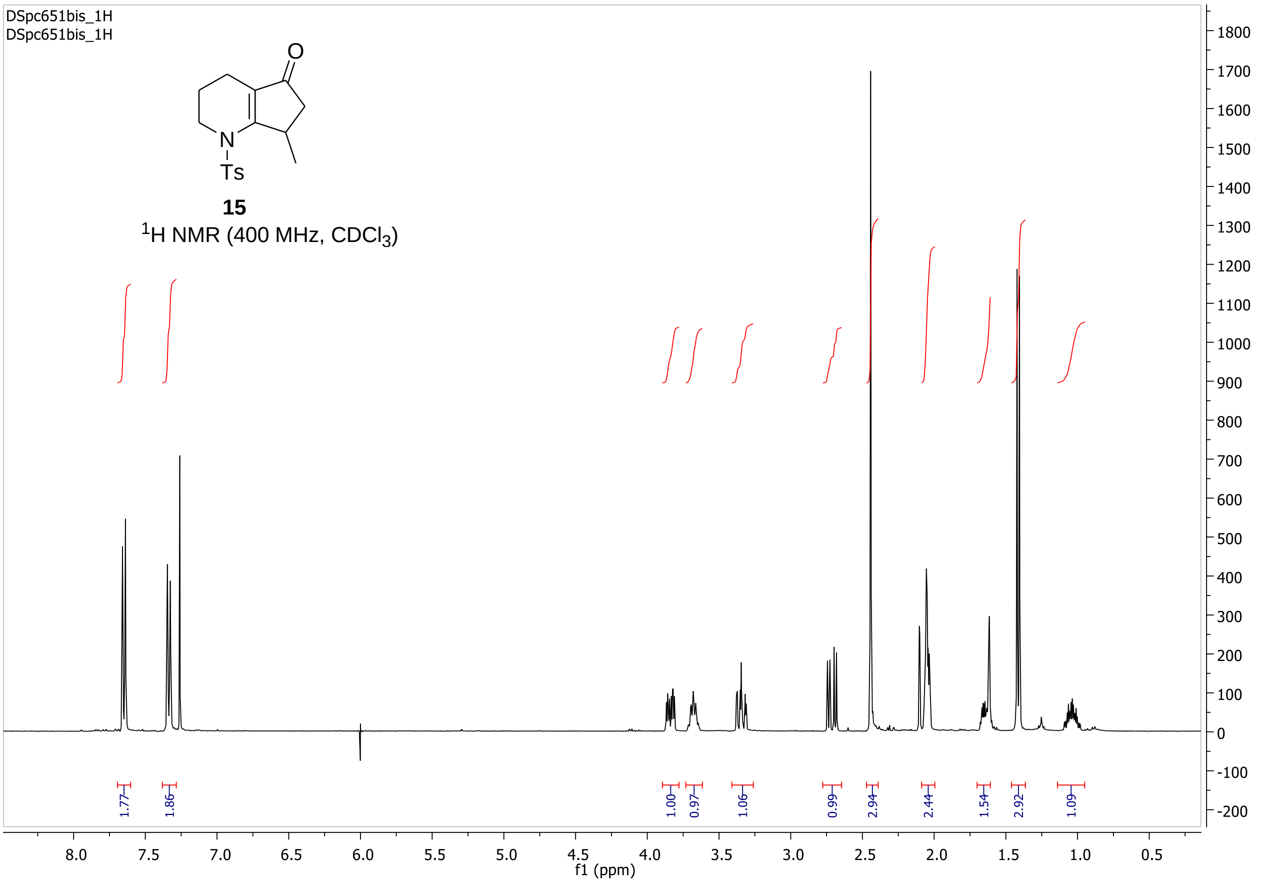
DSpc651bis_1H
DSpc651bis_1H
O
N
Ts
15
1H NMR (400 MHz, CDCl3)
1.77
1.86
1.00
0.97
1.06
0.99
2.94
2.44
1.54
2.92
1.09
f1 (ppm)

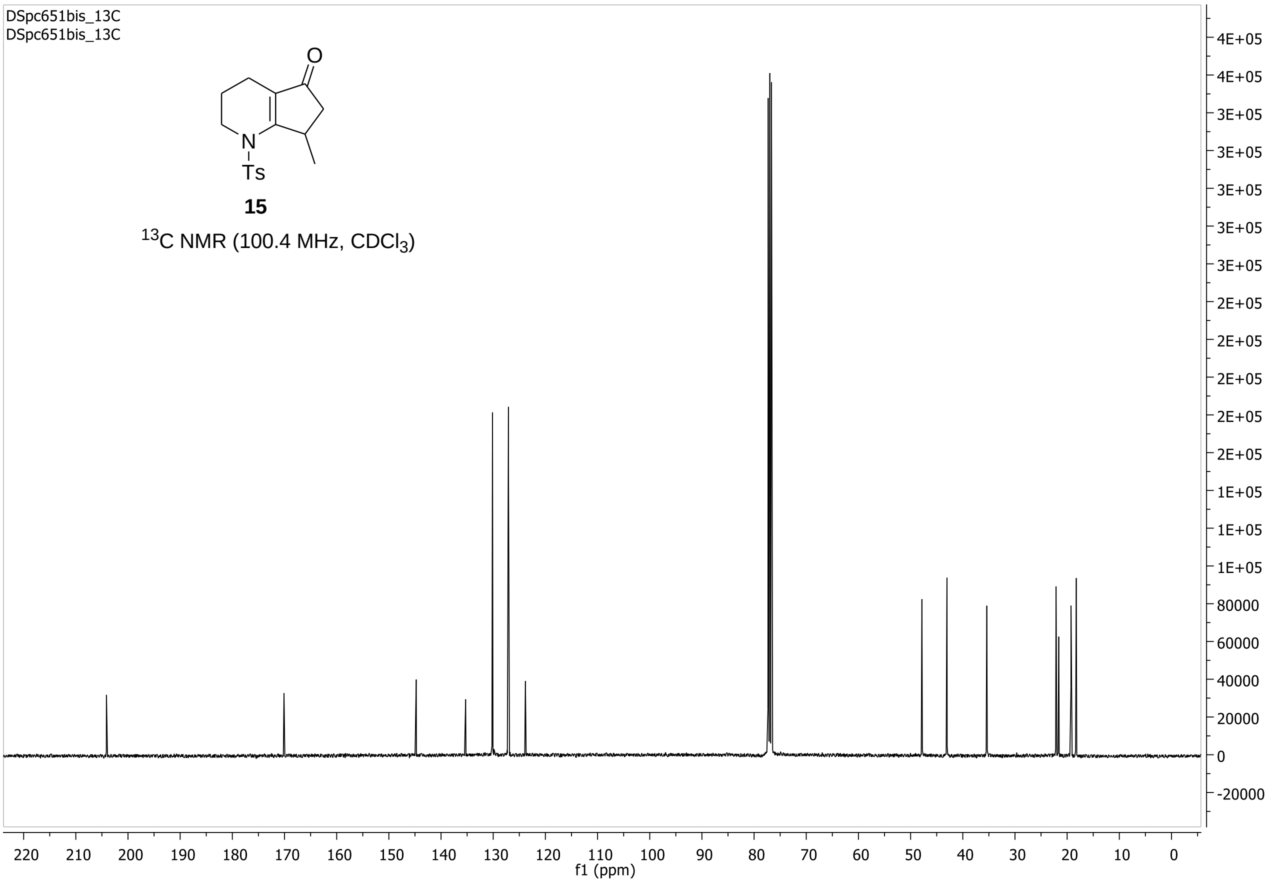
DSpc651bis_13C
DSpc651bis_13C
O
N
Ts
15
13C NMR (100.4 MHz, CDCl3)
f1 (ppm)

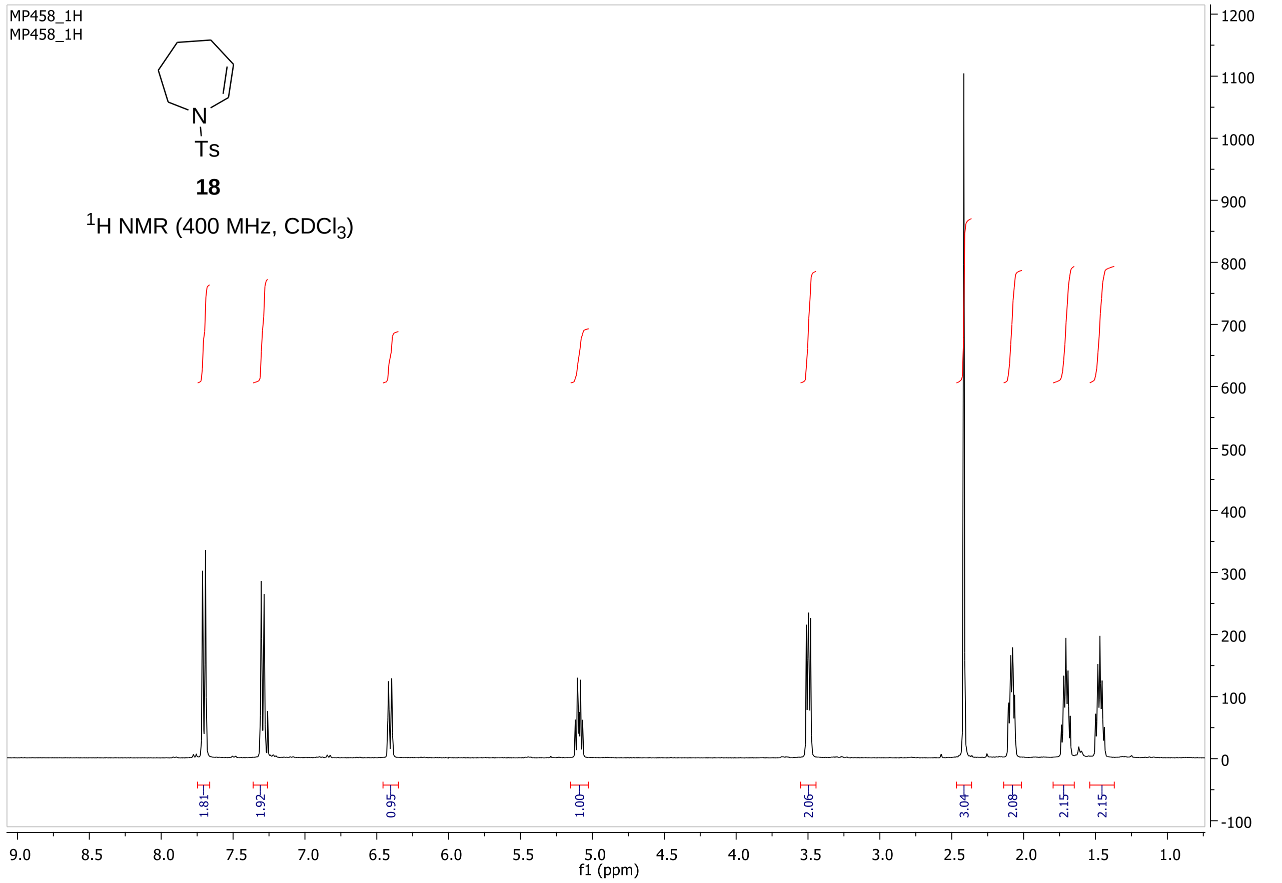
MP458_1H
MP458_1H
N
Ts
18
1H NMR (400 MHz, CDCl3)
1.81
1.92
0.95
1.00
2.06
3.04
2.08
2.15
2.15
f1 (ppm)

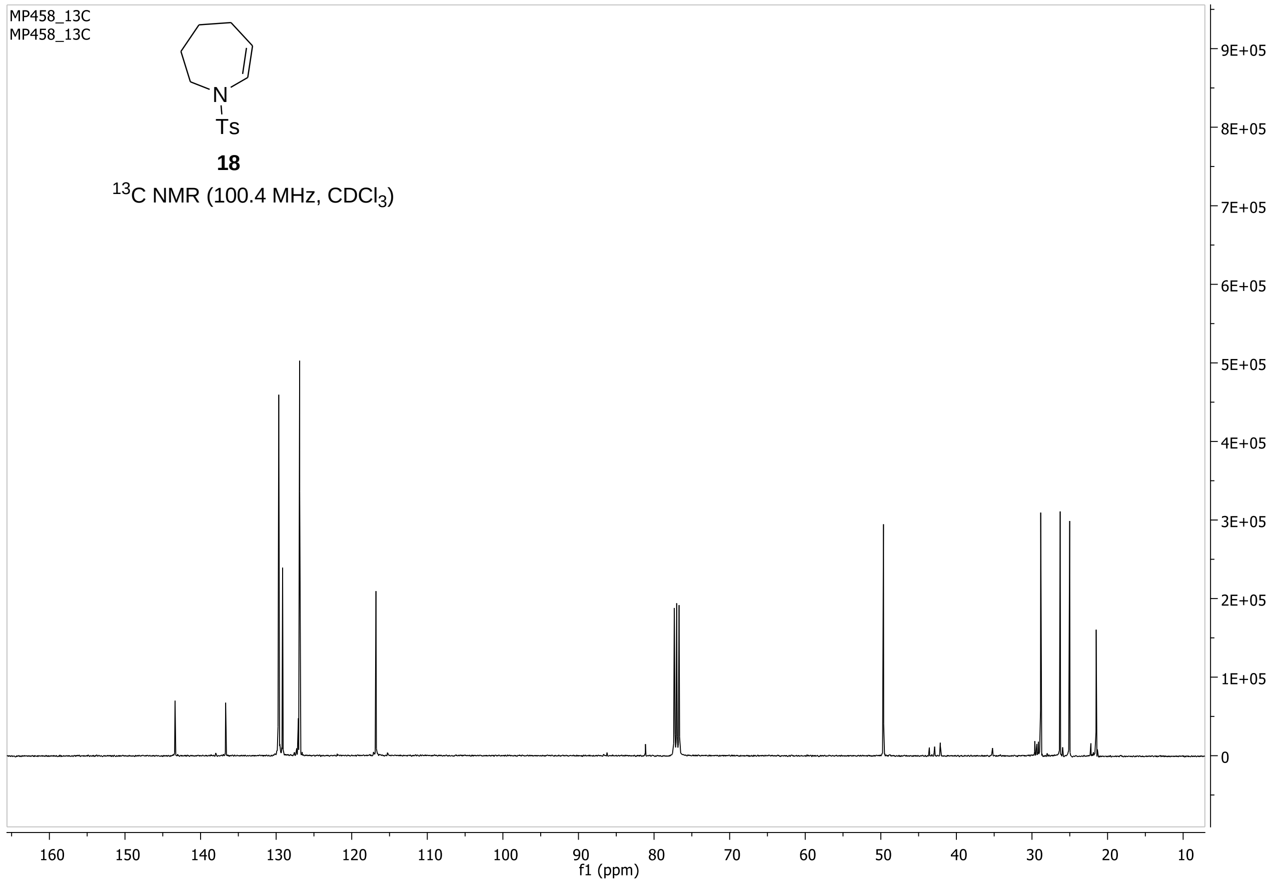
MP458_13C
MP458_13C
N
Ts
18
13C NMR (100.4 MHz, CDCl3)
f1 (ppm)

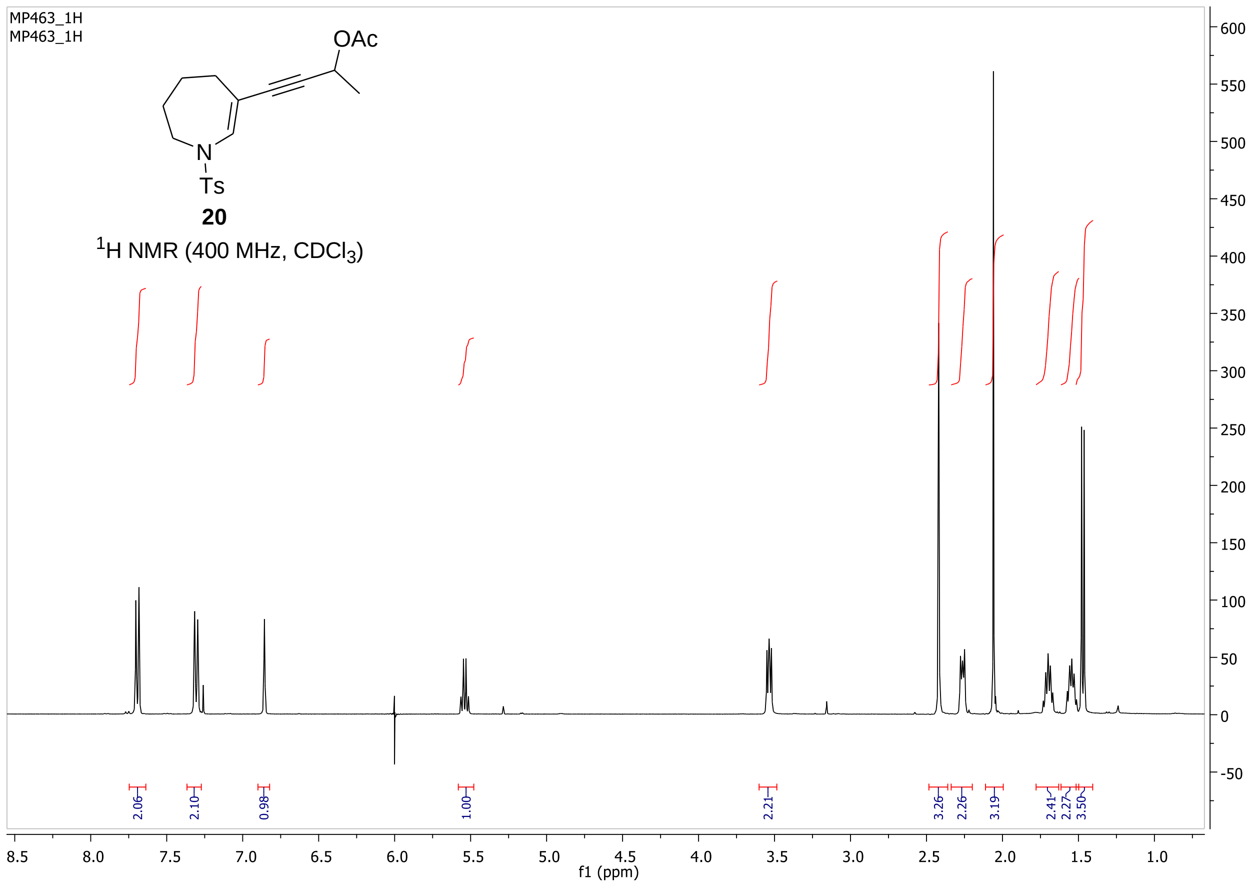
MP463_1H
MP463_1H
OAc
N
Ts
20
^{1}H NMR (400 MHz, $CDCl_3$)
2.06
2.10
0.98
1.00
2.21
3.26
2.26
3.19
2.41
2.27
3.50
f1 (ppm)

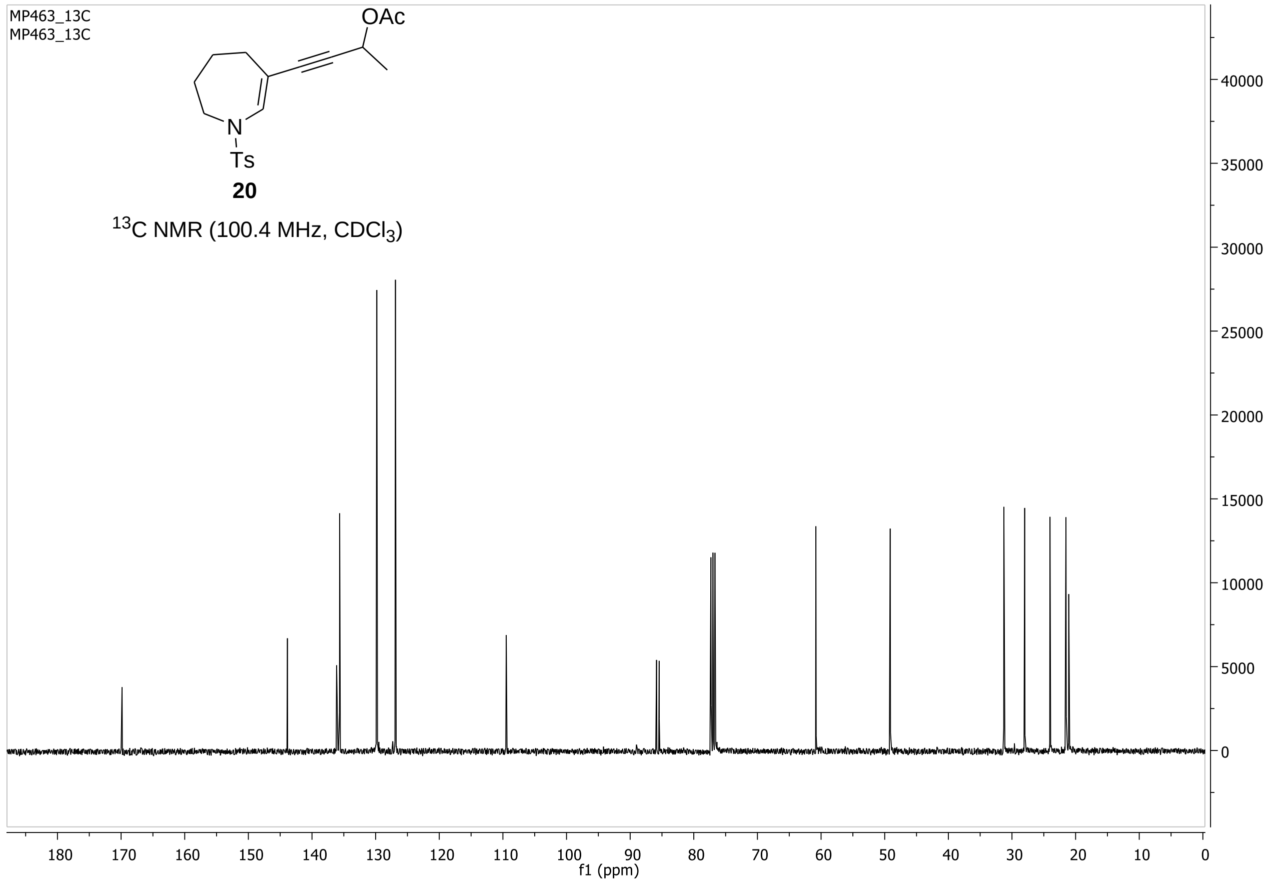
MP463_13C
MP463_13C
OAc
N
Ts
20
^{13}C NMR (100.4 MHz, $CDCl_3$)
f1 (ppm)

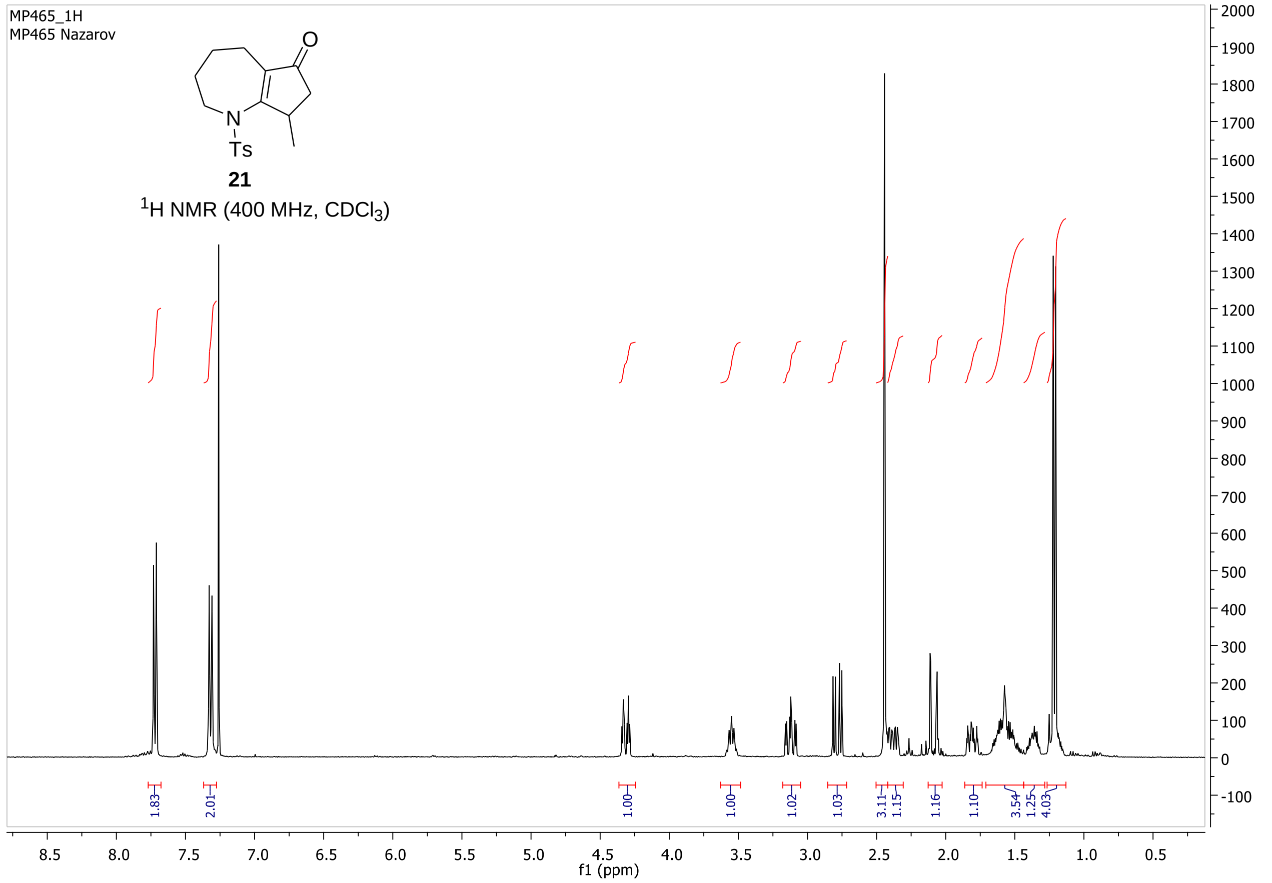
MP465_1H
MP465 Nazarov
O
N
Ts
21
^{1}H NMR (400 MHz, $CDCl_3$)
1.83
2.01
1.00
1.00
1.02
1.03
3.11
1.15
1.16
1.10
3.54
1.25
4.03
f1 (ppm)

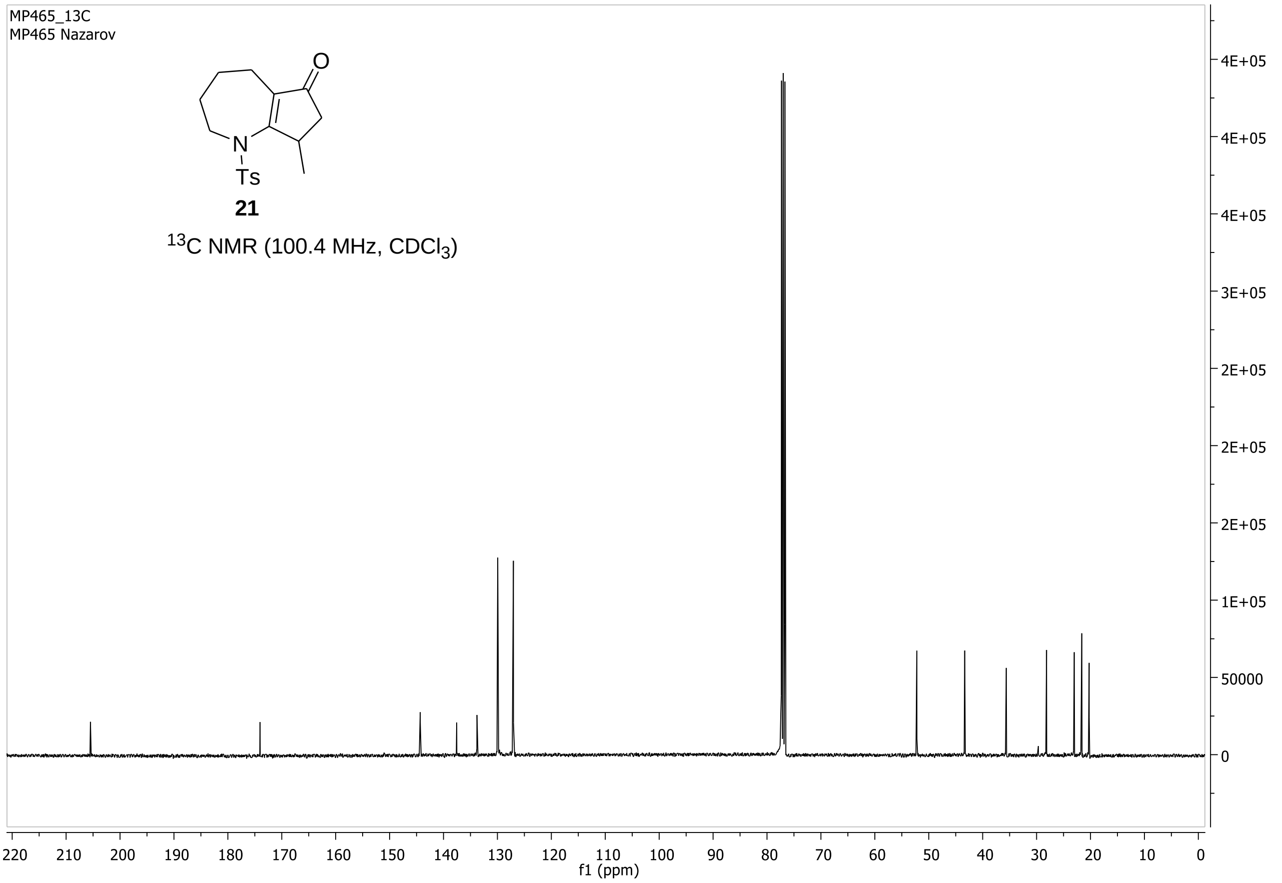
MP465_13C
MP465 Nazarov
O
N
Ts
21
^{13}C NMR (100.4 MHz, $CDCl_3$)
f1 (ppm)
